# Maximum likelihood inference of time-scaled cell lineage trees with mixed-type missing data using LAML

**DOI:** 10.1186/s13059-025-03649-9

**Published:** 2025-07-02

**Authors:** Gillian Chu, Uyen Mai, Henri Schmidt, Benjamin J. Raphael

**Affiliations:** Department of Computer Science, Princeton University, 08540 Princeton, NJ USA

**Keywords:** Lineage tracing, Cell lineage, Phylogeny, Time-resolved, Maximum likelihood using LAML

## Abstract

Dynamic lineage tracing technologies combine genome editing with single-cell sequencing to track cell divisions. We introduce Lineage Analysis via Maximum Likelihood (LAML) to infer a maximum likelihood time-resolved cell lineage tree under the Probabilistic Mixed-type Missing model, which we derive to describe key features of dynamic lineage tracing systems. LAML produces accurate tree topologies with branch lengths representing experimental time between ancestral cells. LAML outperforms existing methods in terms of accuracy and scalability on simulated data, and calculates the timing of cell migrations to metastatic sites in a mouse model of lung adenocarcinoma, revealing distinct epochs of metastasis progression.

**Supplementary information:**

The online version contains supplementary material available at 10.1186/s13059-025-03649-9.

## Background

Deriving the history of cell division and differentiation events that transform a collection of cells into a multicellular tissue or organism is of fundamental importance in developmental biology and cancer research [1–5]. The history of cell divisions during development is represented by a *cell lineage tree*, whose nodes are cells and whose edges indicate the parental relationships between cells. Beyond the ancestral relationships between cells, knowledge of the *timing* of key events, such as cell division, differentiation, and migration, is also essential to understanding development. Thus, it is desirable for the inferred cell lineage tree to include *time-resolved* branch lengths.

The first time-resolved cell lineage tree for a complete multi-cellular organism was the lineage tree for the 671 cells in *Caenorhabditis* *elegans*, which was derived through direct visual observation under microscopy [6, 7]. However, such direct experimental approaches are only feasible for small organisms of several hundreds of cells. Since the cell division process is typically not directly observable for higher organisms, *lineage tracing* is necessary to reconstruct the time-resolved cell lineage tree from cells sampled at the present time. Naturally occurring somatic mutations have been used as heritable markers for lineage tracing [7–9], but this approach is complicated by the low frequency of these mutations and their uneven distribution over the genome, requiring costly single cell whole genome sequencing to detect a sufficient number of somatic mutations [10].

In recent years, *dynamic lineage tracing* has overcome these challenges by using genome editing technologies to induce mutations at pre-defined genomic loci, or synthetic *target sites* [1, 11, 12]. These induced mutations are used as heritable markers for lineage tracing, avoiding the high cost of whole genome sequencing required to detect somatic mutations. Following genome editing, the target sites are measured using targeted amplicon sequencing [13], single-cell RNA sequencing [13, 14], imaging [11, 15], or other approaches. These mutations are then used to infer the cell lineage tree [14]. Many dynamic lineage tracing technologies have been developed, enabling lineage tracing for thousands of cells in vivo [4, 13, 16–25] (see [14] for a review). Recent advances in dynamic lineage tracing, such as CARLIN [21], iTracer [22], Chan et al. [4], Yang et al. [23], and Bolondi et al. [26], have leveraged CRISPR-based systems to control the strength of phylogenetic signal present in a cell culture by adjusting the number of target sites, the number of guide RNAs in the genome editing process, and other parameters. In such CRISPR-based lineage tracing systems, the genome editor is led to its target site by a guide RNA, where it creates an edit in the target site sequence. Once the target site has been edited, the guide RNA no longer matches the sequence at this target site and editing stops. Thus, a target site which has acquired an edit can no longer acquire any more edits—a property that we refer to as *non-modifiability*—nor can the edited site revert to the unedited state, a property we refer to as *irreversibility*.

Several computational methods have been developed to construct a cell lineage tree from dynamic lineage tracing data, including methods that were benchmarked in a DREAM challenge [15] such as Cassiopeia [27], DCLEAR [28], and AMbeRland [29], as well as more recent methods such as Startle [30], GAPML [31], TiDeTree [32], and others [33, 34]. We classify existing methods into two categories: non-probabilistic and probabilistic approaches. Non-probabilistic approaches comprise *distance-based methods* [28, 34] and *maximum parsimony methods* [27, 30]. These methods make few assumptions about the generative process of the dynamic lineage tracing system, making them robust to different types of lineage tracing data. However, without a temporal model of the genome editing system, non-probabilistic approaches can only estimate tree topologies *without time-resolved branch lengths*, precluding them from answering crucial research questions such as the timing of cell migration, fates, and fitness.

Probabilistic approaches to cell lineage tree estimation are related to statistical phylogenetics, which estimates phylogenetic trees of multiple species from their DNA or amino-acid sequences. These methods infer trees with time-resolved branch lengths. Popular software in statistical phylogenetics comprises maximum likelihood approaches (PhyML [35], IQ-Tree [36], RAxML [37], etc.) and Bayesian MCMC approaches (BEAST [38], Mr. Bayes [39], etc.). Despite conceptual similarities, these general-purpose phylogenetic methods do not capture key features of many dynamic lineage tracing systems, such as *irreversibility, non-modifiability*, and *heritable missing data*. Some attempts have been made to adapt statistical phylogenetics models to dynamic lineage tracing data, including GAPML [31] and TiDeTree [32]. However, both of these methods were designed for *older generations* of dynamic lineage tracing technologies that have few mutational states and/or low recording time. Additionally, they are very computationally intensive.

Consequently, there are currently no computational methods that can accurately infer a *time-resolved* cell lineage tree from the data generated by advanced dynamic lineage tracing systems such as Chan et al. [4], Yang et al. [23], and Bolondi et al. [26]. This is primarily due to two challenges with advanced dynamic lineage tracing data. First, many dynamic lineage tracing technologies produce *mixed-type missing data* from two distinct mechanisms: *heritable modifications* and *dropout*. Heritable missing data occurs for a number of reasons including epigenetic silencing of target sites (leading to lack of expression) and resection of several target sites at once. The latter source is more common in arrayed lineage tracing technologies such as GESTALT [13] which influenced the development of other technologies [4, 23, 26]. Dropout refers to unmeasured mutations due to technical issues in the RNA single-cell sequencing, such as low sequencing coverage. Dropout introduces non-heritable missing data. While dropout entries do not contain phylogenetic signal, heritable missing entries are phylogenetically informative and can improve tree inference. Importantly, the two missing data types appear identical in the observed data and no existing computational method distinguishes these types by leveraging the phylogenetic signal in heritable missing entries. Second, some dynamic lineage tracing technologies such as Chan et al. [4], Yang et al. [23], and Bolondi et al. [26] produce a *distinct set of possible genome edits* for every target site (i.e., heterogeneous target sites). This type of data is unconventional in statistical phylogenetics and has not been modeled in either species phylogenetics or single-cell lineage trees.

We present the first probabilistic model and maximum likelihood method for time-resolved cell lineage tree estimation from dynamic lineage tracing data. Our proposed Probabilistic Mixed-type Missing (PMM) model captures all key features of a dynamic lineage tracing system: (1) target sites which have acquired an edit can no longer acquire any more edits (non-modifiability), (2) due to the finite number of target sites, over time there is a decay in the rate of acquired mutations, (3) missing data can be classified into heritable and non-heritable types (mixed-type missing data), and (4) the set of possible genome edits is specific to each target site (heterogeneous target sites). Our maximum likelihood method, LAML (Lineage Analysis via Maximum Likelihood), jointly estimates the tree topology, time-scaled branch lengths, genome editing rate, heritable missing rate, and dropout probability under the PMM model. The method also imputes missing data and infers the maximum a posterior (MAP) estimate of the ancestral sequences. LAML is an iterative algorithm; in each iteration, LAML performs two steps: an EM algorithm for parameter estimation and tree rearrangements for topology search. Notably, the EM algorithm we derived under the PMM model is computationally efficient: in the case of no heritable missing data, we present a closed form solution, and in the general case, we present an efficient block coordinate ascent algorithm. In addition, we also extend PMM to the multi-progenitor PMM model, which applies to the case when several progenitors at the start of lineage tracing. Using the multi-progenitor PMM model, LAML leverages the progenitor labels (if they are available) to infer a *multi-progenitor time-resolved cell lineage tree*. Our method also imputes missing progenitor labels and detects missing progenitor cells.

We validate LAML on simulated data and on data from several model systems with a range of recent lineage tracing technologies: the KP-tracer mouse model of lung adenocarcinoma [23], the mouse model embryonic trunk-like structures [26], and the mouse embryo models traced with intMEMOIR [20]. Our results show that LAML outperforms existing methods on topology inference and infers plausible time-scaled branch lengths. Importantly, we demonstrate that the time-resolved cell lineage tree inferred by LAML enables mapping of metastasis events to real time—a novel application of dynamic lineage tracing. In the KP-tracer data of mouse lung adenocarcinoma, we discover 3 temporal epochs of metastasis progression in the lung adenocarcinoma model with distinct migration patterns and a metastasis burst at around month 2.

## Results

### LAML: lineage analysis using maximum likelihood

**Fig. 1:**
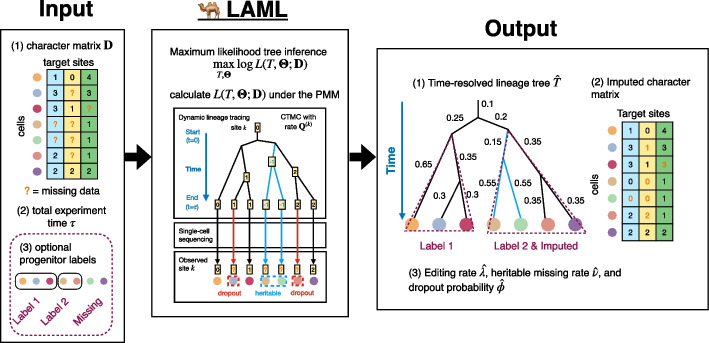
Overview of LAML. The input is a character matrix $\textbf{D}$ where “?” represents missing data. LAML performs maximum likelihood inference under the Probabilistic Mixed-type Missing (PMM) model and outputs three components: (1) a maximum likelihood time-resolved cell lineage tree $\hat{T}$, (2) an imputed character matrix, and (3) maximum likelihood estimates of other parameters in the PMM model, including the editing rate $\hat{\lambda }$, the heritable missing rate $\hat{\nu }$, and the dropout probability $\hat{\phi }$. The PMM generative model describes dynamic lineage tracing in two layers. Layer 1 (top) models the editing process and heritable transcriptional silencing events with a continuous-time Markov chain. After single-cell sequencing, transcriptionally silenced sites will appear as missing data. Layer 2 (bottom) models the introduction of stochastic missing data due to dropout during single-cell sequencing

Lineage Analysis via Maximum Likelihood (LAML) infers a maximum likelihood time-resolved cell lineage tree from single-cell dynamic lineage tracing data and the total time of the experiment (Fig. 1). The inputs to LAML are (1) the $N\times K$ character matrix $\textbf{D}$ where *N* is the number of sampled cells and *K* is the number of target sites, (2) the experiment time $\tau$, and (3) optionally the progenitor labels. The outputs to LAML are (1) the inferred time-resolved cell lineage tree, (2) the imputed character matrix, and (3) the inferred genome editing rate, heritable missing rate, and dropout probability.

LAML optimizes the likelihood of $\textbf{D}$ based on a generative probabilistic model—the *Probabilistic Mixed-type Missing (PMM)* model—which models the probability of **D** as a function of the unknown cell lineage tree, the genome editing rate, the heritable missing rate, and the dropout probability. In addition, some lineage tracing experiments attach a barcode, or *progenitor label* to individual cells at the start of the experiment and are used to indicate the founder cell of a cell population measured at the end of the experiment. These labels are frequently lost during the lineage tracing experiment. The PMM model also calculates the likelihood of the observed progenitor labels—when available—and LAML infers a maximum likelihood *multi-progenitor cell lineage tree* (see optional input in Fig. 1). LAML also infers the progenitor labels of the ancestral cells and imputes any missing progenitor labels.

#### The Probabilistic Mixed-type Missing (PMM) model

The PMM model consists of two layers that correspond to the editing of target sites (layer 1) and single-cell sequencing (layer 2). Layer 1 describes the genome editing process. Let $t_e$ be the length of a branch *e* in time units. We assume the genome editing system edits every site independently at a constant editing rate $\lambda$ and the process follows a continuous time Markov chain (CTMC) parameterized by $\lambda$ and the heritable missing rate $\nu$. Layer 2 describes the stochastic generation of dropout in single-cell RNA sequencing. After single-cell RNA sequencing, characters in the silent state (i.e., $-1$) will be observed in the missing state with probability 1, while all other states have some probability of being observed in the missing state depending on the *dropout probability* $\phi$. If the input includes the progenitor labels for the cells, we treat the progenitor label as a special site that has a distinct transition rate matrix in layer 1 but shares the same dropout matrix with other sites in layer 2. Thus, in addition to the tree topology *T*, the other parameters of the PMM model $\varvec{\Theta }$ are the branch length $t_e$ of every branch *e*, the editing rate $\lambda$, the heritable missing rate $\nu$, and the dropout probability $\phi$. As such, $\varvec{\Theta }=(\{t_e\},\lambda ,\nu ,\phi )$.

#### Maximum likelihood inference using LAML

We find a tree topology $\hat{T}$ and estimated values of the parameters $\hat{\varvec{\Theta }} = (\hat{\{t_e\}},\hat{\lambda }, \hat{\nu }, \hat{\phi })$ that maximize the log-likelihood function:

1 $$\begin{aligned} \hat{T}, \hat{\varvec{\Theta }} = \underset{T,\varvec{\Theta }}{\text {argmax}}\, \log {L(T,\varvec{\Theta };{\textbf {D}})}, \end{aligned}$$

such that the sum of branches on each root-to-tip path in the inferred tree equals the input experiment time $\tau$ (i.e., the time-resolved cell lineage tree is ultrametric and has height $\tau$). In other words, the following constraint must be satisfied for every leaf node *w*:

2 $$\begin{aligned} \sum \limits _{e \in \textrm{Path}(r_{\hat{T}},w)}\hat{t_e} = \tau , \text {for all}\ w\ \in \mathscr {L}_T, \end{aligned}$$

where $\textrm{Path}(r_T,w)$ denotes the path (a list of edges) from the root $r_T$ to *w*.

As maximum likelihood inference of a phylogenetic tree is NP-hard [40], in LAML we propose an efficient heuristic algorithm to find the maximum likelihood tree under the PMM model (Fig. 1). First, we arbitrarily initialize *T* and $\varvec{\Theta }$, then we iterate over the following steps until convergence: (i) propose a new tree topology $T'$ using nearest neighbor interchanges (NNI); (ii) compute the maximum likelihood estimate $\hat{\varvec{\Theta }}$ on the fixed tree topology $T'$ using EM algorithm; and (iii) accept/reject the new topology according to simulated annealing. Importantly, our newly derived EM algorithm for step (ii) is efficient. We derive a linear time algorithm to compute the E-step exactly and present a closed-form solution for the M-step in the case of no heritable missing data; our closed-form solution for the M-step can also be computed in linear time. When there is heritable missing data, we show that the optimization problem in the M-step is *separately* convex on multiple blocks of the parameters $\varvec{\Theta }$; in this case we use block coordinate ascent to efficiently solve the M-step. Refer to the Methods section for more details about the PMM model and the LAML algorithm.

LAML solves two maximum likelihood inference problems: (1) estimate maximum likelihood parameters (time-resolved branch lengths, the heritable missing rate, and the dropout probability) on a fixed tree topology and (2) infer a maximum likelihood tree topology as well as the parameters. We detail command-line functionality for both in the tutorial.

### Evaluation on simulated data

We compare LAML to eight other algorithms for constructing trees from lineage tracing data: Cassiopeia-Greedy [27], Cassiopeia-Hybrid [27], Cassiopeia-ILP [27], TiDeTree [32], Neighbor-Joining [41] (implemented in Cassiopeia [27]), DCLEAR [28], and Startle-NNI [30]—a maximum-parsimony method. We evaluate these methods on simulated trees with 1024 leaves (i.e., 10 cell generations) generated using the birth-only process implemented in Cassiopeia [27] as described in Simulation procedure section of Methods section. Given 24 h and 4 GB of memory, Cassiopeia-Hybrid, Cassiopeia-ILP, and TiDeTree were unable to consistently infer trees with 250 leaves (Additional file S1: Figs. S1 and S2). We omit these methods from the following discussion and refer the reader to Additional file S1.7 for further details. We fix the overall missing data percent to $25\%$, while creating five model conditions with different proportions of heritable and non-heritable missing data (Table 1). For example, the model condition h0d100 has all missing data were generated by dropout, while h25d75 has 25% heritable missing and 75% dropout out, etc. In addition, we also create ten sets of mutation alphabet hyperparameters. For each of five model conditions and ten sets of mutation alphabet hyperparameters, we created five replicates each, for a total of 250 simulated datasets.

**Table 1:** Simulated dataset under five model conditions

Model condition	Heritable percent	Dropout percent	Heritable rate ($\varvec{\nu }$)	Dropout probability ($\varvec{\phi }$)
h0d100	0%	100%	0	0.25
h25d75	25%	75%	0.065	0.2
h50d50	50%	50%	0.134	0.143
h75d25	75%	25%	0.208	0.077
h100d0	100%	0%	0.288	0

All model conditions were simulated with $25\%$ missing data; the attribution of the missing entries to heritable missing versus dropout depending on the model condition

#### LAML infers accurate time-resolved cell lineage trees

**Fig. 2:**
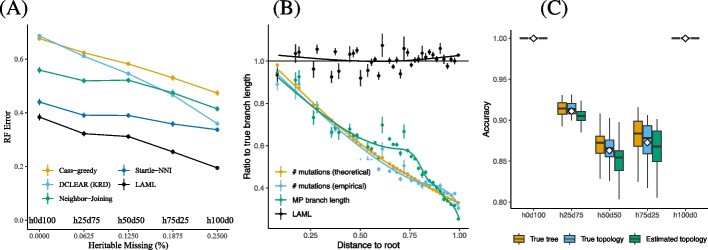
Comparison of LAML and other methods on simulated data. **A** Evaluation of tree topologies using normalized RF error under 5 different percentages of heritable (“h”) and dropout (“d”) of missing data. **B** The ratio of the estimated to true branch lengths using the true tree topologies as input. Black line shows the perfect fit where estimate/true = 1. The *x*-axis is discretized into 50 bins and the estimated branch length by each method (or measure) is averaged for each bin, shown with error bar. **C** Accuracy of LAML on imputing missing data, shown for 3 scenarios: (red) true tree topology $T_{\text {true}}$ with true parameters $\varvec{\Theta }_{\text {true}}$ given; (green) the true topology $T_{\text {true}}$ is given, the MLE $\hat{\varvec{\Theta }}$ is estimated by LAML; (blue) both *T* and $\hat{\varvec{\Theta }}$ are estimated by LAML

The cell lineage trees inferred by LAML have more accurate topologies than trees inferred by Neighbor-Joining, Cassiopeia, DCLEAR, or Startle, with greater advantages evidenced when more heritable missing data is present. Specifically, we observe that LAML infers trees with lower Robinson-Foulds (RF) error (i.e., normalized RF distance between true and estimated trees; Methods: Evaluation metrics section) than the trees inferred by other methods (Fig. 2A) Moreover, while the RF error decreases for all methods as the the rates of heritable missing increases, the improvement LAML achieves over other methods also increases. For instance, compared to Startle—the second best method—the RF error of LAML is lower by 0.06 at h0d100 ($0\%$ heritable missing) and lower by 0.14 at h100d0 ($100\%$ heritable missing), suggesting that LAML better leverages the phylogenetic signal in heritable missing data than the other methods.

As a positive control, we also evaluate each method by its ability to optimize its own objective (Additional file S1: Table S1 and Fig. S4). As expected, LAML has the highest log-likelihood, while Startle-NNI (a maximum parsimony method) typically has the lowest weighted parsimony cost. However, in $16\%$ of the cases the tree inferred by LAML has a lower weighted parsimony cost than the Startle-NNI tree. One possible reason is because Startle-NNI is a heuristic algorithm that does not always find the true optimum and since there is a high correlation between weighted parsimony and log-likelihood, LAML will typically also produce trees achieving a good weighted parsimony score.

LAML also produces highly accurate branch lengths, substantially outperforming methods based on maximum parsimony. The average ratio of estimated-to-true branch lengths for LAML is 1.02 compared to 0.39 for branch lengths estimated from maximum parsimony (computed using Sankoff’s algorithm [42]; see Methods for details). Importantly, the accuracy ratio for LAML remains roughly constant as a function of the branch’s distance to the root (Fig. 2B, black line), showing that LAML yields unbiased estimates of branch lengths. In contrast, the accuracy of the MP branch lengths decays with distance to the root, with branch lengths near the tips of the trees severely underestimated (Fig. 2B, yellow and blue lines). In addition, since the time-resolved branch lengths $\delta _e$ are related to the genome editing rate $\lambda$ in the PMM model by $\delta _e=\lambda t_e$, we also observed that the editing rate $\hat{\lambda } = 0.094$ inferred by LAML is very close to the simulated editing rate $\lambda =0.095$.

We further demonstrate the bias of MP branch lengths both theoretically and empirically using simulated data, demonstrating that using MP branch lengths in lineage trees is ill-advised. The bias of MP branch lengths is the result of using mutation counts as a proxy to measure branch lengths. Because of the decay in the number of available target sites, *mutation counts are not proportional to branch lengths in time units* under the PMM model. Instead, the ratio of mutation counts to time-unit branch lengths actually decreases over time. Thus, the bias in MP branch lengths is *systematic*: the method always underestimates the branch lengths, and branches further from the root are more severely underestimated. To show this fact, we compute both the theoretical and empirical mutation counts under the PMM model and added them as the two alternative means for branch length computation. Under the PMM model, the theoretical expected mutation count of each branch $e=(u,v)$ is $(1-e^{-\delta _e(1+\nu )})e^{-d(r_T,u)(1+\nu )}$, where $d(r_T,u)$ denotes the distance from the root $r_T$ to *u*. (We omit a proof here, but the derivation is straight-forward using Eq. 4). The empirical mutation counts are simply collected from simulated data. We also divide both the theoretical and empirical mutation counts by $\lambda$ to “convert” them to time units. Indeed, we observe that both the theoretical and empirical mutation counts are systematically biased if used to measure time—branches further from the root are more severely underestimated (Fig. 2B, yellow and blue lines). Thus, while it has become a common practice to use the MP branch lengths on the MP cell lineage trees on downstream analyses of lineage tracing [23], our results show that such a practice needs careful consideration, as the MP branch lengths are a poor proxy for time.

#### LAML accurately infers heritable missing rate and dropout probability

LAML accurately and robustly estimates missing data rates and imputes missing entries in the character matrix over all five model conditions, even in the presence of errors in tree topology and branch length (Additional file S1: Fig. S3A). Still, more accurate tree topologies help reduce errors in these estimates. For instance, the values we estimate on the LAML tree topology (which achieved the lowest RF error) have the lowest RMSE, pointing out the advantage of using LAML to infer both the tree topology and other parameters, instead of using an a less accurate topology. LAML also achieves a nearly perfect imputation accuracy when all missing entries (which account for $25\%$ of the whole data) arise solely from dropout (heritable: $0\%$, dropout: $100\%$, denoted as h0d100) or solely from heritable missing data (heritable: $100\%$, dropout: $0\%$, denoted as h100d0). When there is mixed-type missing data, LAML is still highly accurate with at least $85\%$ accuracy in all the model conditions that we tested (Fig. 2C). Interestingly, the imputation accuracy is higher with lower variance on data where heritable missing events account for fewer of the missing entries (h25d75), compared to when heritable missing events account for more of the missing entries (h75d25), suggesting that LAML correctly classifies more dropout missing events than heritable missing events, possibly due to an overestimation of dropout. In all model conditions, LAML’s missing data imputation is robust against errors in tree topology and branch length ($\le 2\%$ difference when using the true versus estimated trees).

#### Scalability

We found that LAML produces trees with lower error more quickly than current methods while consuming comparable computational resources (Additional file S1: Fig. S7, Section S1.7.1). We leverage the numerical computing library Jax [43] in our implementation of the EM algorithm for optimizing parameters on a fixed tree. On a dataset of 5000 cells, only LAML, Cassiopeia-Greedy, and Neighbor-Joining produced results in less than 24 h of runtime, with LAML consuming less memory than Neighbor-Joining (implemented in the Cassiopeia suite, Additional file S1: Fig. S7). To the best of our knowledge, no method has proven scalable enough to be evaluated on datasets of this scale.

The overall runtime of the EM algorithm for parameter estimation depends on the number of iterations, which we found to be less than 15 iterations on average even for trees with up to 5000 cells (Additional file S1: Fig. S8). We observed that the runtime of the EM algorithm was largely unaffected by the alphabet size.

### Mouse lung adenocarcinoma (KP-Tracer) data

We analyze a lineage tracing dataset from a mouse model of metastatic lung adenocarcinoma [23], which following the original publication we will refer to as the KP-tracer data. We show below that LAML not only infers a cell lineage tree that is *more plausible* than the published trees inferred by Cassiopeia [23] and Startle [30], but also our time-resolved branch lengths enable the study of *metastasis progression*, a novel application of the cell lineage tree.

#### Analysis of branch length estimation on six representative samples

We compare the time-resolved branch lengths inferred by LAML with the MP branch lengths inferred by Maximum Parsimony methods on six representative samples from KP-tracer and find that LAML produces more accurate branch lengths. The six representative samples (3432_NT_T1, 3435_NT_T4, 3520_NT_T1, 3703_NT_T1, 3703_NT_T2, and 3703_NT_T3) were selected based on the following two criteria: high number of target sites (ranging from 29 to 73) and moderate numbers of cells (29 to 294). For this evaluation, we gave all methods the published tree topologies and only estimate branch lengths.

**Fig. 3:**
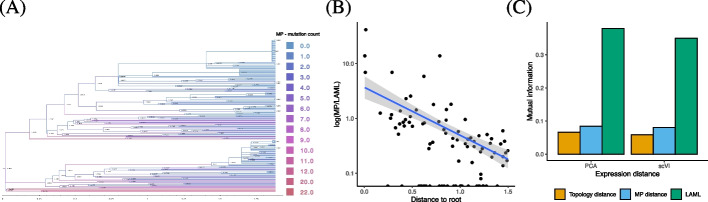
Evaluation of branch lengths on the cell lineage tree for sample 3432_NT_T1 from the KP-Tracer mouse lung adenocarcinoma lineage tracing study. **A** The tree topology published in [23] with branch lengths estimated by LAML indicated on each edge. Edge colors indicate the number of mutations inferred by maximum parsimony. **B** The log-ratio between the branch length estimates by maximum parsimony and by LAML as a function of the distance between the root node and the end of the branch. **C** Mutual information between gene expression and phylogenetic distances. Gene expression distance is computed in latent space. Phylogenetic distance is computed on branch lengths that were estimated by LAML, computed from maximum parsimony (MP) distance, or set to equal unit length 1 (topological distance)

We observe that the MP and LAML branch lengths are not proportional (Fig. 3A). Importantly, there is a decrease in the ratio of the maximum parsimony branch length to the branch inferred by LAML (MP/LAML) with the distance to the root (Fig. 3B)—a pattern that is consistent with the results shown on simulated data (Fig. 2B) where the MP branch lengths decrease with distance to the root while LAML branch length estimates do not. This same pattern is observed in all of the six tested samples (Additional file S1: Fig. S10A).

We also find that the LAML’s branch lengths are more plausible than the MP branch lengths according to the gene expression measured in each cell (Fig. 3C). Specifically, we compared a phylogenetic distance between each pair of cells on the tree to a gene expression distance between the gene expression vector of the cells. We define the *phylogenetic distance* between a pair of cells as the sum of the branch lengths on the unique path in the tree between the leaf nodes corresponding to these cells. Note that every branch length estimation method produces a different set of pairwise phylogenetic distances. We define *gene expression distance* between a pair of cells as the Euclidean distance between their projected gene vectors using either PCA or scVI [44]). For each method that estimates branch length, we compute the *mutual information* between the phylogenetic distances and the gene expression distance, with higher mutual information indicating better agreement between the phylogenetic distances and gene expression distances. As a baseline, we also compare with the topological distance, where all branches are set to length 1. The branch lengths estimated by LAML have substantially higher agreement with the gene expression data (as measured via mutual information) than the branch lengths estimated by maximum parsimony or topological distance. Interestingly, the mutual information of the MP branch lengths to gene expression is barely higher than the baseline method that uses topological branch lengths. A similar result is observed on the other five samples (Additional file S1: Fig. S10B). Further details about data processing and evaluation metrics are in Additional file S1.

The maximum parsimony objective produces branch lengths which minimize overall evolutionary change, whereas the LAML objective finds branch lengths which maximize the likelihood of generating the observed data. Since both the allelic distance and the branch lengths inferred by maximum parsimony (MP) are based on minimizing changes, one would expect that these quantities are highly correlated. However, we observe that the allelic distances exhibit higher correlation with the branch lengths inferred by LAML (in mutation units) than with the MP branch lengths on 4 out of the 6 samples (Additional file S1: Table S2 and Section S1.8.1). Thus, the branch lengths inferred by LAML maintain comparable correlation with the allelic distances while greatly improving the concordance with gene expression.

#### Analysis of lineage tree topology on the largest sample

**Fig. 4:**
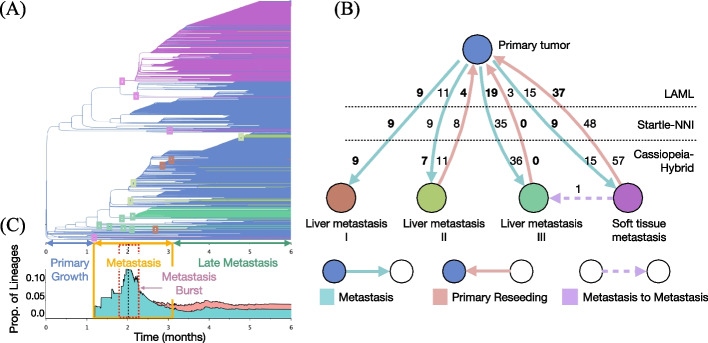
Migrations between anatomical sites in mouse lung adenocarcinoma sample 3724_NT_T1_All. **A** LAML’s estimated phylogeny on 1461 cells (de-duplicated data). Time-scaled branches are colored by the inferred anatomical location of incident child node. Small boxes indicate metastasis events. Time is divided into three epochs: primary growth (blue), metastasis (yellow), and late metastasis (green). Red dashed lines mark the metastasis burst and the black dashed line marks the peak of metastasis burst. **B** Migration graph inferred from the trees estimated using Cassiopeia-Hybrid, Startle-NNI, and LAML. Nodes represent the anatomical sites, arrows represent migration events, and numbers represent the number of migration events inferred from each estimated tree. Bold indicates the lowest number for each migration event. Note that the metastasis to metastasis event from soft tissue to liver III appears in all three methods. **C** Stacked area chart representing the proportion of lineages (branches) annotated as metastasis (aqua), reseeding (red), and metastasis to metastasis (purple) at each time point

We perform another evaluation of LAML, Cassiopeia, and Startle by comparing the number and pattern of cellular migrations that are inferred on the trees obtained by each method on the largest KP-Tracer sample 3724_NT_T1_All. This sample has 21,108 cells in total, but many of the cells have identical CRISPR/Cas9 sequences, resulting in 1461 unique cells after deduplication. These cells were sequenced from tumor samples from three anatomical locations in the mouse: the primary lung tumor, three liver metastases, and a soft tissue metastasis. LAML produces a very different tree topology (Fig. 4A) from the published trees inferred by Cassiopeia-Hybrid [23] (normalized RF 0.81) and Startle-NNI [30] (normalized RF 0.58), but maintains high correlation indel allelic distance and LAML’s inferred phylogenetic distances (in mutation units) (LAML: 0.920, Startle-NNI: 0.930, Cassiopeia-Hybrid: 0.907, Mantel test *p *value $\le 0.002$, see Additional file S1: Section S1.8.1 for further details). We evaluate the trees inferred by each method by examining the minimum number of migrations between anatomical locations that is inferred on each tree, following the procedure described in [30] and [45]. Specifically, we assume that a cell leaving a tumor at one location and traveling through the bloodstream to seed (or join) a metastasis at another anatomical site is a rare event. Under this assumption, a lineage tree with fewer total migrations is more plausible.

We compute the minimum number of migrations in a cell lineage tree by finding a maximum parsimony labeling of the ancestral nodes of the tree by anatomical locations and summarize the pattern of migrations by the *migration graph* described in [45]. Specifically, we use Sankoff’s algorithm [42] to compute the labeling that minimizes the total number of migrations—or changes in anatomical location—along the edges of the tree (as described in [30, 45]). The migration graph is a directed graph whose *nodes are anatomical sites*, *edges indicate migration events*, and *edge weights show the number of times a migration event happened* on a maximum parsimony labeling of the ancestral nodes of the tree (Fig. 4B). Migration events are classified into three types: primary tumor to a non-primary anatomical site (*metastasis*), non-primary anatomical site to the primary tumor (*reseeding*), and non-primary anatomical site to a different non-primary anatomical site (*metastasis to metastasis*). Similar to [30], we define the *migration cost* as the sum of edge weights in the migration graph and use it as an evaluation metric for lineage tree topology. We also define the *reseeding cost* as the total number of reseeding events and use it as a secondary evaluation metric. Lower values of the migration and reseeding costs are more plausible under the assumptions that both migrations between distant anatomical sites and migrations back to a previous tumor are rare. See Additional file S1: Section S1.3 for further information.

The migration graph computed from the LAML tree is more plausible than those computed from Cassiopeia and Startle trees in several respects. First, LAML achieves both the lowest migration cost and lowest reseeding cost (Fig. 4B and Additional file S1: Table S3). Second, compared to both Cassiopeia and Startle, LAML has the lowest *reseeding to seeding ratio* for liver metastasis I and II. Finally, LAML has the lowest total number of migration events for liver metastasis III and soft tissue metastasis.

Although LAML produces a very different tree topology (Fig. 4A) from the published trees inferred by Cassiopeia-Hybrid [27] (normalized RF 0.81) and Startle-NNI [30] (normalized RF 0.58), the correlation between the indel allelic distance and LAML’s inferred phylogenetic distances (in mutation units) are comparable to the correlations obtained by Cassiopeia-Hybrid or Startle-NNI phylogenetic distances (LAML: 0.920, Startle-NNI: 0.930, Cassiopeia-Hybrid: 0.907, Mantel test *p *value $\le 0.002$, see Additional file S1: Section S1.8.1 for further details). We further explored how well the indels support the inferred metastasis events. Recall that we define a metastasis event as a branch where the anatomical labeling of the parent node is the primary tumor, and the anatomical labeling of the child node is a different anatomical site. We refer to the extant cells under the child node as the metastasis clade and the other cells under the parent node as the sibling group. We can therefore evaluate two tree topologies by the indel support for the metastasis clades under the inferred metastasis events. Since the migration cost is lower in the LAML tree, as expected we find that the metastasis clades are on average larger in the LAML tree than in the published Cassiopeia-Hybrid tree (LAML: 19.852, Cass-Hybrid: 10.194 average extant cells per metastasis clade) and that metastasis clades consisting of more than 2 cells have lower allelic distance in the LAML tree than they do in the Cassiopeia-Hybrid tree (LAML: 0.186, Cassiopeia-Hybrid: 0.242 normalized allelic distance).

At the same time, the migration graphs computed from the three trees agree on a few key properties of metastatic spread, even though the trees from the three methods are different (according to RF distance). First, a single migration event from soft tissue metastasis to liver metastasis III is detected by all methods, and no other migration events between other pairs of non-primary sites is detected. Second, all trees agree on a high number of reseeding events for soft tissue metastasis, a result that is consistent with local spread of cancerous cells prior to metastasis through the blood or lymphatic system to distant anatomical sites [46].

#### Timing metastasis progression with time-resolved branch lengths

We illustrate the advantages of LAML’s time-scaled branch lengths by examining the timing of cellular migrations between the primary tumor and the multiple anatomical sites. An analysis of the timing of metastasis was not performed in the original KP-tracer publication [23] since the lineage trees were inferred by Cassiopeia and thus do not have time-scaled branch lengths.

The time-resolved cell lineage tree inferred by LAML reveals three epochs of metastasis progression with distinct patterns of cell migration (Fig. 4C): primary growth (month 0–1), metastasis (month 1–3), and late metastasis (month 3–6). In the primary growth epoch, cells grow and divide within the primary tumor but do not migrate (i.e., no metastasis) (Fig. 4C). The metastasis epoch starts at around month 1, with a gradual increase in the number of metastasis events and lasts until around month 2, followed by a sudden surge (i.e., metastasis burst) and a later gradual reduction until month 3 (Fig. 4C, aqua area plot). The first metastasis event is a soft tissue metastasis (around the end of month 1), which is consistent with previous reports that local spread of tumors into surrounding soft tissue is expected to be an early migration event [46] (Fig. 4C, red area plot). Notably, in month 2, there is a surge of metastases (i.e., the metastasis burst window, see Fig. 4C) that lasts about half a month (from month 1.75 to 2.25). We observe that during metastasis burst, the rate of metastasis (i.e., the number of metastases per cell lineage per month) increases more than 30-fold (from a background rate of 0.003 to a rate of 0.113) and during this short time there are metastases to multiple anatomical sites. We also explore the largest metastasis event in the LAML tree, which occurs over the 1.810 to 2.178 month period and overlaps with the “metastasis burst” time period. Extant cells inferred to have metastasized share a key indel in the third target site (Additional file S1: Section S1.8.1). After the metastasis burst, there is a gradual decrease in the number of migrations, until the beginning of month 3 when the number reaches a stable state. We mark this time point as the start of the late metastasis epoch. In late metastasis, there is a stable proportion of lineages with migrations at every time point. In addition, we also observe reseeding events during this epoch, where cells are inferred as migrating back to the primary tumor from distant metastases [45]. Interestingly, the ratio of reseeding to all migration events is maintained at a constant value of about 0.4 throughout the late metastasis epoch. Because there are a low number of unmodified target sites from month 3 forward ($\le 3$), the phylogenetic signal is weaker during this epoch, and thus we expect higher uncertainty in the inference of both tree branch lengths and metastasis events.

Finally, we analyze the missing data rates inferred by LAML on this largest sample of the dataset. According to our estimates, this sample has mixed-type missing data: dropout probability $\phi = 0.043$ and heritable missing rate $\nu =0.010$. Among the 753 missing entries in the data, LAML imputes 578 entries to be dropout and 175 entries to be heritable missing. The 175 heritable missing entries are attributed to 80 heritable missing events, with each event introducing 2 to 4 missing entries to the character matrix. Interestingly, the signal from heritable missing data is lost when we estimate missing data rates and branch lengths using the Startle-NNI tree topology ($\phi = 0.057$, $\nu =0$). This is not surprising since Startle-NNI does not model heritable silencing events. Instead, the Startle-NNI tree separates cells with similar missing data patterns, explaining the lineage data in these cells with multiple dropout events. Thus, the Startle-NNI tree topology yields an inflated estimate of the dropout probability compared to the LAML tree topology which explains the missing data by shared ancestral silencing events.

### Inference of progenitor labels in mouse trunk-like structures (TLS) dataset

We apply LAML to infer cell lineage trees from lineage tracing data from embryonic trunk-like structures (TLS), a model of mouse embryogenesis [26]. In this dataset, a founder population of progenitor cells are labeled with a unique lentiviral barcode, which we will refer to as the progenitor label, prior to lineage tracing. At the conclusion of the experiment, both the lentiviral barcodes and lineage barcodes are sequenced, with the lentiviral barcode indicating the ancestral progenitor cell. We refer to these as the *progenitor labels* of the cells. However, as the progenitor labeling process is imperfect, some of the sequenced cells are missing their progenitor labels. Importantly, it is unclear whether these cells share an ancestral cell which did not receive a progenitor label (heritable missing) or whether their progenitor labels were dropped in the sequencing process (dropout).

**Fig. 5:**
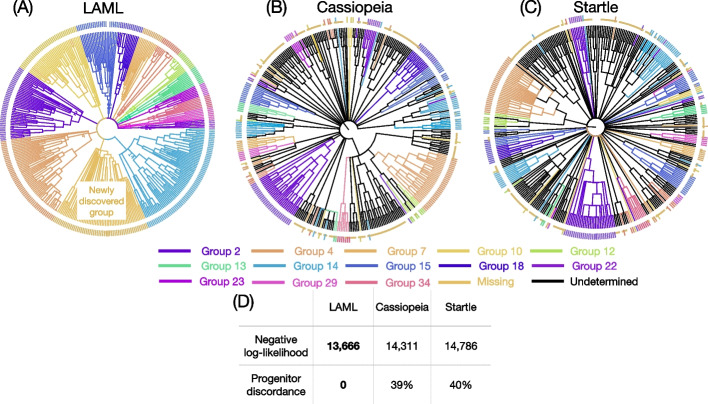
Cell lineage trees inferred for sample 12 of the TLS mouse embryo from [26]. **A** The LAML tree inferred using the multi-progenitor PMM model. Tree branches are colored by the monophyletic groups of progenitor labels. Note that by construction, the multi-progenitor LAML tree has no discordance with the progenitor labels; therefore, every clade branching out from the root belongs to a single progenitor group. **B** The trees inferred by Cassiopeia (published in [26]) and **C** Startle. Both trees are colored by the monophyletic groups of progenitor labels, with black branches indicating non-monophyletic groups due to discordance with the progenitor labels. **D** Negative log-likelihood and progenitor discordance of the three trees

Using the multi-progenitor PMM model (see Methods: The multi-progenitor PMM model and inference), LAML not only infers cell lineage trees but also imputes the missing progenitor labels. To the best of our knowledge, LAML is the only existing method that uses the progenitor labels to improve cell lineage tree inference. In the LAML cell lineage tree, each labeled (i.e., non-missing) progenitor serves as the common ancestor for a monophyletic clade attached to the root of the tree (Fig. 5A). In contrast, in the cell lineage tree from the original publication [26] (inferred by Cassiopeia) each progenitor does not correspond to a single clade (Fig. 5B). A similar result is seen on the tree inferred by Startle (NNI mode) where cells with the same progenitor label are distributed across multiple clades in the tree (Fig. 5C). We quantify this phenomenon using a measure called *progenitor discordance* which we define using the normalized triplet error, or proportion of cell triplets where cells sharing a progenitor label are not inferred to be in the same clade. More specifically, it is computed as $1-n_C/n_R$, where $n_R$ is the number of triplets where 2 cells have the same progenitor label and one cell has a different progenitor label, and $n_C$ is the number of such triplets where the outgroup cell is the one with a different progenitor label (see Methods: Evaluation metrics for more details). By construction, the multi-progenitor LAML trees have progenitor discordance of zero because LAML enforces that each progenitor label corresponds to a single clade. In contrast, Cassiopeia and Startle trees have much higher discordance because these methods do not enforce any restriction on the placement of cells by their progenitor labels (Fig. 5D and Additional file S1: Table S6).

A missing progenitor label in a cell could be the result of dropout during sequencing or a failure in the labeling of the ancestral progenitor cell. In the latter case, all cells in the clade of the lineage tree with the same progenitor cell will have a missing progenitor label. The LAML inferred tree distinguishes these two cases: some cells with missing progenitor labels are placed into the same clade as cells with the same progenitor label, suggesting that these cells’ progenitor labels were dropped in sequencing. Other cells with missing progenitor labels are grouped into monophyletic progenitor clades (Additional file S1: Section S1.9). Some of these clades have distinct indel patterns (see the “Newly discovered group” in Fig. 5 for an example) strongly suggesting that these cells are descended from a progenitor that did not receive a progenitor label. We refer to these as the missing label progenitors. We discover missing label progenitors in 5 of the 6 samples that we analyzed with LAML, and the number of newly discovered progenitors for each sample ranges from 0 to 3 (Additional file S1: Table S6). Aggregating the results from the 6 samples, we see that the ratio of missing to known progenitors is 9:70, giving an estimated rate of $13\%$ missing progenitor labels, which is close to the 10% (3/30) fraction of early silencing of target sites observed in most samples.

Leveraging the cell type annotations published in the TLS dataset, we evaluate how the LAML topology recapitulates what is known of the mouse embryogenesis system. Although cell type and lineage are not necessarily expected to conform [4], we still expect cell type transitions to be parsimonious [26]. To quantify concordance with the published cell type development tree, we define the developmental cost to be the number of cell type transitions in the minimum cost labeling $\hat{\ell }$ of the lineage tree T with cell types (see Methods: Developmental cost for a formal definition). The published cell type development tree is consistent with expression analysis performed in [26] and is compatible with the cell type development tree determined from an in vivo mouse model [47].

On the large TLS sample AM-DNA-097, we observe that the LAML topology minimizes the number of cell type transitions compared to the topologies estimated by other methods. This sample has 1836 cells in total, but only 959 cells have unique lineage barcodes. We have annotations for 17 cell types, which can be broadly grouped into 5 widely accepted differentiated cell types (PCGLC, endothelial, endoderm, neural tube, and somite) [26]. We analyze the tree topologies using both resolution levels of the developmental tree, representing two levels of confidence in the cell state annotations: higher resolution with 17 cell types and lower resolution with 5 cell types. The LAML tree has substantially lower developmental cost (LAML: 881 vs. Cassiopeia-Hybrid: 1112 on the lower resolution developmental tree and LAML: 2808 vs. Cassiopeia-Hybrid: 3240 on the higher resolution developmental tree). LAML also provides a similar but smaller improvement on sample 12 which has only 363 cells (LAML: 537, Cassiopeia-ILP: 549).

### Mouse embryo cellular development using intMEMOIR

We compare LAML, TiDeTree, Startle-NNI, and Cassiopeia on a dataset which uses the intMEMOIR dynamic lineage tracing technology [20] to track development in the mouse embryo. The dataset has ground truth lineage trees obtained from microscopic observation of the cell division process. However, this dataset has only 10 sites and 2 mutated states, and thus presents a very challenging dataset for benchmarking lineage tree reconstruction methods, as demonstrated in the DREAM challenge in 2021 [15] where all tested methods obtained a high RF error of 0.55 or more.

LAML achieves comparable RF to state-of-the-art methods benchmarked in DREAM challenge 2021 and has the lowest average RF error (RF = 0.53) among the methods that we tested, followed by TiDeTree (RF = 0.56), Startle-NNI (RF = 0.58), and Cassiopeia (RF = 0.63) (Additional file S1: Fig. S15). Interestingly, although the tree topologies inferred by the different methods have similar RF error, they have large average RF distance to the LAML tree (Startle-NNI: 0.31, Cassiopeia: 0.46, TiDeTree: 0.46). Importantly, compared to TiDeTree (i.e., the second best method), LAML is much faster (Additional file S1: Fig. S16): on average, LAML finished in just under a minute while TiDeTree took $\approx 9$ h to infer the lineage tree for each intMEMOIR sample.

## Discussion

Single-cell lineage tracing technologies continue to advance in scale and resolution, increasing the need for accurate methods to infer lineage trees from the resulting data. We introduce LAML, a maximum likelihood method that jointly estimates the single-cell chronogram, editing rate, heritable missing rate, and dropout probability under the newly developed PMM model for dynamic lineage tracing. LAML can be run (i) to estimate time-scaled branch lengths and other parameters on a given tree topology using the EM algorithm, or (ii) to perform topology search to also estimate the tree topology.

We show on both simulated and real biological data that LAML is more accurate for topology estimation than many existing methods, including a greedy approach (Cassiopeia-greedy), parsimony approaches (Cassiopeia-ILP and Startle-NNI), and Neighbor-Joining (with multiple distance matrices, including DCLEAR). Importantly, on the KP-tracer dataset of mouse lung adenocarcinoma [23], we show that the chronogram inferred by LAML reveals 3 epochs of metastasis progression through time: the primary growth epoch spanned the first 1 month, during which there were no metastasis events; the metastasis epoch spanned from month 1 to 3, during which there was a metastasis burst at month 2; and the late metastasis epoch started at around month 3 and showed a stable proportion of lineages undergoing metastasis and primary reseeding at every time point.

In addition to studying metastasis progression as we demonstrated, the chronogram inferred by LAML also finds applications in other places, such as trajectory inference, subclonal dynamics, and cell migration. For instance, the PMM model has been used as a component of a phylogeographic model to infer spatial cell lineage trees from spatial lineage tracing data [48]. Besides that, the time-scaled tree is helpful in reconstruction of unobserved intermediate cell states [23, 49]. Additionally, as LAML can distinguish heritable missing from dropout, it yields insights for designing new lineage tracing technologies where one controls over the ratio of dropout to heritable missing types for optimal phylogenetic signal. There are also multiple directions to improve LAML. First, although we focus on CRISPR-based lineage tracing systems in this paper, LAML can be applied to any dynamic lineage tracing systems for which the key assumptions in the PMM model hold. If there are an ample number of target sites, several assumptions of the current PMM model could be relaxed, such as the independence of the sites, constant editing rate, and constant heritable missing rate. Additional properties defining prime editors may also be useful to model. Second, runtime and scalability can be further improved by parallelization [50], divide-and-conquer techniques [51, 52], or by approximating the likelihood and quantizing time [53]. Additional scalability improvements could also solve related problems such as phylogenetic placement of newly sampled cells into an existing cell lineage tree [54]. Third, recent simulators such as TedSim [55], which simulate both lineage trees and associated gene expression data, offer promising avenues for further benchmarking. Additional improvements in TedSim or other simulators—including variable branch lengths and fine-grained control over the missing data generation process—would be helpful in benchmarking LAML and other tree reconstruction algorithms. Finally, methods such as LinTIMaT [33] and LinRace [56] integrate lineage barcode and gene expression information to build cell lineage trees. One could extend these ideas to the time-resolved trees generated by LAML; e.g., applying LinRace to refine polytomies on the LAML tree, or extending LAML to use gene expression during tree reconstruction. Finally, LAML could be extended to incorporate additional data, such as time points from multiple rounds of editing (present in technologies such as CARLIN, iTracer, scGESTALT) or the fluorescence tagging in the lineage tracing vector [18, 23], to better infer time-scaled branch lengths.

## Conclusions

LAML estimates a maximum likelihood cell lineage tree from dynamic lineage tracing data under our proposed PMM model. Maximum likelihood approaches have the advantage of directly modeling branch lengths, allowing for accurate, unbiased estimates of both mutation counts and experimental time between ancestral cell divisions. The PMM model is a step towards more realistic models of dynamic lineage tracing and can be applied to other dynamic lineage tracing systems with both heritable and non-heritable missing data. By distinguishing heritable and non-heritable missing data, LAML obtains additional signal to improve phylogeny inference. We demonstrated LAML’s scalability and accuracy in inferring both the topology and branch lengths of lineage trees using modern lineage tracing datasets consisting of thousands of cells.

## Methods

Given a dynamic lineage tracing experiment with *K* target sites^1^ and *N* sequenced cells, we define the $N\times K$ *observed character matrix* ${\textbf {D}}$ to describe the data obtained from experiment. Entries in column *k* of ${\textbf {D}}$ take values in $\mathscr {A}^{(k)}=\{?,0,1,...,M^{(k)}\}$, where $\mathscr {A}^{(k)}$ is the *alphabet* of target site *k*, 0 is the *unmutated state*, $-1$ is the *heritable silent state (before sequencing)*, “?” is the *missing data state (after sequencing)*, and $1,...,M^{(k)}$ are *mutated states*.

We represent the cell lineages by a rooted tree topology *T*. Each leaf node of *T* represents a sampled cell which corresponds to one row of ${\textbf {D}}$ and the internal nodes represent unobserved ancestral cells. Let $\mathscr {L}_T\ \mathscr {V}_T$, and $\mathscr {E}_T$ be the *set of leaves*, *set of nodes*, and *set of edges* of *T*, respectively. Let $r_T$ be the root of *T*. We assume that $r_T$ has exactly one child (the progenitor cell needs time to divide) and all other internal nodes of *T* have exactly two children. Let (*u*, *v*) be the edge^2^ in $\mathscr {E}_T$ from *u* to its child *v* (where $u,v\in \mathscr {V}_T$). Each branch (*u*, *v*) of *T* has an associated branch length that shows the distance between *u* and *v* in time unit.

Given the character matrix ${\textbf {D}}$ and total experiment time $\tau$, our goal is to construct a cell lineage tree topology $\hat{T}$, and a branch length for each edge in $\hat{T}$ that maximizes the likelihood of observing ${\textbf {D}}$. First, we describe the Probabilistic Mixed-type Missing (PMM) model for the likelihood $\mathcal {P}(\textbf{D} | T, \varvec{\Theta })$ where $\varvec{\Theta }$ includes the branch lengths and other parameters. Next, we develop an algorithm to find $\hat{T}$ and $\hat{\Theta }$ that maximizes this likelihood. Note that *T* refers to a given, fixed tree topology, and $\hat{T}$ refers to the maximum likelihood estimator of the tree topology.

### The Probabilistic Mixed-type Missing (PMM) model

The Probabilistic Mixed-type Missing (PMM) model consists of two layers: (1) genome editing at the target sites and (2) single-cell sequencing. Below we describe these two layers in more detail.

#### Layer 1: stochastic model of the induced mutations and heritable missing data

Layer 1 describes genome editing at the target sites. At the beginning of the experiment, we assume there is exactly one *progenitor cell* that has all *K* target sites in the *unmutated state*. (In Multi-progenitor PMM model and inference we generalize the PMM to multiple progenitor cells). During the genome editing process, each target site *k* either mutates into one of the *mutated states* in $\{1,...,M^{(k)}\}$ or into the *silent state* “−1.” A cell that has taken on the silent state “−1” is unable to take on any additional edits. We assume each site *k* mutates following a continuous time Markov chain (CTMC) with the following transition rate matrix ${\textbf {Q}}^{(k)}$:3

Here $q^{(k)}_1,q^{(k)}_2,...,q^{(k)}_{M^{(k)}}$ are the rates of transitioning from 0 to the mutated states of site *k* with $\sum \nolimits _{m=1}^{M^{(k)}}q^{(k)}_m=1$. $\nu$ is the heritable missing rate, which is shared across all sites.

Let $\lambda$ be the editing rate (i.e., rate of changing from 0 to a mutated state) and $t_e$ be the length of a branch $e \in \mathscr {E}_T$ in time units. We assume $\lambda$ is shared across all sites and branches and the branch length $t_e$ for each branch *e* is also shared across all sites. On an edge $e \in \mathscr {E}_T$ at site *k*, the transition probabilities of every pair of states are determined by the *transition probability matrix*, $\varvec{\Psi }^{(k)}_e=\textrm{e}^{{\textbf {Q}}^{(k)}\lambda t_e}$. Performing matrix exponentiation gives the following:4where $\delta _e=\lambda t_e$, which is the length of *e* in mutation unit. Note that given the editing rate $\lambda$, the branch length $t_e$ and value $\delta _e$ can be trivially computed from one another. The matrix $\varvec{\Psi }^{(k)}_e$ determines transition probabilities of every pair of states on *e*. In other words, let $\mathbb {X}$ be the *random* matrix of size $|\mathscr {V}_T|\times K$ which represents the state of the corresponding cell at target site *k* *before single-cell sequencing*; that is, entries in every column *k* of $\mathbb {X}$ take values in $\mathscr {A}^{(k)} \setminus \{?\}$. Then for all $\alpha _u,\alpha _v \in \mathscr {A}^{(k)} \setminus \{?\}$, we have:

5 $$\begin{aligned} \mathcal {P}(\mathbb {X}^{(k)}{(v)}=\alpha _v|\mathbb {X}^{(k)}{(u)}=\alpha _u) = \varvec{\Psi }_e^{(k)}(\alpha _u,\alpha _v), \end{aligned}$$

where $\mathbb {X}^{(k)}(u)$ and $\mathbb {X}^{(k)}(v)$ denote the entries of $\mathbb {X}$ corresponding to site *k* and cells *u* and *v*, respectively.

**Fig. 6:**
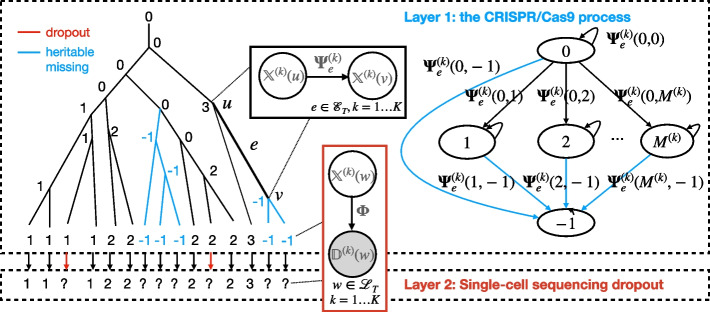
The PMM model with its two layers. Layer 1 (Top): A CTMC that models the process of genome editing and heritable missing and (2) stochastic dropout in single-cell sequencing. 0 denotes the unmutated state, $-1$ denotes the silent state, and other integers denote mutated states. State transitions are demonstrated by both a plate diagram and a transition graph. Transition probabilities are parameterized by $\varvec{\Psi }$ (see Eq. 4). Layer 2 (Bottom): A model for stochastic dropout in single-cell sequencing. The model is parameterized by $\varvec{\Phi }$ as indicated by the plate diagram (see Eq. 6). Observed missing data is represented by “?” and is a mixture of heritable missing (part of layer 1) and dropout (part of layer 2). The two types of missing data are indistinguishable in the observed data

The model is illustrated in Fig. 6. Note that while we assume that $t_e, \delta _e$, and $\nu$ are shared for all sites, we allow each site *k* to have a distinct alphabet $\mathscr {A}^{(k)}$ and a distinct set $\{q^{(k)}_1,q^{(k)}_2,...,q^{(k)}_{M^{(k)}}\}$. To avoid overparameterization, we treat all $q^{(k)}_1,q^{(k)}_2,...,q^{(k)}_{M^{(k)}}$ as hyperparameters (also referred to as *indel priors* in previous works [27, 30]). The user can specify these hyperparameters to be identical (as in [32]), or use external data to provide pre-estimated values (as in [27]). Notably, the transition matrix $\varvec{\Psi }^{(k)}_e$ is sparse and has a special structure: non-zero entries are only in the first row, last column, and main diagonal.

#### Layer 2: model of dropout during single-cell sequencing

The second layer of the model describes the stochastic generation of dropout during single-cell sequencing. After single-cell sequencing, characters in the silent state (i.e., $-1$) will be observed in the missing state (i.e., “?”) with probability 1, while characters with any other (non-silent) states can also be observed in the missing state due to insufficient sequencing information (described by dropout probability $\phi$). We use $\phi$ to represent the *dropout probability* and represent the transition probabilities during single-cell sequencing at site *k* by the following matrix:6

Let $\mathbb {D}$ be the *random* matrix of size $|\mathscr {L}_T|\times K$ whose entries take values in $\mathscr {A}^{(k)} \setminus \{-1\}$. Each entry in column *k* of $\mathbb {D}$ represents the state of the corresponding cell at target site *k* *after single-cell sequencing*. Note that ${\textbf {D}}$, the character matrix, is a realization (observed value) of $\mathbb {D}$. For all $\alpha \in \mathscr {A}^{(k)} \setminus \{?\}$, $\beta \in \mathscr {A}^{(k)} \setminus \{-1\}$ and $w\in \mathscr {L}_T$, we have:

7 $$\begin{aligned} \mathcal {P}(\mathbb {D}^{(k)}{(w)}=\beta |\mathbb {X}^{(k)}{(w)}=\alpha ) = \varvec{\Phi }^{(k)}(\alpha ,\beta ), \end{aligned}$$

where $\mathbb {X}^{(k)}(w)$ and $\mathbb {D}^{(k)}(w)$ denote the entries of $\mathbb {X}$ and $\mathbb {D}$ corresponding to site *k* and cells *w*, respectively.

In summary, the PMM model has the following parameters: branch lengths $t_e$ for each edge *e* in *T*, the editing rate $\lambda$, the silencing rate $\nu$, and the dropout probability $\phi$. In the rest of this paper, we use $\varvec{\Theta }=(\{\delta _e\},\lambda ,\nu ,\phi )$ to denote these parameters. The model is illustrated in Fig. 6.

### Maximum likelihood estimation using LAML

In this section, we describe our Lineage Analysis via Maximum Likelihood (LAML) algorithm for maximum likelihood estimation under the PMM model.

#### Likelihood computation

We assume that sites mutate independently, and thus given tree topology *T* and $\varvec{\Theta }$ describing branch lengths and missing data parameters, the log-likelihood $\log L(T,\varvec{\Theta };{\textbf {D}})$ of the observed data ${\textbf {D}}$ under the PMM model is the sum of the log-likelihoods of the individual sites:

8 $$\begin{aligned} \log L(T,\varvec{\Theta };{\textbf {D}}) = \sum \limits _{k=1}^{K}\log \mathcal {P}({\textbf {D}}^{(k)};T,\varvec{\Theta }). \end{aligned}$$

The probability $\mathcal {P}({\textbf {D}}^{(k)};T,\varvec{\Theta })$ of one site *k* is computed by marginalizing over all possible realizations of $\mathbb {X}^{(k)}$:

9 $$\begin{aligned} \mathcal {P}({\textbf {D}}^{(k)};T,\varvec{\Theta }) & = \sum \limits _{{\textbf {x}}} \mathcal {P}(\mathbb {D}^{(k)}{=} {\textbf {D}}^{(k)}, \mathbb {X}^{(k)}{=} {\textbf {x}}; T,\varvec{\Theta })\nonumber \\ & = \sum \limits _{{\textbf {x}}}\prod \limits _{e=(u,v)\in \mathscr {E}_T}\!\varvec{\Psi }_e^{(k)}({\textbf {x}}(u),{\textbf {x}}(v))\prod \limits _{w\in \mathscr {L}_{\mathscr {T}}}\varvec{\Phi }({\textbf {x}}(w),{\textbf {D}}^{(k)}(w)), \end{aligned}$$

where $\varvec{\Psi }$ and $\varvec{\Phi }$ are defined in Eqs. 4 and 6. While the sum in the above equation is over an exponential number of possible realizations of $\mathbb {X}^{(k)}$, the log-likelihood can be computed in linear time, using Felsenstein’s pruning algorithm [57]. See S2 for a review of the pruning algorithm to compute the likelihood under the PMM model.

#### Maximum likelihood inference

Our goal is to find the tree topology $\hat{T}$ and parameters $\hat{\varvec{\Theta }}$ that maximize the log-likelihood function:

10 $$\begin{aligned} \underset{T,\varvec{\Theta }}{\max}\log {L(T,\varvec{\Theta };{\textbf {D}})}, \end{aligned}$$

such that

11 $$\begin{aligned} \sum \limits _{e \in \textrm{Path}(r_T,w)}\delta _e = \lambda \tau ,\ \text {for all}\ w \in \mathscr {L}_T, \end{aligned}$$

where $\textrm{Path}(r_T,w)$ denotes the path (a list of edges) from the root $r_T$ to a leaf node *w*, and $\tau$ is length of the experiment (must be given by the user). Note that while $\varvec{\Theta }$ does not include $\{t_e\}$, we can compute every $t_e$ from $\lambda$ and $\delta _e$ using $\delta _e=\lambda t_e$. As such, we infer *branch lengths in both mutation units and time units*.

In general, maximum likelihood inference of phylogenetic trees is NP-hard [40]. Therefore, we use the following algorithm to find the maximum likelihood tree and $\varvec{\Theta }$, iterating over two subroutines: (i) for a fixed tree topology *T*, find $\hat{\varvec{\Theta }}$ to maximize $L(\varvec{\Theta };T,{\textbf {D}})$, and (ii) search for the optimal tree topology $\hat{T}$. To solve (i), we devise *an efficient EM algorithm* and to solve (ii) we use simulated annealing with Nearest-Neighbor-Interchange (NNI) operations. Details of both steps are provided below.

#### Estimation of $\hat{\Theta }$ on a fixed tree topology

Given a tree topology *T* the maximum likelihood estimate $\varvec{\hat{\Theta }}$ is defined as

12 $$\begin{aligned} \hat{\varvec{\Theta }} = \underset{\varvec{\Theta } \text { satisfies Eq.~12}}{\text {argmax}}\, \log L(\varvec{\Theta };T,{\textbf {D}}). \end{aligned}$$

We compute $\hat{\varvec{\Theta }}$ using the expectation maximization (EM) algorithm. Since *T* is fixed, we omit *T* from the equations below for brevity.

#### The EM algorithm

The expectation maximization (EM) algorithm has been used to compute maximum likelihood estimates of branch lengths given a fixed tree topology [58, 59]. This algorithm iteratively updates $\{\hat{\delta }_e\}$ by alternating between an E-step and M-step. Building on these works, we derive an efficient EM algorithm to compute the maximum likelihood estimates for all parameters $\hat{\varvec{\Theta }}=(\{\hat{\delta }_e\},\hat{\lambda },\hat{\nu },\hat{\phi })$, which are the estimated branch lengths $\hat{\delta }_{e}$, editing rate $\hat{\lambda }$, heritable missing rate $\hat{\nu }$, and dropout probability $\hat{\phi }$. This algorithm leverages the special structure of the transition matrix (Eq. 6) to reduce the complexity of the E-step and simplify the optimization problem involved in the M-step.

We briefly describe the E-step and the M-step below with the full proof and pseudocode in Additional file S2.

##### E-step

At iteration $t+1$, in the E-step we compute the expected values of the transitions $\mathbb {X}(u)\rightarrow \mathbb {X}(v)$ for every edge $e=(u,v)$ of *T* and expected values of the transitions $\mathbb {X}(w)\rightarrow \mathbb {D}(w)$ for every leaf *w* with respect to the estimate $\hat{\varvec{\Theta }}^t$ of $\varvec{\Theta }$ at iteration *t*. Because both $\varvec{\Psi }_e$ and $\varvec{\Phi }$ are sparse and have special structures, we group the transition types of $\mathbb {X}(u)\rightarrow \mathbb {X}(v)$ into the following 5 groups: (i) 0 to 0 ($z\rightarrow z$), (ii) 0 to any mutated state ($z\rightarrow a$), (iii) 0 to the silent state −1 ($z\rightarrow m$), (iv) any mutated state to another mutated state $a\rightarrow a$, and (v) any mutated state to silent state −1 ($a\rightarrow m$). Similarly, we group the transition types of $\mathbb {X}(w)\rightarrow \mathbb {D}(w)$ into the following two groups: no-dropout, $\mathcal {B}$, and dropout, $\tilde{\mathcal {B}}$. The expected values of these transition types are computed as follows:

13 $$\begin{aligned} \mathcal {C}_e^{z\rightarrow z} & = \sum \limits _{k=1}^{K}\mathcal {P}(\mathbb {X}^{(k)}(u)=0,\mathbb {X}^{(k)}(v)=0|\mathbb {D}^{(k)}={\textbf {D}}^{(k)};\hat{\varvec{\Theta }}^t),\nonumber \\ \mathcal {C}_e^{z\rightarrow a} & = \sum \limits _{k=1}^{K}\sum \limits _{\alpha \in \mathscr {A}^{(k)}\setminus \{0,-1,?\}}\mathcal {P}(\mathbb {X}^{(k)}(u)=0,\mathbb {X}^{(k)}(v)=\alpha |\mathbb {D}^{(k)}={\textbf {D}}^{(k)};\hat{\varvec{\Theta }}^t),\nonumber \\ \mathcal {C}_e^{z\rightarrow m} & = \sum \limits _{k=1}^{K}\mathcal {P}(\mathbb {X}^{(k)}(u)=0,\mathbb {X}^{(k)}(v)=-1|\mathbb {D}^{(k)}={\textbf {D}}^{(k)};\varvec{\Theta }^t),\nonumber \\ \mathcal {C}_e^{a\rightarrow a} & = \sum \limits _{k=1}^{K}\sum \limits _{\alpha \in \mathscr {A}^{(k)}\setminus \{0,-1,?\}}\mathcal {P}(\mathbb {X}^{(k)}(u)=\alpha ,\mathbb {X}^{(k)}(v)=\alpha |\mathbb {D}^{(k)}={\textbf {D}}^{(k)};\hat{\varvec{\Theta }}^t),\nonumber \\ \mathcal {C}_e^{a\rightarrow m} & = \sum \limits _{k=1}^{K}\sum \limits _{\alpha \in \mathscr {A}^{(k)}\setminus \{0,-1,?\}}\mathcal {P}(\mathbb {X}^{(k)}(u)=\alpha ,\mathbb {X}^{(k)}(v)=-1|\mathbb {D}^{(k)}={\textbf {D}}^{(k)};\hat{\varvec{\Theta }}^t),\nonumber \\ \mathcal {B}_u & = |\{k:{\textbf {D}}^{(k)}(u)\ne ?\}| \text { , }\quad \tilde{\mathcal {B}}_u = \sum \limits _{k:{\textbf {D}}^{(k)}(u)= ?}(1-\mathcal {P}(\mathbb {X}^{(k)}(u)=-1|\mathbb {D}^{(k)}={\textbf {D}}^{(k)};\hat{\varvec{\Theta }}^t)). \end{aligned}$$

It is known that all the posterior probabilities $\mathcal {P}(\mathbb {X}^{(k)}(u)=\alpha _u,\mathbb {X}^{(k)}(v)=\alpha _v|\mathbb {D}^{(k)}={\textbf {D}}^{(k)};\hat{\varvec{\Theta }^t})$ can be computed in $\mathcal {O}(M^2NK)$ [58, 59] for all combinations of $e=(u,v),\alpha _u,\alpha _v$, where *M* is the maximum alphabet size, *N* is the number of sequenced cells, and *K* is the number of target sites. Therefore, the complexity of the E-step is also $\mathcal {O}(M^2NK)$. While this complexity is acceptable for most current lineage tracing data, we further reduce the complexity to $\mathcal {O}(NK)$ by leveraging special properties of our model to compute the expected values without knowing all the posterior probabilities (details in Additional file S2).

##### M-step

The M-step of iteration $t+1$ is the following optimization problem:

14 $$\begin{aligned} \hat{\varvec{\Theta }}^{t+1} = \underset{(\{\delta _e\},\lambda ,\nu ,\phi )\ \text {satisfies Eq. 11}}{\text {argmax}}\, \sum \limits _{e\in \mathscr {E}_T}\left( -\mathcal {C}_e^{z\rightarrow z}(1+\nu )\delta _e + \mathcal {C}_e^{z\rightarrow a}(\log (1-e^{-\delta _e})-\nu \delta _e) + \right. & \nonumber \\ \left. \mathcal {C}_e^{z\rightarrow m}\log {(1-e^{-\delta _e\nu })} - \mathcal {C}_e^{a\rightarrow a}\delta _e\nu + \mathcal {C}_e^{a\rightarrow m}\log {(1-e^{-\delta _e\nu })}\right) + & \nonumber \\ \sum \limits _{u\in \mathscr {L}_T}\left( \mathcal {B}_u\log {(1-\phi )} + \tilde{\mathcal {B}}_u\log {\phi }\right) , & \end{aligned}$$

where the coefficients (of form $\mathcal {C}$ and $\mathcal {B}$) in Eq. 14 depend on $\hat{\varvec{\Theta }}^t$ and were computed in the E-step. While this problem is not convex in general, in a special case where the heritable missing rate $\nu =0$, the problem is convex and if we ignore the constraints, we can derive a closed-form solution by a simple change of variables. When $\nu \ne 0$, the problem is still solved efficiently using block coordinate ascent. We use the CVXPY package [60, 61] with the MOSEK solver [62] to solve these convex optimization problems.

##### Case 1: heritable missing rate $\nu =0$

If $\nu =0$, then $\mathcal {C}^{z\rightarrow m}_e=\mathcal {C}^{a\rightarrow m}_e=0$ for all edges *e*. In addition, for all $u\in \mathscr {L}_T$ we have $\mathcal {P}(\mathbb {X}^{(k)}=-1)=0$, so $\tilde{\mathcal {B}}_u=K-\mathcal {B}_u$. Thus, the M-step described by Eq. 14 reduces to:

$$\begin{aligned} \hat{\varvec{\Theta }}^{t+1} = \underset{(\{\delta _e\},\lambda ,\nu ,\phi )\ \text {satisfies Eq.~12}}{\text {argmax}} \left[ \sum \limits _{e\in \mathscr {E}_T}\left( -\mathcal {C}_e^{z\rightarrow z}(1+\nu )\delta _e + \mathcal {C}_e^{z\rightarrow a}\log (1-e^{-\delta _e}) \right) \right. + & \\ \left. \sum \limits _{u\in \mathscr {L}_T}\left( \mathcal {B}_u\log {(1-\phi )} + (K-\mathcal {B}_u)\log {\phi }\right) \right] & \end{aligned}$$

This optimization problem is concave (the objective function is separable, its second partial derivatives are negative, and the constraints are linear).

##### The closed-form M-step when $\nu =0$ and ignoring constraints

*If we ignore the constraints given in Eq.* 11, we obtain a closed-form solution, as follows. Let $\rho _e=e^{-\delta _e}$. Then, the problem reduces to

$$\begin{aligned} \max _{\{\rho _e\},\phi } \sum \limits _{e\in E_T} \mathcal {C}_e^{z\rightarrow z}\log {\rho _e} + \mathcal {C}_e^{z\rightarrow a}\log {(1-\rho _e)}+ \sum \limits _{u\in \mathscr {L}_T}\mathcal {B}_u\log {(1-\phi )} + (K-\mathcal {B}_u)\log {\phi }. \end{aligned}$$

We solve the above problem by simply finding the roots of the partial derivatives, obtaining the following *closed-form* solution:

$$\begin{aligned} \hat{\rho }_e = \frac{\mathcal {C}_e^{z\rightarrow z}}{\mathcal {C}_e^{z\rightarrow z}+\mathcal {C}_e^{z\rightarrow a}}\ \text {and}\ \hat{\phi } = \frac{\sum \nolimits _{u\in \mathscr {L}_T}(K-\mathcal {B}_u)}{\sum \nolimits _{u\in \mathscr {L}_T}K} = \frac{N_{\textrm{missing}}}{NK} \end{aligned}$$

where $N_{\textrm{missing}}$ is the total number of missing entries “?” in ${\textbf {D}}$. Then, $\hat{\delta }_e=-\log {\hat{\rho }_e}$. Because $\hat{\phi }$ only depends on ${\textbf {D}}$, we compute $\hat{\phi }$ outside the EM algorithm. We note that because the constraints given in Eq. 11 are ignored, this closed-form formula only gives solution for branch lengths in mutation units and does not give a closed form solution for $\lambda$.

##### Case 2: heritable missing rate $\nu \ne 0$

If $\nu \ne 0$, the M-step problem is not always convex. However, the problem is convex separately with respect to $\phi$, all $\delta _e$, and $\nu$. In this case, we use block coordinate ascent to find a local optimum, where we sequentially optimize $\phi$, $\delta$, and $\nu$ (see Additional file S2.6 for a proof of convexity and the block coordinate ascent algorithm). In summary, our EM algorithm is an iterative algorithm where each iteration consists of two steps: (i) **E-step**: compute Eq. 13 and (ii) **M-Step**: solve for $\varvec{\Theta }$ in Eq. 14. We present pseudocode in Algorithm 1.

**Figure:**
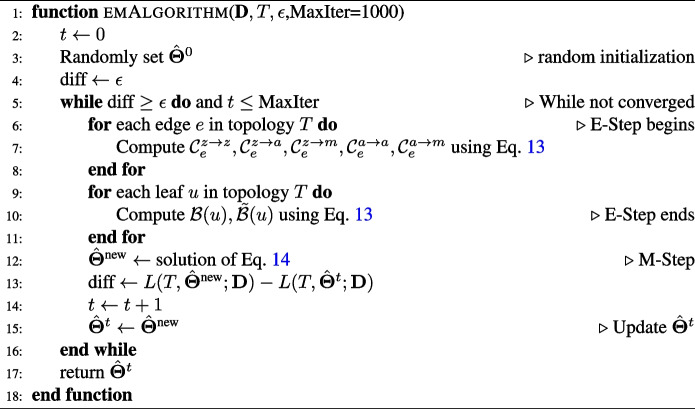
**Algorithm 1** Pseudocode for the EM algorithm. Inputs: **D**: the character matrix, *T*: the tree topology, $\epsilon$: the convergence threshold, and MaxIter (optional): the maximum number of EM iterations allowed (default to 1000). Output: the maximum likelihood estimate $\hat{\varvec{\Theta }}$

### Topology search

We now describe Lineage Analysis via Maximum Likelihood (LAML), an algorithm that alternates between proposing a new tree topology $\hat{T}$ and using the proposed EM algorithm to find the maximum likelihood estimate $\hat{\varvec{\Theta }}$ on this topology. Topology search is performed using nearest neighbor interchange (NNI) with simulated annealing. More specifically, we repeat the following procedure, starting with iteration $t=1$, until convergence or $t>t_\textsf{max}$:

1. At iteration *t*, do the following steps:
  - Propose a new topology $\hat{T}^{(t)}$ using an NNI move on $T^{(t-1)}$.
  - Optimize for $\hat{\varvec{\Theta }}^{(t)}$ using EM algorithm
  - Compute the log-likelihood $l^{(t)} = \log L(T^{(t)},\varvec{\Theta }^{(i)};{\textbf {D}})$
  - Go to (2)
2. If $l^{(t)}>l^{(t-1)}$, then accept $\hat{T}^{(t)}$ and $\varvec{\Theta }^{(t)}$ and go to (3). Otherwise, compute the temperature $\textsf{Temp}(t)$ and acceptance probability *p*(*t*), as follows:
  $$\begin{aligned} \textsf {Temp}(t) & = \max \left( \epsilon ,\frac{\alpha ^t-\alpha ^c}{1-\alpha ^c}\right) \\ p(t) & = \min \left( 1, \frac{\exp (l^{(t)}-l^{(t-1)}-\epsilon )}{\textsf{Temp}(t)}\right) , \end{aligned}$$
  where $c,\alpha$ and $\epsilon$ are hyperparameters controlling the simulated annealing procedure. By default, we set $\epsilon =1e-12,c=20$, and $\alpha =0.9$. Accept $T^{(t)}$ and $\varvec{\Theta }^{(t)}$ with probability *p*(*t*) and go to (3).
3. If $\hat{T}^{(t)}$ and $\hat{\varvec{\Theta }}^{(t)}$ are accepted in (2), then increase *t* and iterate back to step (1). Otherwise, reject $\hat{T}^{(t)}$ and $\hat{\varvec{\Theta }}^{(t)}$; without increasing *i*, turn back to step (1) and try another NNI move of $\hat{T}^{(t-1)}$. If there is no NNI move left or the likelihood improvement is below a predefined threshold $\eta$, stop the procedure and return $\hat{T}^{(t-1)}$ and $\hat{\varvec{\Theta }}^{(t-1)}$.

The pseudocode for LAML is given in Algorithm 2.

**Figure:**
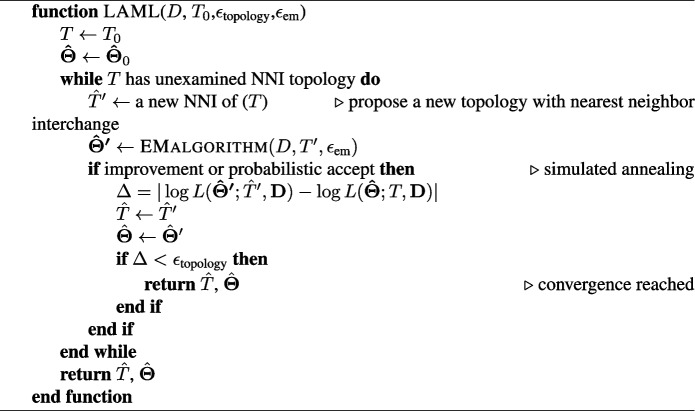
**Algorithm 2** Pseudocode for the LAML algorithm. Inputs: *D*: character matrix, $T_0$: starting tree (either random or constructed using a fast tree construction method), $\epsilon _{\text {topology}}$ and $\epsilon _{\text {em}}$: the thresholds determining convergence of topology search and EM algorithm, respectively. Output: a tree topology *T* and parameters $\hat{\varvec{\Theta }}$

### Multi-progenitor PMM model and inference

Let ${\textbf {s}}$ denote the observed progenitor labels, which is an *N*-dimensional vector corresponding to the progenitor labels of the *N* sequenced cells. Entries in ${\textbf {s}}$ take values in $\{1,2,...,M_s,?\}$, where “?” indicates a missing progenitor label. We assume the missing data in ${\textbf {s}}$ shares the same mechanism as the sites in $\textbf{D}$; that is, ${\textbf {s}}$ shares the same heritable missing rate $\nu$ and dropout probability $\phi$. We also use $-1$ to indicate the silent state. In layer 1, in addition to all components of the (single-progenitor) PMM model, the multi-progenitor extension also has the following CTMC describing heritable missing in ${\textbf {s}}$:15

In layer 2, we add to the PMM model the dropout probabilities of **s**, which shares the same dropout matrix $\varvec{\Phi }$ as all sites in ${\textbf {D}}$. LAML maximizes *the joint likelihood* of ${\textbf {D}}$ and ${\textbf {s}}$ under the multi-progenitor PMM model:

$$\begin{aligned} \hat{T},\hat{\varvec{\Theta }} = \underset{T,\varvec{\Theta }}{\text {argmax}} \log L(T,\varvec{\Theta };{\textbf {D}},{\textbf {s}}) = \underset{T,\varvec{\Theta }}{\text {argmax}}\, \left( \log \mathcal {P}({\textbf {D}};T,\varvec{\Theta })+\log \mathcal {P}({\textbf {s}};T,\varvec{\Theta })\right) \end{aligned}$$

As ${\textbf {s}}$ shares a very similar generative process to every site of ${\textbf {D}}$, the joint likelihood of ${\textbf {D}}$ and ${\textbf {s}}$ on a fixed tree topology can be optimized using the same EM algorithm.

### Simulation procedure

We simulate a model tree of 1024 leaves (i.e., 10 cell generations) using the birth-only model implemented in Cassiopeia. Branch lengths in time units are drawn according to a lognormal distribution with mean 1 and standard deviation 0.1. We simulate $K=30$ target sites under the PMM model, with the values of hyperparameters $q^{(k)}$ at each site *k* selected to match a recent lineage tracing dataset of trunk like structures (TLS) [26]. We also calibrate the simulated data to match two other statistics in the TLS dataset: the expected proportion ($64\%$) of non-zero (mutated) entries in the character matrix and the expected proportion ($25\%$) of missing data. To achieve $64\%$ non-zero entries in the simulated data, we set the editing rate $\lambda =0.095$ and make the molecular clock assumption. We create five model conditions with different proportions of heritable and non-heritable missing data by varying $\phi$ and $\nu$ (Table 1). To model the sampling of sequenced cells, each simulated character matrix is subsampled to 250 cells and the model tree is restricted to the same subset. We repeat the subsampling procedure five times for each character matrix.

### Computing maximum parsimony branch lengths

To compute MP branch lengths, we follow a standard practice of many maximum parsimony methods such as Startle and Cassiopeia: use Sankoff’s algorithm to infer the ancestral sequences and compute the length of each branch (*u*, *v*) as the number of mutations separating nodes *u* and *v* from one another. Note that each MP branch length is an estimate of the *number of mutations* that occurred on the corresponding branch (i.e., mutation count). Therefore, as a best effort to “convert” the MP branch lengths to time units, we divide them by the true editing rate ($\lambda =0.095$; known in the simulation) before evaluating them against the true branch lengths.

### Evaluation metrics

In this section, we describe all the evaluation metrics used for data analysis.

#### The normalized Robinson-Foulds (RF) error

For simulated data and the intMemoir dataset where the ground-truth topology is known, we measure topological error by the *RF error*. The RF distance between two trees $T_1$ and $T_2$ on a same leafset is the sum of false positives (FP—bipartitions in $T_2$ not present in $T_1$) and false negatives (FN—bipartitions in $T_1$ not present in $T_2$). We normalize the RF distance by the total number of internal edges in both trees. Thus:

16 $$\begin{aligned} \Delta _{RF} (T_1,T_2) = \frac{FN + FP}{n_{internal}(T_1) + n_{internal}(T_2)}, \end{aligned}$$

where $n_{internal}$ denotes the number of internal edges of a tree. The RF error of an estimated topology is simply its normalized RF distance to the true topology.

#### Root-mean-square error (RMSE)

We use *root-mean-square error (RMSE)* to compute the error in estimating $\phi$ and $\nu$. Specifically, for each model condition where $\theta ^*=(\phi ^*,\nu ^*$) is the true set of parameters, the error of an estimate $\hat{\theta }=(\hat{\phi },\hat{\nu })$ is $d(\hat{\theta },\theta ^*)=(\hat{\phi }-\phi ^*)^2 + (\hat{\nu }-\nu ^*)^2$. The RMSE of the estimates across *m* replicates is

17 $$\begin{aligned} \text {RMSE}(\{\hat{\theta _i}\},\theta ^*) = \sqrt{\sum \limits _{i=1}^{m} \frac{d(\hat{\theta }_i, \theta ^*)}{m}}, \end{aligned}$$

where $m=50$ in all model conditions of our simulation.

#### Migration cost

For the sample 3724_NT_T1_All from the KP-tracer data [23], we compare the tree inferred by LAML to the trees inferred by other methods using the *migration cost*, which is the minimum of migrations required to obtain the observed anatomical labels of the cells. This metric is the same as that used in [30], and was introduced and implemented in MACHINA [45]. Below we briefly describe the construction of a *migration graph*; see [30] and [45] for more details.

1. First, infer the ancestral labeling using maximum parsimony; i.e., label the internal nodes to obtain the fewest migrations.
2. Collapse all branches where the parent node and child node have identical labels.
3. Group all unique (parent state, child state) edges. Each state tuple should be annotated with the number of times edges labeled with (parent state, child state) were observed in the tree. These correspond to migration events.

The *migration cost* is the sum of all edge weights. The *reseeding cost* is the sum of the edge weights of all edges pointing from a non-primary site to the primary site. See Additional file S1.8.2 for a more detailed treatment of the migration cost metric and its variations.

#### Comparison of phylogenetic distance and gene expression

For the 6 samples from KP-Tracer [23], we evaluate the branch lengths by comparing the phylogenetic distances on a cell lineage tree with the distances between the gene expression at the leaves. For this evaluation, we gave all methods the published tree topologies and only estimate branch lengths. We construct two pairwise *phylogenetic distance matrices*, one using the edge lengths estimated by maximum parsimony (MP, see Computing maximum parsimony branch lengths) and another one using edge lengths estimated by maximum likelihood (ML) using LAML (under the PMM model). In addition, we add another distance matrix computed solely from tree topology (i.e., setting all branch lengths to 1), used as control. We process the gene expression data by performing dimensionality reduction, using PCA (10 PCs), scVI (10 PCs), and UMAP (2 PCs). We use the published vectors provided in KP-tracer for scVI and UMAP, and perform PCA using SCANPY [63]. We measure gene expression distance using PCA, scVI, and UMAP to match the gene expression analysis performed in the original KP-Tracer paper [23] (Additional file S1: Fig. S10B). We compute a phylogenetic distance and matrix for each of the three methods described above, and a *gene expression distance matrix* by calculating Euclidean distance between gene expression vectors following each of the three dimensionality reduction techniques described above. For each pair of phylogenetic and gene expression distance matrices, we compute the mutual information as presented in [64, 65], implemented in scikit-learn [66].

#### Quantifying progenitor discordance using normalized triplet error

We compute the progenitor discordance of a tree topology *T* by the normalized triplet error, a quantity commonly used in phylogenetics [67–70]. We compute this quantity as follows. First, we list all the ways to select a triplet of leaves (*a*, *b*, *c*), corresponding to cells with known progenitor labels, to form a set $\mathcal {R}$. Next, we form a subset $\mathcal {R}_{\text {resolved}}$ of *R* containing the triplets that have two cells with the same progenitor label and one cell with a different progenitor label. For each triplet $(a_1,a_2,b)$ in $\mathcal {R}_{\text {resolved}}$ where $a_1$ and $a_2$ share a progenitor label and *b* has a different progenitor label, we find the triplet induced by *T* on $\{a_1,a_2,b\}$. We say that *T* is concordant with the progenitor labels on $\{a_1,a_2,b\}$ if the induced triplet tree has *b* outgroup to $a_1,a_2$; otherwise, it is a discordant. The normalized triplet error is defined as the number of discordant triplets divided by the size of $\mathcal {R}_{\text {resolved}}$. We used the tqDist [71] library to efficiently compute the normalized triplet error.

#### Developmental cost

For sample 12 from the TLS data [26], we compare the tree inferred by LAML to the trees inferred by other methods using the developmental cost. Given cell development tree $T_D$, a lineage tree *T*, and a leaf labeling $\ell$, the developmental cost is the minimum number of cell type transitions consistent with $T_D$, over all labelings of the lineage *T* which extend the observed leaf labeling $\ell$. Formally, we define the developmental cost $d^*$ as:

18 $$\begin{aligned} d^* & = \min _{\hat{\ell }\ \text {extending}\ \ell } \sum \limits _{e = (u, v) \in E(T)}c(\hat{\ell }(u), \hat{\ell }(v)) \quad \text {where}\nonumber \\ c_{s,t} & = \left\{ \begin{array}{ll} d_{T_D}(s,t) & \text {if node}\ t\ \text {is reachable from node}\ s\ \text {in}\ T_D, \\ \infty & \text {otherwise} \end{array}\right. \end{aligned}$$

To compute the developmental cost, we apply Sankoff’s algorithm [42]. A key caveat of this analysis is that the true mouse developmental tree remains unknown and cell state may not always conform with cell lineage [4].

## Supplementary information

Supplementary Material 1: Supplementary data. Provides data for additional analyses referenced in the paper. Additional topics include an exploration of scalability, extensive benchmarking results, and further exploration of the KP-Tracer, TLScL and intMEMOIR datasets.

Supplementary Material 2: Supplementary methods. Discusses computation of the tree likelihood, a derivation of the EM algorithm, and proofs and algorithms associated with improving the EM complexity.

## Data Availability

The source code for LAML is available on GitHub at https://github.com/raphael-group/LAML [72] and on Zenodo at 10.5281/zenodo.15570612 [73]. Simulation data and evaluation scripts are available on GitHub at https://github.com/raphael-group/laml-experiments [74] and on Zenodo at 10.5281/zenodo.15570712 [75]. Both repositories are published under the Berkeley Software Distribution (BSD) license. The sequencing data used in this study is available on Zenodo (KP-Tracer: 10.5281/zenodo.5847462 [23], TLScL: 10.5281/zenodo.10791343 [26], intMEMOIR: 10.22002/D1.1444 [15]).

## Footnotes

1 Following the convention in phylogenetics, we use *target site* and *site* interchangeably.

2 Throughout this paper, we use *edge* and *branch* interchangeably.
