# Supplementary material for: Maximum likelihood inference of time-scaled cell lineage trees with mixed-type missing data using LAML
Source: Genome Biol. 2025 Jul 2;26:189. doi: 10.1186/s13059-025-03649-9 (PMC12220811; doi:10.1186/s13059-025-03649-9)
Supplement: Supplementary file 1 — Supplementary Material 1: Supplementary data. Provides data for additional analyses referenced in the paper. Additional topics include an exploration of scalability, extensive benchmarking results, and further exploration of the KP-Tracer, TLScL and intMEMOIR datasets. [file 13059_2025_3649_MOESM1_ESM.pdf]

S1: Supplementary data

Maximum Likelihood Inference of Time-scaled Cell Lineage Trees with Mixed-type Missing Data using LAML

Gillian Chu[1,†] Uyen Mai[1,†] Henri Schmidt[1] Benjamin J. Raphael[1,*]

[1]Department of Computer Science, Princeton University, NJ 08540, USA
[†] These authors contributed equally to this work.

[*]Correspondence: braphael@princeton.edu

Appendix S1 Supplementary data

Contents

S1.1 Simulated data . . . 1
S1.1.1 Benchmarking LAML . . . 1
S1.1.2 Leveraging the Statistical Model in Distance-Based Approaches . . . 3
S1.2 Processing Input Data . . . 4
S1.3 The KP-tracer data . . . 4
S1.4 The intMEMOIR data . . . 5
S1.5 The TLScL data . . . 6
S1.6 Supplementary Results for Simulated Data . . . 6
S1.7 Benchmarking LAML . . . 6
S1.7.1 Scalability Results . . . 10
S1.8 Supplementary Results for KP-Tracer . . . 11
S1.8.1 Correlation of allelic distances and phylogenetic distances . . . 11
S1.8.2 Variations on the Migration Cost . . . 13
S1.8.3 Metastasis Analysis . . . 14
S1.9 Supplementary Results for the mouse embryo cellular development using TLS 16
S1.10 Supplementary Results for the mouse embryo cellular development using intMEMOIR . . . 17
S1.11 Distance-Based Exploration . . . 20
S1.12 Review of Lineage Tracing Technologies . . . 20
S1.13 Review of Other Computational Approaches . . . 21

S1.1 Simulated data

We simulate data according to the procedure described in Section 1 of the main paper. In this section we describe how various methods were run on the simulated data.

S1.1.1 Benchmarking LAML

Using this simulated dataset, we benchmark LAML against other methods: Greedy method (based on the principle of perfect phylogeny, used as the baseline), Startle [30], Neighbor Joining [41] (implemented in Cassiopeia, with weighted Hamming distances [27]), and DCLEAR. For the Greedy method, we use the implementation of Cassiopeia [27]). We run Startle in NNI mode with 250 iterations (Startle-NNI) and use the topology estimated by Greedy method as the starting tree. LAML was run using Startle-NNI as the starting tree; simulated annealing NNI with EM optimization was performed until convergence of likelihood.

We used the following commands to run each method. For all Cassiopeia-implemented methods, we used the wrapper script: `cassiopeia_solvers_with_pickle.py`. Following the default tutorial pipeline, we allow Cassiopeia to collapse all mutationless edges, producing trees with polytomies.

1. Startle-NNI: `python startle.py <seed_tree> <character_matrix> -e <mutation_priors> --iterations 250 --output tree.nwk`
2. Hybrid method (Cassiopeia-Hybrid): `cas.solver.HybridSolver(cell_cutoff=100)`

3. ILP method (Cassiopeia-ILP) with default parameters: `cas.solver.ILPSolver()`
4. ILP method (Cassiopeia-ILP) with non-default parameters:

```
cas.solver.ILPSolver(convergence_time_limit=300,
convergence_iteration_limit=5,
    maximum_potential_graph_layer_size=500)
```

5. Greedy method (Cassiopeia-Greedy): `cas.solver.VanillaGreedySolver()`
6. Neighbor Joining (Cassiopeia): `cas.solver.NeighborJoiningSolver( dissimilarity_function= cas.solver.dissimilarity.weighted_hamming_distance, add_root=True)`
7. LAML: `python run_problin.py -t <starting_tree> -c <character_matrix> -p <mutation_prior> -o <output> -v --delimiter comma --nInitials 1 --topology_search --ultrametric > <logfile> 2>&1`

To run the Cassiopeia-Hybrid and Cassiopeia-ILP versions, we ran Cassiopeia Release (tag 2.0.0) https://github.com/YosefLab/Cassiopeia/releases/tag/2.0.0, as well as the development version (last updated Aug 8 2023, commit hash: `f89530145adad929fc47a2e80f70a93c6af2cf3f`). Neither Cassiopeia-ILP (run with default parameters) nor Cassiopeia-Hybrid ran to completion on our larger simulated datasets containing more missing data. Both Cassiopeia-Hybrid and Cassiopeia-ILP failed to provide output on several of the datasets in the allotted time. We provide further details in the simulated data section and note that Cassiopeia-ILP (run with non-default parameters) was added to some of the evaluations in an effort to provide partial benchmarking results.

There are two ways of running DCLEAR. The first, DCLEAR using a k-mer based approach, is referred to as `DCLEAR (KRD)`, and the second, DCLEAR using a training-based approach, is referred to as `DCLEAR (WHD)`. Here we report results for `DCLEAR (KRD)` only, because `DCLEAR (WHD)` produced segmentation fault errors. We followed the tutorial available here: https://ikwak2.github.io/tmphtml/Example_subchallenge2.

To run `DCLEAR (KRD)`, we note that `DCLEAR` assumes characters across different sites share an alphabet. However, when we converted our simulated data to follow this convention, `DCLEAR` could not scale to handle alphabet sizes of approximately 250. Instead, we report results from running `DCLEAR` directly on the input character matrices, which were also directly provided to the other benchmarked methods. We followed this `DCLEAR (KRD)` vignette: https://colab.research.google.com/gist/gongx030/653a76bffc4ee6ff41499e0026b6d39a/krd.ipynb.

Given a fixed topology, we used LAML to estimate numeric parameters (branch lengths, missing data rates). We used the following command to do so:

```
python run_problin.py -t <fixed_topology> -c <character_matrix>
 -p <mutation_priors> -o <output> -v --delimiter comma --nInitials 1
--ultrametric
```

We run TiDeTree (last accessed: Feb 12, 2025) with the recommended executable published on Github. Two limitations of TiDeTree required modification to run on our simulated data. (1) By design, TiDeTree does not offer support for heterogeneous target site alphabets (distinct

edit alphabets for each target site); and (2) the TiDeTree documentation and codebase do not specify how to input missing data (more importantly, how to specify a "silenced" state).

To address the first limitation, we input into TiDeTree the union of all alphabets from all sites, noting that a given state i at target site k is counted as a different state if it appears in target site j, for $j \neq k$. We input the edit frequencies as the prior into TiDeTree, and initialized the silencing rate parameter to the true value. To address the second limitation, we treated the last edit state as the silenced state according to line 11 from the tidetree/src/tidetree/evolution/datatype/EditData.java file (https://github.com/seidels/tidetree/blob/main/src/tidetree/evolution/datatype/EditData.java#L11). Following this convention, we represented the silenced state as the last edited state, but did not pass a frequency or prior edit rate for this value.

S1.1.2 Leveraging the Statistical Model in Distance-Based Approaches

One of our methodological contributions in this paper is a new probabilistic model of CRISPR/-Cas9 lineage tracing. In this section we elaborate on the applications of this new statistical model.

Using the statistical model, we can estimate maximum likelihood distances between any two sequences. To explore how maximum likelihood distances under our statistical model compare to other ways of computing distances, we compute a distance matrix using several different approaches and run the widely-used distance-based method Neighbor Joining (NJ). This comparison is run on simulated data under all model conditions (varying missing data type composition).

We explain each pipeline in greater detail below:

1. wHD (weighted Hamming Distance): Standard Hamming distance is modified by provided weights. These weights are negative log transformed from the prior transition probabilities for each site's alphabet. wHD is recommended within the Cassiopeia implementation of NJ [27, 34].
```
cas.solver.NeighborJoiningSolver(dissimilarity_function=
cas.solver.dissimilarity.weighted_hamming_distance, add_root=True)
```
2. modified-AC (modified Allelic Coupling distance): Standard allelic coupling distance [23] is modified here by consideration of the missing state. This distance matrix is passed to PHYLIP-NJ to construct a tree for each sample. We use the `get_wAC.py` script to calculate the distance matrix.
3. MLpair: Given an input character matrix, we use LAML to estimate maximum likelihood distances for each rooted pair of leaves. After building this distance matrix, we run PHYLIP-NJ to construct a tree for each sample. We use the `split_pairwise.py` script to split the input distance matrix into pairs, and compute the maximum likelihood distances for each rooted pair.
4. MLall: Running LAML in the default way, we estimate maximum likelihood distances over all pairs of leaves at once, exploring tree topology space to find a maximum likelihood tree.

S1.2 Processing Input Data

For the three biological datasets we analyzed (KP-Tracer, TLS and intMEMOIR), we used the character matrices from the respective publications without further modification. Although we don't perform any additional data processing, we broadly summarize the data pipeline below.

We refer the reader to the detailed documentation provided in Cassiopeia to understand the heuristic process of transforming raw sequencing FASTQ reads into character matrices. The KP-Tracer and TLS character matrices used in the paper were constructed using Cassiopeia's end-to-end pipeline to process raw sequencing reads in FASTQ format into character matrices. Many heuristics affect this long process, which are well-documented in the respective publications: error-correcting cell barcodes, collapsing reads into unique molecular identifiers (UMIs), aligning the sequences to the reference target site, resolving a single sequence for each UMI, calling alleles for each cell, etc. Notably, the Cassiopeia pre-processing pipeline assumes that the unedited state can be distinguished from the missing data case, which exclusively describes the absence of any sequencing reads after filtering. The data also come with character matrices for multiple samples, each has its own per-site alphabet hyperparameters $q(k)$.

S1.3 The KP-tracer data

We benchmark LAML on KP-tracer, a recently published dynamic lineage tracing experiment in mouse models of metastatic lung adenocarcinoma [23]. The data come with character matrices for multiple samples, each has its own per-site alphabet hyperparameters $\mathbf{q}^{(k)}$. These character matrices were obtained directly from the KP-Tracer publication (https://zenodo.org/records/5847462). As is detailed in that publication, the allele tables are processed into character matrices using the Cassiopeia data processing pipeline. Note that for a given target site, the absence of reads is recorded as missing data. For additional details on construction, we refer the reader to the original publication.

To evaluate tree topology, we use the largest tumor dataset, `3724_NT_T1_All`, which consists of $21,108$ cells and 9 target sites. Originally, Yang et al. published a phylogeny using the Cassiopeia-Hybrid (Cass-hybrid) method [23]. Later, Sashittal et al. [30] published another phylogeny using Startle-NNI that yielded a more parsimonious explanation of the metastatic process compared to the published Cass-hybrid phylogeny. We use the migration cost metric, which we discuss below Supplementary Section 1. To run LAML, we first remove cells with identical sequences from the original character matrix, resulting in $1,461$ unique sequences. Then we prune the published Startle-NNI tree to this set of $1,461$ sequences and use it as starting tree. We search for the tree that optimizes the log-likelihood of the sequence data using the provided hyperparameters. We run topology search in LAML for 450 iterations to optimize for log-likelihood, which is improved from -12288 in the Startle-NNI tree to -12208 in the LAML tree. Finally, we add the duplicated sequences to the tree as polytomies.

To evaluate branch length estimation, we use 6 other samples of KP-Tracer that have a medium-high number of target sites (29 to 73) and moderate number cells (29 to 294); these are: 3432_NT_T1, 3435_NT_T4, 3520_NT_T1, 3703_NT_T1, 3703_NT_T2, and 3703_NT_T3. For these 6 selected samples, we use the published tree topologies (estimated by Cassiopeia) and estimate branch lengths using LAML (here after referred to as dML), and compare our estimate to that of maximum parsimony (here after referred to as dMP).

S1.4 The intMEMOIR data

The MEMOIR [16] technology encodes DNA barcodes, and performs lineage tracing by accumulating variable edits in the encoded synthetic target array, and which allows readout by imaging. The intMEMOIR technology [20] permits extended recording and germline transmission, so that the true evolutionary history of sampled cells is recorded. In the intMEMOIR technology, only two edits are possible (either deleting or inverting the target region), so that only three states are possible at any of the sites. We should note this is in sharp contrast to the technology used in [4], where each site can have a unique, variably-sized alphabet of possible edits. In this dataset, we have 106 samples, each with 10 sites, with a maximum of 40 cells and a minimum of 4 cells. The intMEMOIR dataset was used in the original DREAM challenge [15], making it a widely used benchmarking dataset. Details on how the sequences were processed can be found in the original DREAM challenge publication [15]. We used Cassiopeia's processed character matrices [15]. TiDeTree [32], which takes a Bayesian approach to inferring phylogenies, and was intended for use as a module in the BEAST package, was also tested on the intMEMOIR dataset.

The TiDeTree [32] paper details that the topologies for all 106 intMEMOIR samples were jointly estimated, with shared scarring rates and population dynamic parameters across all trees, and assuming a molecular clock. They benchmark against AMbeRland, Guna lab, Cassiopeia, Jasper06, pRennert and RnlLabs. The author kindly made their resulting maximum clade credibility (MCC) trees available to us. We post-processed these trees as TiDeTree's supplement specified, by running BEAST's TreeAnnotator tool (with the "maxium clade credibility" option to create a summary tree.

We benchmarked LAML against Startle-NNI, Cassiopeia and TiDeTree using the following commands:

1. Startle-NNI:
```
python startle.py $seed_tree $character_matrix -e $mut_priors
--iterations ${iters} --output
${output_dir}/startlenni_tree_collapsed.newick
```
2. Cassiopeia: We ran Cassiopeia-Hybrid with a cut-off of 100 cells, so that we effectively report results for Cassiopeia-ILP on all intMEMOIR samples. Note that Cassiopeia-ILP is set up with non-default parameters in order to ensure a resulting tree could be produced in the allotted time-frame and compute.
```
cas.solver.HybridSolver(cas.solver.VanillaGreedySolver(), cas.
    solver.
ILPSolver(convergence_time_limit=100,
    convergence_iteration_limit=1,
maximum_potential_graph_layer_size=1000), cell_cutoff=100)}
```
3. LAML:
```
python /n/fs/ragr-research/projects/problin/run_problin.py -t $tree
-c $msa -p $prior -o $prefix.txt -v --delimiter comma --nInitials 1
--topology_search --ultrametric --maxIters 2500 --parallel >
$prefix.log 2>&1
```
4. TiDeTree:
We adapted the provided example scripts https://github.com/seidels/tidetree/blob/main/

examples/, using the default parameters and provided the sequence information. We installed BEAST 2.7 and used the following recommended compiled jar file on the adapted example XML file:
`java -jar bin/tidetree.jar examples/adapted_example.xml`

S1.5 The TLScL data

The TLScL [26] dataset is a recently published dynamic lineage tracing dataset on mouse embryonic trunk-link structures. We obtained character matrices directly from the TLS publication. As is detailed in the original paper and repository [26], the allele tables are processed into character matrices using the Cassiopeia data processing pipeline. Note that for a given target site, the absence of reads is recorded as missing data. The matrices we used to compare were obtained from the original publication; for details on construction, we refer the reader to the original publication.

S1.6 Supplementary Results for Simulated Data

	**Greedy method**	**Startle-NNI**	**LAML**
RF distance to the true tree	0%	0%	**100%**
Weighted parsimony cost	0%	**84%**	16%
Log-likelihood	0%	0%	**100%**

Table S1 Robinson-Foulds error (RF), weighted maximum parsimony (WMP), and log likelihood (LLH) of the methods on simulated data, summarized over all five model conditions (250 replicates). For each pair of method and metric, the percentage of times the method scored the best according to that metric is shown.

S1.7 Benchmarking LAML

We designed a second set of experiments specifically to benchmark LAML, Startle-NNI, Cassiopeia-Greedy, Cassiopeia-Hybrid, Cassiopeia-ILP and TiDeTree as we vary (1) sequence length, (2) alphabet size, (3) missing data proportion. Broadly, we observe that LAML has the lowest error in the inferred tree topology across all benchmarked methods as the sequence length increases and the alphabet size increases, and the missing data proportion increases (Figures S1 and S2). Note that Cassiopeia-ILP and TiDeTree failed to produce results with the alloted constraints (24 hours and 4GB of memory).

LAML has the lowest topology error as the sequence length increases and the alphabet size increases (Figure S1). LAML consistently performs better than the next best method (Startle-NNI), with greater improvement as the sequence length increases. LAML also has the lowest topology error as the max alphabet size increases.

LAML also has the lowest topology error as the missing data proportion increases (Figure A5). LAML consistently performs better than the next best method (Startle-NNI), with a smaller margin of improvement as the proportion of missing data increases from 25% to 75%.

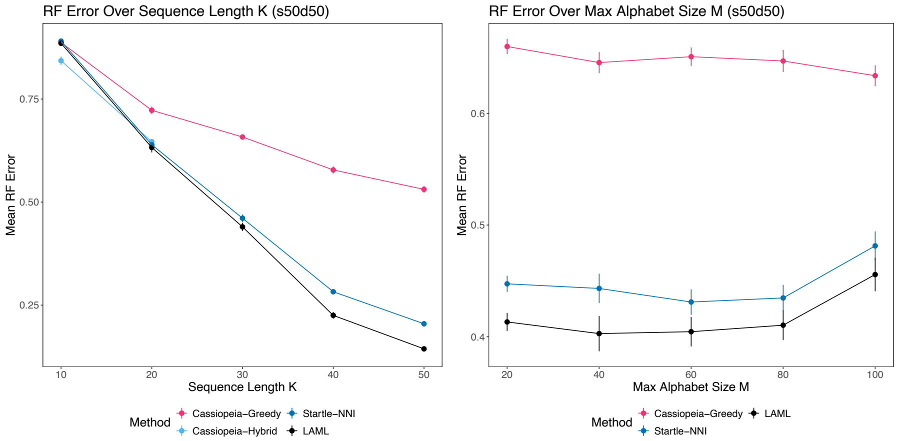

Fig. S1 Comparison of topology error (Robinson-Foulds) for LAML, Startle-NNI, Cassiopeia-Greedy, Cassiopeia-Hybrid, Cassiopeia-ILP and TiDeTree on simulated topology error (Robinson-Foulds) on simulated data with 250 cells and 25% missing data. Note that all methods were given 24 hours and 4 GB of memory to run, but Cassiopeia-Hybrid provided only partial results, and TiDeTree and Cassiopeia-ILP did not finish on any of the inputs. (Left) Varying sequence length. (Right) Varying alphabet size. We hypothesize that the increase in topology error at M=100 is due to the increased runtime in Startle-NNI resulting in fewer explored topologies in the allotted time.

We note that when there is no missing data, Cassiopeia-Hybrid, Startle-NNI and LAML all perform similarly. On the 34/250 samples where Cassiopeia-Hybrid completed, it achieved the lowest RF error. However, Cassiopeia-Hybrid is unable to scale in the presence of 25% missing data, where LAML produces solutions with the lowest average RF error. As the missing data proportion increases, the error of all methods increase, but we note that this is to be expected in cases of low lineage tracing data quality (75% missing data). On a smaller simulation dataset of 30 cells with 25% missing data, we observe that LAML produces the lowest RF error.

We hypothesize that TiDeTree is unable to compute the posterior likelihood of the tree due to numerical underflow as the data size increases. To assess this, we further reduced the size of the dataset to 15 cells, and found that TiDeTree does initialize properly on some of the replicates.

In Fig S3 we have simulated data results illustrating that LAML produces reasonable parameter estimates on different tree topologies. LAML produces accurate estimates of heritable silencing rate ν and dropout probability ϕ, acheiving the most accurate parameter estimates when provided with the most accurate tree topology (the most accurate tree topology is given by LAML, shown in gray in Fig S3A, see 2 for topology error). Compared against Cassiopeia-Greedy, Neighbor-Joining, and Startle-NNI, LAML also produces the most accurate estimate of the true number of mutations (Fig S3B), even as the collision probability at each target site increases (Fig S3C).

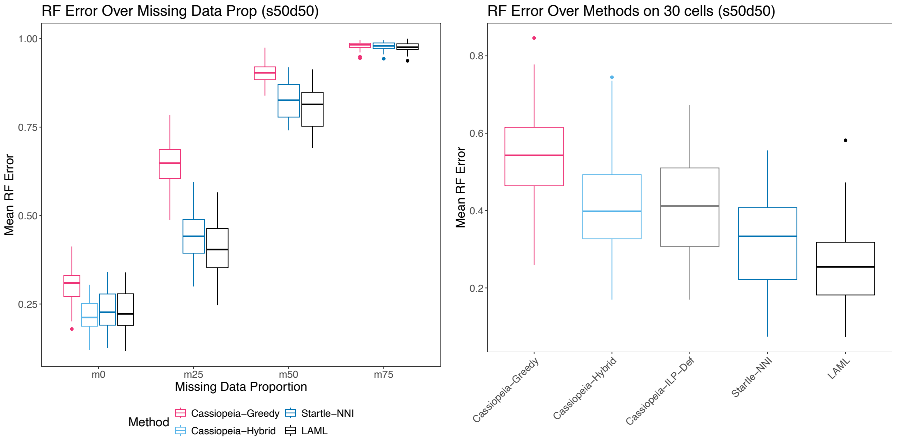

Fig. S2 Comparison of topology error (Robinson-Foulds) for LAML, Startle-NNI, Cassiopeia-Greedy, Cassiopeia-Hybrid, Cassiopeia-ILP and TiDeTree on simulated data. Note that all methods were given 24 hours and 4 GB of memory to run, but TiDeTree did not finish on any of the inputs. (Left) Varying missing data proportion with 250 cells. Startle-NNI was run starting on Cassiopeia-Hybrid where it was available (34/250 jobs), and on Cassiopeia-Greedy where it was not. Results are shown on all samples. Cassiopeia-ILP is also not shown here as it was unable to produce inferred trees. (Right) Topology accuracy on small dataset with 30 cells and 25% missing data. Note that Cassiopeia-ILP-Def indicates Cassiopeia-ILP-Default was run with default settings.

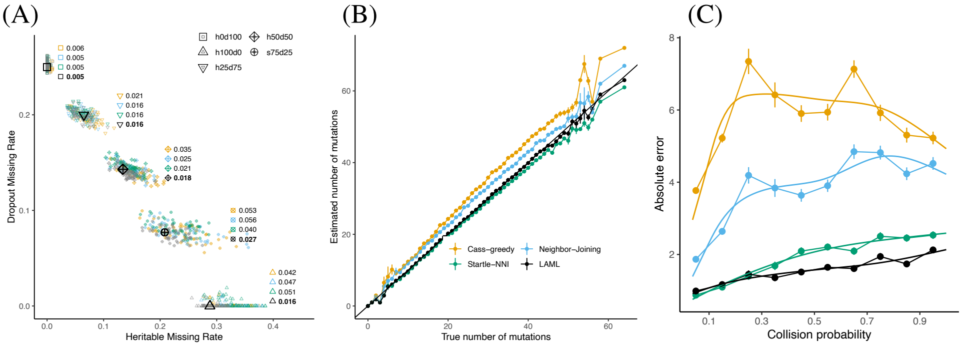

Fig. S3 Supplementary results on the simulated data (see (B) for color code). (A) Estimation of ϕ and ν. True values of each model condition are shown in black, while the estimates using different tree topologies are shown in different colors, together with the root-mean-square-error (RMSE) of the estimates on each model condition. (B) Estimated versus true number of mutations. Each dot is the average estimated value by each method around one true value, shown with error bar. (C) Absolute error in estimating number of mutations versus collision probability of each target site. Collision probability (x-axis) is computed for each target site across all model condition. The x-axis is discretized into 10 bins and absolute error is averaged for each bin, shown with error bar. For (B) and (C), results are combined for all model conditions.

In Figure S4, we have simulated data results illustrating that LAML produces the most likely tree, as well as relatively parsimonious trees. However, we should note that the most parsimonious tree (Startle-NNI) shows very different RF error (2). Thus, likelihood and RF are more correlated than parsimony and RF are, suggesting that likelihood may be a better metric for scoring trees.

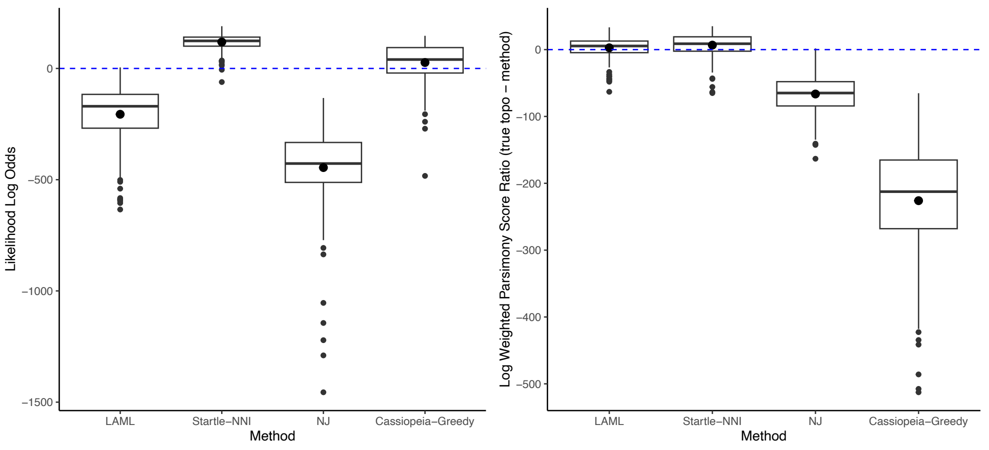

Fig. S4 Comparison of LAML, Startle-NNI, Neighbor-Joining (NJ), and Cassiopeia-Greedy based on two metrics: estimated log likelihood (calculated by LAML) to the true tree likelihood on simulated data (left); and weighted parsimony score (right). This was run on simulated data with 250 cells and 25% missing data, across the five different missing data model conditions.

In Figure S5, we show an additional comparison to Cassiopeia-ILP (run with non-default parameters) on simulated data subsampled to 250 cells, across the five model conditions. Cassiopeia-ILP produced results on 44/250 samples (h0d100: 13, h100d0: 8, h25d75: 15, h50d50: 4, h75d25: 4). For those which Cassiopeia-ILP did not produce results on, 126 of the jobs did not finish running given 168 hours of runtime (7 days), 48 jobs ran out of memory (given 4G per job) and 32 of the jobs fail with an "Index-Error," failing to build the potential graph at all.

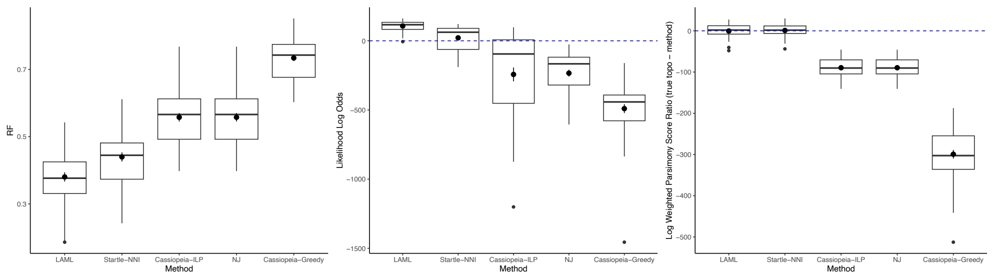

Fig. S5 Comparison of LAML, Startle-NNI, Cassiopeia-ILP, Neighbor-Joining (NJ), and Cassiopeia-Greedy based on two metrics: estimated log likelihood (calculated by LAML) to the true tree likelihood on simulated data (left); and weighted parsimony score (right). This was run on simulated data with 250 cells and 25% missing data, across the five different missing data model conditions. We note that Cassiopeia-ILP's poor parsimony score is likely due to the parameters we modified in order to allow it to run on 250 cells with 25% missing data, and that these results are subset to those $44/250$ samples for which Cassiopeia-ILP produced results.

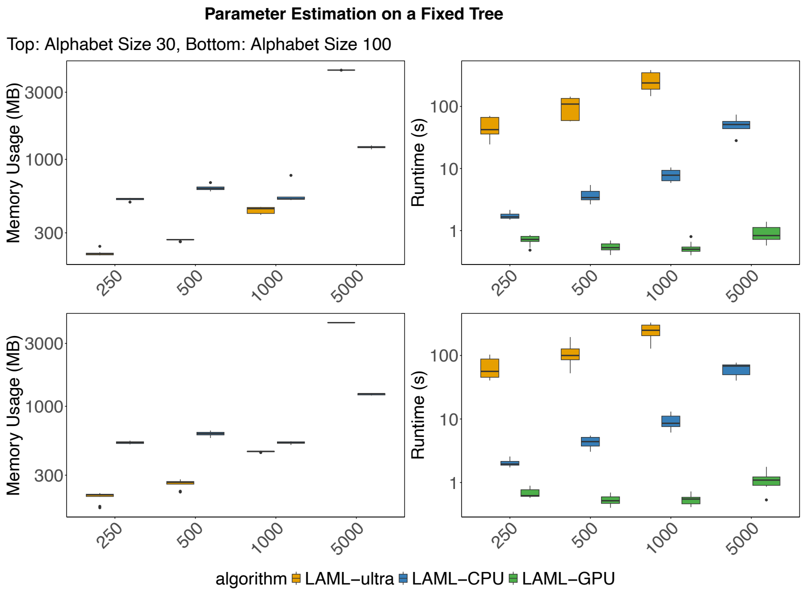

Fig. S6 Memory usage (MB) and runtime (seconds) of LAML parameter estimation on a fixed tree. Results on simulated data with a varying number of cells. All data is simulated under the 25% total missing data model condition, with 50% heritable missing and 50% dropout. LAML-CPU: (no ultrametric constraint, using 8 CPUs), LAML-GPU (no ultrametric constraint, using GPUs), LAML-ultra (LAML with ultrametric constraint, using 8 CPUs). Note that we omit LAML-GPU from the memory usage plot since the -GPU and -CPU versions allocate the same memory.

S1.7.1 Scalability Results

We characterize the scalability of LAML's parameter estimation, across three settings: LAML run on CPUs with the ultrametric constraint (LAML-ultra), LAML run on CPUs without the ultrametric constraint (LAML-CPU), and LAML run on GPUs without the ultrametric constraint (LAML-GPU) (Fig S6). As we expect memory usage (MB) and CPU/GPU utilizationusage % to be the same between LAML-GPU and LAML-CPU, we only report for LAML-CPU. The memory consumption is consistent and reasonable (at 1GB) as the maximum size of the alphabet increases. As the number of nodes increases, the CPU usage % of both LAML-CPU increases. Importantly, we observe that LAML-GPU run without the ultrametric constraint is the fastest, with a runtime of consistently around 1 second even for trees with 5000 nodes. This is consistent with the observation that LAML-GPU converges in under fifteen EM iterations on average (Fig S7), independent of alphabet size.

We evaluate LAML, Cassiopeia-Greedy, Cassiopeia-Hybrid, Neighbor-Joining, and Startle-NNI on increasingly large numbers of cells (Fig S7), and find that LAML uses is able to estimate topologies which improve the RF distance even on 5000 cells in reasonable amounts of time and memory (Fig S7). We note that given 24 hours, Cassiopeia-Hybrid did not produce results on any of the simulated data replicates. In contrast, LAML was able to improve the RF of the trees (Cassiopeia-Greedy: 0.829 RF, LAML: 0.783 RF on 5000 cells).

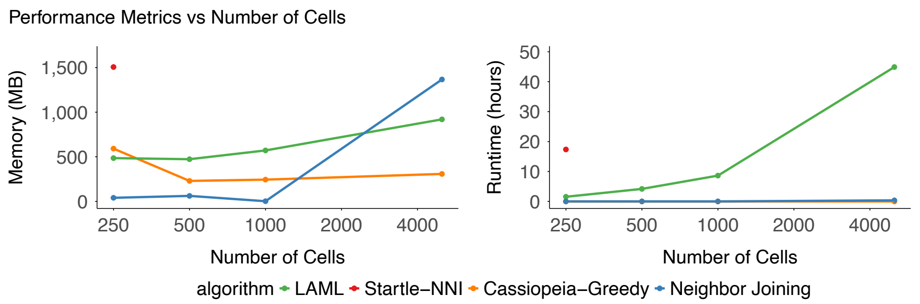

Fig. S7 Comparison of LAML, Startle-NNI, Cassiopeia-Greedy, and Neighbor-Joining in memory usage (in MB) and runtime (in seconds). Results are from simulated data with a varying number of cells, sequence length 30, and 25% missing data of which half is attributed to heritable missing and half is attributed to dropout. All methods were given 24 hours to run (except on 5000 cells, where LAML was given 168 hours). Startle-NNI only finished on the 250 cells in the allotted time with default settings. Cassiopeia-Hybrid did not finish on any of the replicates.

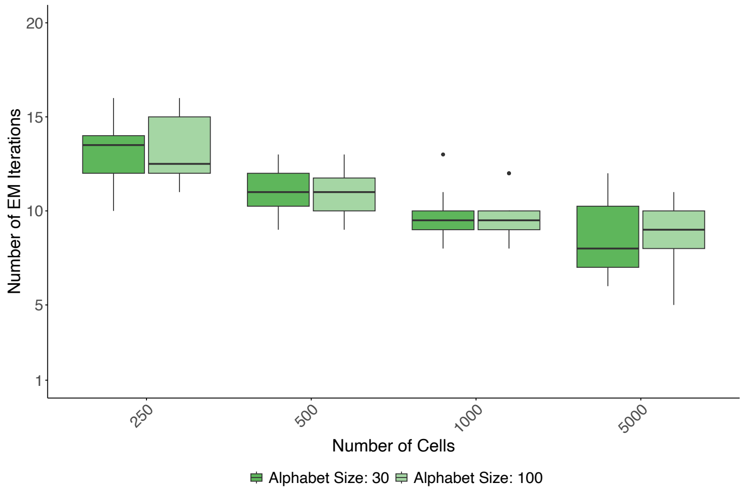

Fig. S8 The number of EM iterations before convergence using LAML-GPU to perform fixed tree parameter estimation over different numbers of cells. All data is simulated with 25% total missing data, with half heritable missing and half dropout.

S1.8 Supplementary Results for KP-Tracer

S1.8.1 Correlation of allelic distances and phylogenetic distances

We use the Mantel test to evaluate the correlation between the indel allelic distances and the phylogenetic distances computed from each inferred tree.

We compute the normalized allelic distance (unweighted) according to the formula implemented in Cassiopeia which considers missing data. Briefly, for a given character we increment the dissimilarity by +2 if the two states are different indels, +1 if one state is unedited and the other is an indel, and +0 if the two states are identical. If one or both states are missing,

no dissimilarity is incurred. The distance is then normalized by the number of non-missing characters across the two samples.

The LAML time-scaled branch lengths are explicitly not proportional to the mutation counts. For a fairer comparison, we use the LAML branch lengths in mutation units (which are output in the *_annotations.txt file). For the parsimony-based (Cassiopeia-Hybrid and Startle-NNI) topologies, we use Sankoff's algorithm [42] to compute mutation counts, which we refer to as the MP branch lengths.

On sample 3432_NT_T1 (which is featured in the main text), we used LAML to estimate branch lengths on the published Cassiopeia-Hybrid tree topology. Then, we compared LAML's branch lengths in mutation units to the MP branch lengths. We observe that the allelic distances have better correlation with the LAML branch lengths than with the MP branch lengths (LAML: 0.860, MP: 0.732, all with p-values of 0.001). Across the other five samples, we see that allelic distances have comparable correlation with the LAML and MP branch lengths.

Sample Name	LAML branch lengths	MP branch lengths	Number of Cells
3703_NT_T3	**0.9761**	0.9533	23
3703_NT_T1	0.9142	**0.9479**	74
3703_NT_T2	**0.6561**	0.2420	103
3520_NT_T1	**0.8990**	0.8678	144
3432_NT_T1	**0.8600**	0.7322	174
3435_NT_T4	0.8266	**0.8717**	220

Table S2 Mantel test between edit distances (using missing-aware Hamming distance) and two types of phylogenetic distances (LAML and MP branch lengths) on a fixed tree topology across six samples.

We selected the largest inferred metastasis event (i.e. the metastasis event with the largest number of extant cells in the subtree) for further investigation. Specifically, the metastasis event from the primary tumor to the soft tissue metastasis site has 594 extant cells under the parent node and 495 extant cells under the child node (referred to as the metastasis clade) in the LAML-inferred tree. Consistent with our observation that the LAML tree requires fewer cell migration events than the Cassiopeia-Hybrid tree, we observe that the largest metastasis clade in the LAML tree has a higher purity of anatomical site locations than the largest metastasis clade in the Cassiopeia-Hybrid tree (LAML: 87.5%, Cassiopeia-Hybrid: 84.72%). We further observe that the grouping of extant cells into the LAML metastasis clade (lineage group: blue) is supported by a distinct indel acquired at the third target site (red box, Fig S9). In contrast, sibling cells that were not inferred to metastasize (lineage group: yellow) share different indels at that same target site.

We evaluated the indel patterns for the extant cells under inferred migration events in the LAML and Cassiopeia-Hybrid trees. For a metastasis event on a branch, we computed the average allelic distance across extant cells below the child node. For clades containing at least two extant cells, we observe lower allelic distance in LAML 's metastasis clades than in the Cass-Hybrid metastasis clades, despite the LAML metastasis clades also being larger (LAML: mean size μ : 0.203, median size $\tilde{\mu} = 0.166$; Cass-Hybrid: $\mu = 0.249$, $\tilde{\mu} = 0.222$).

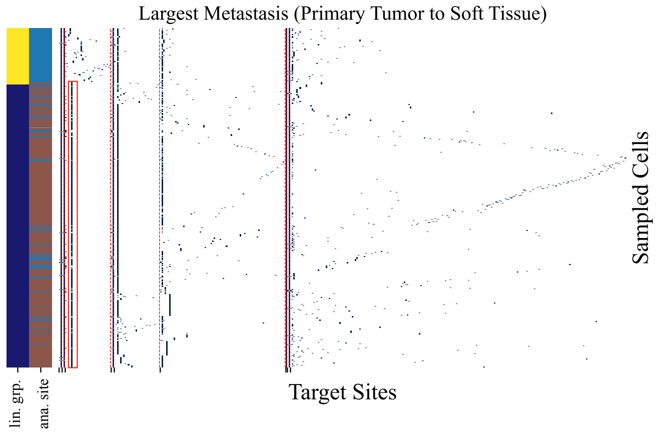

Fig. S9 Illustration of sampled cells by clustered binarized target sites, for extant cells associated with the largest metastasis in the LAML tree. This includes all extant cells under the parent node, incident to the branch with the largest metastasis event. The lineage group (lin. grp) column summarizes whether the cells were in the metastasis clade (blue) or in the sibling clade (yellow). The anatomical site (ana. site) column illustrates the extant cells' anatomical origins: soft tissue (brown), primary tumor (blue). Each row is a cell, and each column is the categorical edit for a specific target site (over 9 target sites). The presence of a categorical edit is shown in dark blue and absence of an edit is shown in white. Red dashed lines indicate the start of each target site. Red box labels the distinct indel acquired in the third target site.

S1.8.2 Variations on the Migration Cost

The KP-Tracer sample (`3724_NT_ALL`) includes two useful meta annotations on the observed cells. First, we have the anatomical site labeling (e.g. primary tumor, lung metastasis 1, lung metastasis 2, lung metastasis 3, soft tissue metastasis). This indicates where in the body each cell was sampled from. Second, we have a "refinement" of this anatomical site labeling for certain tumors, that indicates the approximate spatial location of the sample in that tumor (e.g. primary tumor can be broken into 15 components).

As defined above, a migration graph is defined by the tree and the clustering. Thus, given a tree topology T and a clustering SC for spatial clustering and AC for anatomical clustering, we can get migration graph $T + SC \to MG_{SC}$ and $T + AC \to MG_{AC}$. We presented results on the anatomical clustering in the main body of the paper. We include additional results on MG_{SC}, the and compute different costs on this migration graph (below) in the Supplementary Results section for KP-Tracer S1.8.

1. Total Cost: The migration cost of $MG_{SC}(T_{LAML})$ and $MG_{SC}(T_{CassH})$.
2. Induced Anatomical Cost: Using the parsimonious ancestral labeling inferred using the spatial clustering SC, infer the number of transitions between anatomical sites.
3. Spatially-aware Migration Cost: Within the **primary tumor only**, each transition is weighted as follows: migration cost $*$ length of path between spatial locations.
4. Constrained Parsimony: Given a tree topology T, we first compute a parsimonious ancestral labeling solution given the anatomical clustering AC. Then, for clades within each anatomical site, we compute a parsimonious ancestral labeling solution given the spatial clustering SC.

	Migration Types	LAML	Cass-Hybrid	Startle-NNI
De-duplicated (1,461 cells)	All migrations	**99**	136	119
	Reseeding	**42**	68	56
Full Tree (21,108 cells)	All migrations	**1568**	1600	1783
	Reseeding	**191**	219	203

Table S3 Migration cost of the trees inferred by Cass-hybrid, Startle-NNI, and LAML on KP-tracer sample 3724_NT_T1_All.

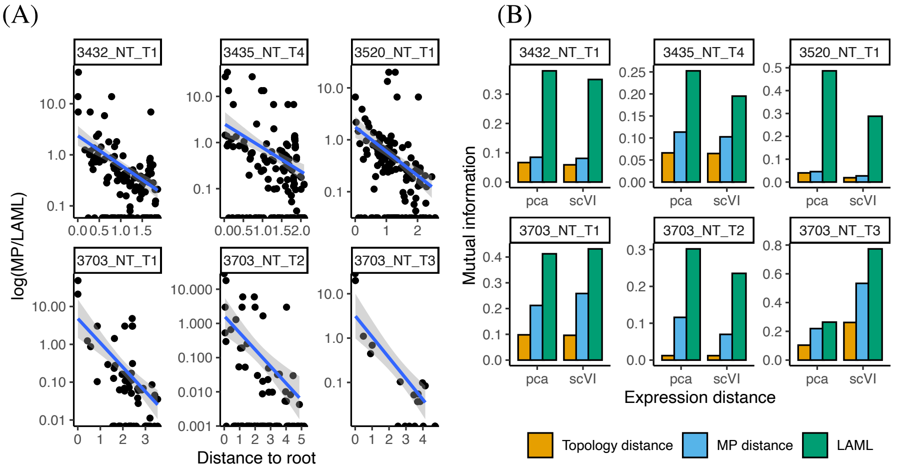

Fig. S10 Branch length estimation by LAML (dML) and Maximum Parsimony (dMP) on the 6 selected samples of KP-tracer. (A) The ratio of each cell's branch length (i.e. the branch above the node) estimated by maximum parsimony (dMP) and maximum likelihood (dML) versus its distance to the root. The y-axis is shown in log-scale. (B) Mutual information of pairwise gene expression and phylogenetic distances. Gene expression is represented either by PCA, scVI, or UMAP. For these 6 samples, we use the published tree topology (estimated by Cassiopeia) and estimate branch lengths by LAML (ML distance), maximum parsimony (MP distance), or solely use the tree topology (Topology distance).

Metrics	LAML (T_{LAML})	Cassiopeia-Hybrid (T_{CassH})
Total Cost	836	959
Induced Anatomical Cost	125	159
Spatially-aware Migration Cost (Primary Tumor)	730	828
Constrained Parsimony	935	1095

Table S4 Metrics computed on the MG_{SC} migration graph with spatial clustering of all observed cells.

From Table S4, we can observe that the LAML tree topology always achieves a lower migration cost than the Cassiopeia-Hybrid tree does.

S1.8.3 Metastasis Analysis

With the knowledge that this experiment ran for 6 months, we scale our ultrametric tree, which had root-to-tip distance of 2.47 to be 6 with a scale factor of 2.425. To produce the bottom half of Figure 4(B), we begin with our tree with inferred anatomical labels on the internal nodes as described in the main text [45] and in Supplementary Section 1. We annotate each edge

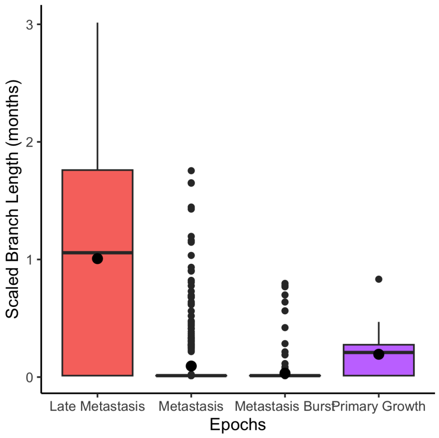

Fig. S11 Length of branches lying entirely within each epoch.

$e = (u, v)$ based on the anatomical labels of the parent node u and child node v. We use edge, branch and lineage interchangeably in this section.

Notably, we are interested in transition edges, where one of the nodes is labeled with the primary tumor anatomical site, and the other node is labeled with one of the other 4 anatomical sites. Thus, we define four edge types: non-transition edge, primary to non-primary (metastasis), non-primary to primary (reseeding), and non-primary i to non-primary j (where $i \neq j$).

Next, we establish $2,470$ intervals ranging from 0.0 to 2.47 with a step size of 0.001. In each interval, we find the branches which overlap with this interval. Thus, we can compute the number of each branch type which overlap with this interval. Since the tree grows to include more cells in each cell generation, we normalize the counts of each edge type by the total number of branches overlapping this interval.

We can do an additional analysis to compute the expected number of unedited sites left at any given time point. We use the following equation: $\exp(-d/c) * 9$, where d is the time-scaled distance to root, and c is our scaling factor. It is useful to consider the expected number of unedited sites left at various time points (i.e. at the 3 month mark in the main paper), to give a sense of whether there is still phylogenetic signal left.

We also analyze the length of any branches which lie entirely within a single epoch in Figure S11. Note that the branches in the Late Metastasis epoch are much longer as expected due to the loss of phylogenetic signal and subsequent low tree resolution. The branch lengths observed in the the Primary Growth epoch are shorter, suggesting the phylogenetic signal is capturing cell divisions. Interestingly, the Metastasis epoch has the shortest branches < 1.0 month, perhaps suggesting a general increase in cell division rates. The Metastasis Burst window within the Metastasis epoch has even fewer outlier branch lengths.

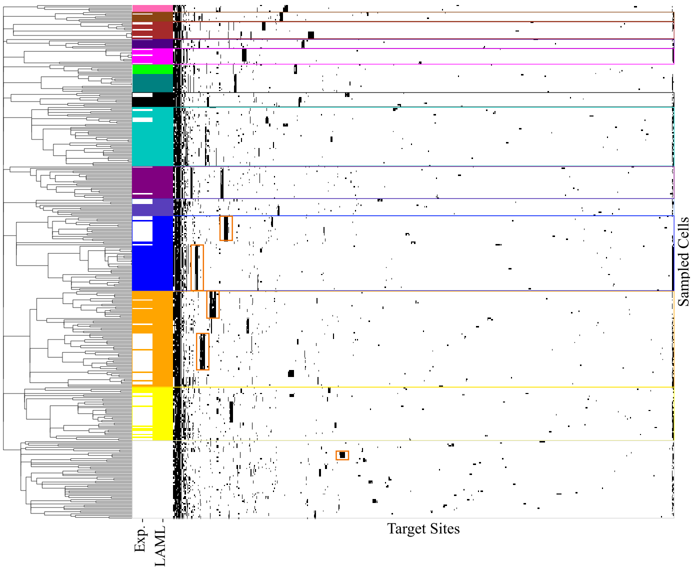

Fig. S12 Illustration of sampled cells by clustered binarized target sites in Sample 12. Black indicates the presence of this unique indel in a specific target site. The first column "Exp" indicates the experimentally determined progenitor label with white indicating a missing label. The second column "LAML" indicates the LAML determined progenitor label, with black indicating LAML did not infer clade membership for this cell. The LAML inferred phylogeny is shown on the left. Colored boxes are placed around those clades where LAML imputed one or more cell progenitor labels. Red boxes highlight clades with clear indel patterns.

S1.9 Supplementary Results for the mouse embryo cellular development using TLS

We further investigated the placement of the cells with missing progenitor labels, taking Sample 12 as an example below. Cells with missing progenitor labels are placed in different progenitor clades, in addition to the "newly discovered group" of cells which lack their progenitor label (Fig S12). We highlight the blue and orange progenitor groups; each group has two clear clades in the lineage tree which are accompanied by a clear shared indel (Fig S12). In the clade with missing progenitors, we observe a clade of missing progenitor cells which are supported by a clear indel pattern (Fig S12, last red box).

For all 160 cells in Sample 12 which are missing their progenitor label, all are placed with their closest allelic distance sister(s) in the LAML inferred tree. For example, two cells "TCAAGTGGTGGTCTCG-1" and "TTCTGTAGTCTTGTCC-1" with missing progenitor label were imputed to be in clade 12 because of indel similarity with the cell "AAGTGAAAGGATGCGT-1," which has progenitor label 12. Table A4 gives the exact number of cells with missing progenitor label reassigned by LAML.

We list below the progenitor discordance and negative log-likelihood across samples and across trees.

Lentiviral Barcode	?	14	10	4	2	7	37	13	12	15	18	34	23	29	22
Original	160	32	11	35	36	8	8	7	8	21	13	7	6	6	5
LAML	55	68	38	53	42	12	12	10	11	23	13	7	7	7	5

Table S5 The first row gives the lentiviral barcode label, the second row shows the number of cells with this label according to the experimentally determined dataset, and the last row shows the number of cells with this label according to the LAML inferred tree. Note that the lentiviral barcode columns are in descending order according to the number of cells reassigned (from "?" or to a non-missing label).

Sample	Progenitor discordance			Negative log-likelihood		
	Cassiopeia	Startle	LAML	Cassiopeia	Startle	LAML
10	29%	35%	0	10,661	11,156	10,656
11	39%	40%	0	8,433	8,567	7,992
12	39%	40%	0	14,311	14,786	13,666
21	41%	44%	0	10,419	10,739	9,720
23	37%	37%	0	10,936	11,631	10,403
24	32%	30%	0	6,260	6,420	5,965

Table S6 Progenitor discordance and negative log-likelihood of the Cassiopeia and LAML cell lineage trees on the TLSCL data of Mouse embryo with multi-progenitors. Progenitor discordance is measured by normalized triplet error, which ranges from 0 to 1 (see Methods for more details). Negative log-likelihood is computed by LAML for each tree topology after optimizing for branch lengths, editing rate, dropout probability, and heritable missing rate.

Sample	No. known progenitors	No. discovered progenitors	No. given progenitor labels	No. imputed progenitor labels
10	14	2	173	132
11	11	2	82	124
12	14	1	203	160
21	8	0	204	97
23	16	1	220	111
24	7	3	71	121

Table S7 Summary of progenitor imputation and discovery of LAML on TLSCL.

The developmental tree we used to compute the development cost is constructed from the TLS paper [26], specifically using details from in notebook 8 of the AM-DNA-097 analysis, titled "progenitor analysis". Fig S14 shows a visual comparison of two TLS1 tree topologies on TLS1 ($1,836$ cells). We corroborated this tree through discussion with the authors of [26] and checked for compatibility against a development tree from an in vivo mouse experiment [47].

S1.10 Supplementary Results for the mouse embryo cellular development using intMEMOIR

Comparison with TiDeTree on all the intMEMOIR samples. Note that the TiDeTree topologies shown here are the maximum credibility clade trees obtained from the TiDeTree authors, which show trees jointly estimated (sharing parameters across all samples). We ran Cassiopeia-Hybrid with a cut-off of 100 cells, so that we effectively report results for Cassiopeia-ILP on all intMEMOIR samples.

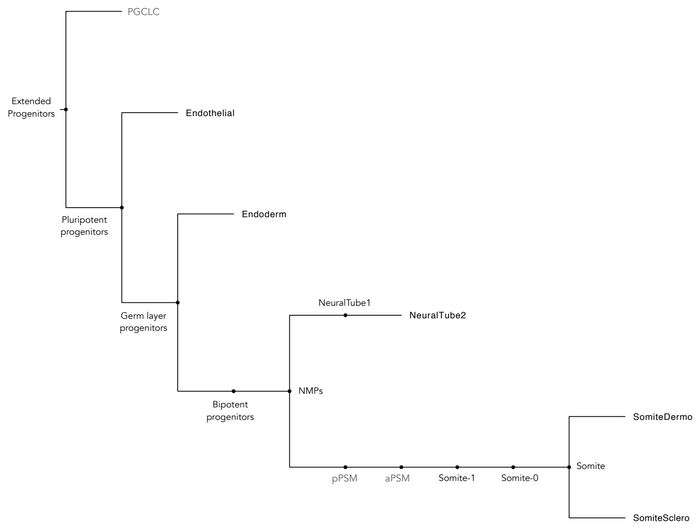

Fig. S13 The developmental tree relating cell states, annotated with progenitors defined in the TLS paper [26]. In the resolved tree with 17 annotated cell types, all edges between nodes are given branch length of 1. In the grouped tree with only 5 differentiated cell types, all edges below the NMP internal node are given length 0.

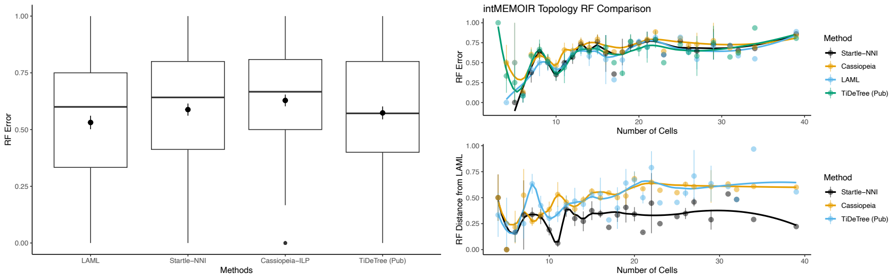

Fig. S15 Comparison of LAML, Startle-NNI, Cassiopeia-ILP and TiDeTree. (Left) Topology error (RF) comparison between the true tree and trees estimated using LAML and the benchmarked methods, on all intMEMOIR samples. (Right) RF distance plotted against the varying number of cells. The top plot shows RF distance from the true tree and the bottom plot shows RF distance from the LAML tree. Note that we run Cassiopeia-ILP-intermediate (non-default settings) here.

Runtime comparison on the intMEMOIR data (see Fig S16).

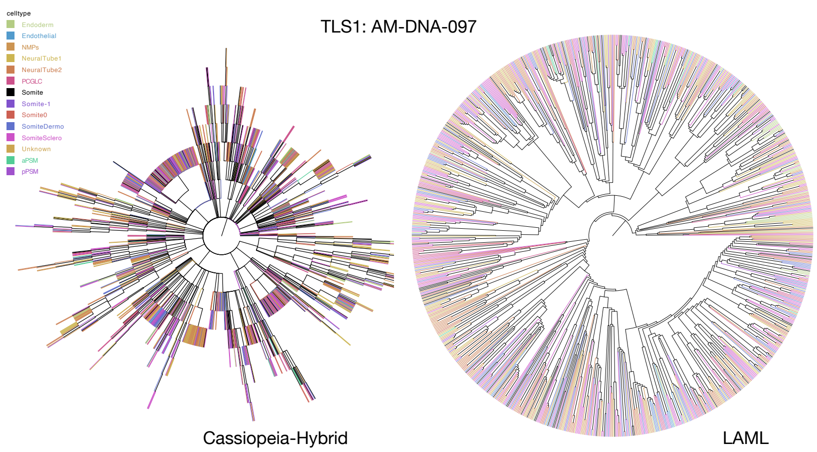

Fig. S14 Visual comparison of two trees built on the TLS1 (AM-DNA-097) data [26] via Cassiopeia-Hybrid and LAML. In both trees, branches incident to the leaves are colored by annotated cell type [26]. The left tree is the published Cassiopeia-Hybrid tree topology, and the right tree is the time-scaled LAML tree topology.

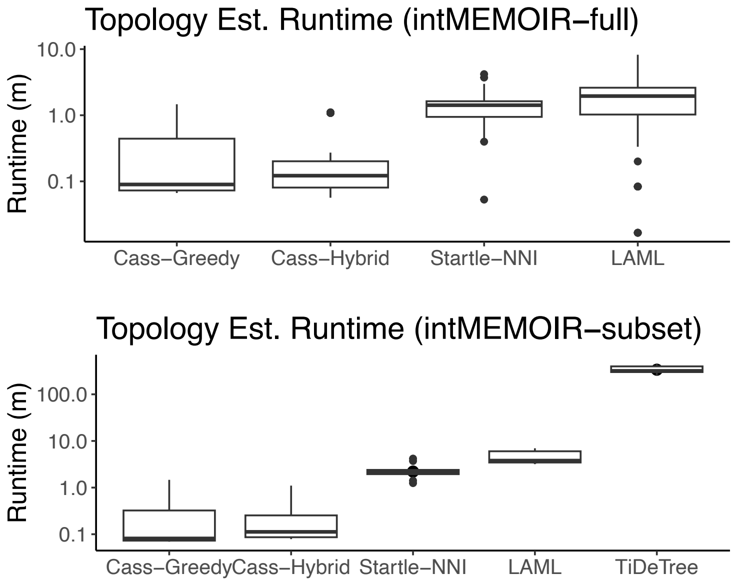

Fig. S16 Topology estimation runtime comparison between LAML and TiDeTree on intMEMOIR. Note that the runtime of each methods' starting tree has also been added to that method's runtime. Please note also that LAML was run while enforcing the ultrametric constraint at each step.

S1.11 Distance-Based Exploration

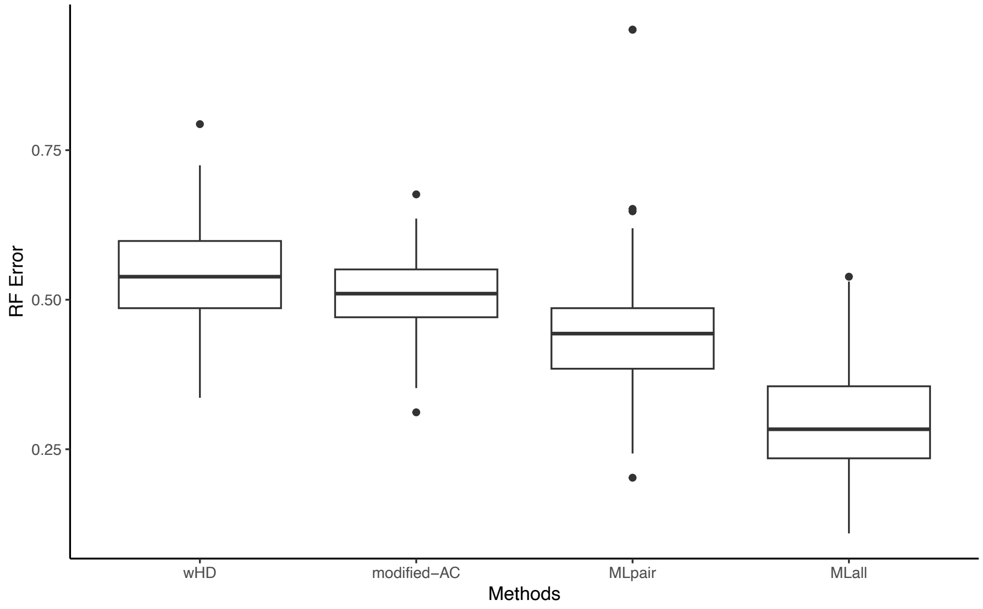

Fig. S17 Comparison of different approaches to distance estimation. Two pipelines: (1) From the left, the first three pipelines first estimate rooted pairwise distances, before tree estimation using NJ. (2) MLall (LAML) finds the maximum likelihood distance matrix and tree topology over all pairs of leaves at once.

Figure S17 illustrates how distances estimated under this statistical model improve tree estimation. wHD, which does not take the missing state into consideration, produces trees with the highest RF error across all methods. This can be contrasted with the mAC, which does consider the missing state. The MLpair approach, which explicitly models the two types of missing data for each pair of cells, further improves the RF error. As expected, the MLall approach, which is able to optimize distances between sequences over all pairs of sequences, achieves the lowest RF error. This indicates the importance of estimating distances under a statistical model explicitly modeling the two types of missing data.

S1.12 Review of Lineage Tracing Technologies

Dynamic lineage tracing technologies begin by engineering one or more progenitor cells with artificial recording sites, then accumulating heritable insertions or deletions (“indels”) [14] over cell generations. We classify dynamic lineage tracing technologies into 3 generations of technology. In the first generation of dynamic lineage tracing (GESTALT[13] and MEMOIR [16]), the recordable time is low because of limited number of target sites (no more than 10) and edit states (limited to 2: inversion and deletion). In the second generation (scGESTALT [17], LINNAEUS [18], ScarTrace [19], and intMEMOIR[20]), other information such as gene

expression and spatial information, are recorded in addition to CRIPSR-Cas9 edit, enhancing tree inference [17]. However, the recordable tree depth remains low (only 6 cell generations) [13, 17], and there is a high error rate as edits are often removed or overwritten [14, 18]. In the third generation, novel dynamic lineage tracing technologies, such as CARLIN [21], iTracer [22], Chan et al. [4], Yang et al. [23] and TypeWriter [25], substantially improve recordable tree depth. We can further classify these technologies into two groups. The first group comprises technologies that induce editing at distinct time points. The second group comprises TypeWriter [25], Chan et al. [4], Quinn et al. [24], and Yang et al. [23], making up a group of technologies leveraging continuous expression of the editor to increase the number of cell generations recorded by optimizing the number of editable sites and states. According to TypeWriter, the recordable tree depth can scale to 20 generations. The technology developed by Chan et al. is later extended in the publications by Quinn et al. [24] and finally Yang et al. [23], where the number of target sites and edit states scale up to hundreds, and the recordable tree depth scales to 15 generations. Notably, the largest sample has 21,108 cells spanning 5-6 months of cell growth.

We should note that although the number of editable target sites affects the number of cell generations one can record over, that the quality of target sites affect how many of them turn out to be informative. Note also the distinction between continuous editing and inducible editing approaches: the depth of a given reconstructed cell lineage tree may span different numbers of cell generations and developmental time. **PMM is applicable to *all* of these lineage tracing technologies.**

Lineage Tracing Technology	Lineage Tree Depth	Time Recorded Over	# Recording Target Sites	Edit Location	Size of Edit Library	Genome Editor
Kalhor2018 [1]	3-5	6-8 weeks	41-60	scattered in genome	uncontrolled	constitutive Cas9
NSC-seq [2]	10	6-8 weeks	9	scattered in genome	uncontrolled	constitutive Cas9
ScarTrace [19]	2	10 hours	8	target array	uncontrolled	Cas9 injection
LINNAEUS [18]	6	10 hours	16-32	CFP region	uncontrolled	Cas9 injection
CARLIN [21]	3	/	10	target array	uncontrolled	dox inducible
iTracer [22]	5	100 hours	4 (Fig 6., Ext. Fig 10.)	target array	uncontrolled	dox inducible
scGESTALT [17]	5	/	9	target array	uncontrolled	heat shock inducible
Chan et al. [4]	12	6 days	9-45	target array	uncontrolled	constitutive Cas9
Quinn et al. [24]	mean depth 7.5	2 months	30	target array	uncontrolled	constitutive Cas9
Yang et al. [23]	15	6 months	10-30	target array	uncontrolled	constitutive Cas9
intMEMOIR [20]	4	36 hours	no target sites 10 transgenes	integrated target barcode	uncontrolled	heat shock inducible
DNATypewriter [25]	20	25 days	48 ”tapes”	target “tape” array	by design: prototyped up to 16	dox-inducible prime editing

Table S8 Summarizing key properties of different lineage tracing technologies.

S1.13 Review of Other Computational Approaches

Computational methods for lineage tracing can be broadly categorized into three groups: maximum parsimony (MP), distance-based, and probabilistic modeling, with some methods exhibiting hybrid characteristics. Maximum parsimony methods [27, 30] rely on the heuristic criterion of maximum parsimony (MP). Cassiopeia [27], a highly regarded method in the DREAM challenge [15], infers the MP tree using a modified Camin-Sokal (C-S) model to leverage the non-modifiability property of CRISPR/Cas9 edits. Sashittal et al. [30] later refer to this evolutionary model as the “star homoplasy model” and develop Startle [30] to find the MP tree. However, due to the use of the heuristic MP criterion, neither Cassiopeia or Startle can adequately capture the stochastic nature of the CRISPR/Cas9 process. The second category

encompasses distance-based methods, such as neighbor joining and triplet-based approaches. A critical challenge in these methods lies in computing pairwise distances, which are essential for generating the distance matrix (for neighbor joining) or triplet topologies (for triplet-based methods). Recent research [34] has shown that accurate computation of distances can be achieved with theoretical guarantees, but only when a probabilistic model is assumed. The last category comprises methods that formulate lineage tracing as a statistical inference problem. Bayesian [32] and maximum likelihood [13, 31, 33] methods fall within this category. Many of these methods model a specific set of lineage tracing technologies and do not generalize well to other technologies. For example, GAPML [31] introduced a probabilistic model tailored for the scGESTALT technology, focusing on single-barcode evolution. LiNTIMaT [33] can only be used for the second generation of lineage tracing technologies, where there are only two edit states, and requires expression data as input. TiDeTree [32] is designed more generally for barcode-based technologies, but their model assumes sites are independent and *identically distributed*, limited its application to the handful of technologies having all target sites shared a same set of edit states.
