# Supplementary material for: Maximum likelihood inference of time-scaled cell lineage trees with mixed-type missing data using LAML
Source: Genome Biol. 2025 Jul 2;26:189. doi: 10.1186/s13059-025-03649-9 (PMC12220811; doi:10.1186/s13059-025-03649-9)
Supplement: Supplementary file 2 — Supplementary Material 2: Supplementary methods. Discusses computation of the tree likelihood, a derivation of the EM algorithm, and proofs and algorithms associated with improving the EM complexity. [file 13059_2025_3649_MOESM2_ESM.pdf]

S2: Supplementary methods

Maximum Likelihood Inference of Time-scaled Cell Lineage Trees with Mixed-type Missing Data using LAML

Gillian Chu[1,†] Uyen Mai[1,†] Henri Schmidt[1] Benjamin J. Raphael[1,*]

[1]Department of Computer Science, Princeton University, NJ 08540, USA
[†] These authors contributed equally to this work.

[*]Correspondence: braphael@princeton.edu

Appendix S2 Supplementary methods

Contents

S2.1 Extended notations 1
S2.2 Computing the log-likelihood given T and Θ 2
S2.3 The edge-inside likelihood, the outside likelihood, and the posterior 3
S2.3.1 The edge-inside likelihood 3
S2.3.2 The outside likelihood 4
S2.3.3 The posterior 4
S2.4 Derivation of the EM algorithm (Proof of Eq. 14 in the main text) 5
S2.5 Reducing the complexity of the E-step 6
S2.5.1 Special properties of α-clades and z-branches 7
S2.5.2 The linear-time algorithm 15
S2.6 Solving the M-step 21
S2.6.1 Proof of convexity by variable blocks 21
S2.6.2 The block coordinate ascent algorithm 21

S2.1 Extended notations

Consider a dynamic lineage tracing procedure consisting of K target-sites. At the beginning of the experiment, there is exactly one *progenitor cell* that has all target-sites in the *unmutated state*, denoted by 0. During the genome editing process, each target-site k of any cell can either mutate into one of the $M^{(k)}$ *mutated states* in $\{1, ..., M^{(k)}\}$ or go to the *silent state*, denoted by -1. During the single-cell sequencing (sc-Seq) process, cells in the silent state cannot be determined and will show up in the *missing state*; in addition, cells in the other states can also turn into the missing state because of dropout. We denote the missing state by "?" and refer to the set $\mathscr{A}^{(k)} = \{?, -1, 0, 1, ..., M^{(k)}\}$ as the *alphabet* of the target-site k. We assume that there are N cells sequenced by sc-Seq, so the dynamic lineage tracing data consists of $N \times K$ entries. Following the convention in phylogenetics, hereafter, we refer to *target-site* as *site* for brevity.

We represent the cell lineage of the lineage tracing data by a rooted tree T. Let $\mathscr{L}_T$, $\mathscr{V}_T$, and $\mathscr{E}_T$ be the *set of leaves*, *set of nodes*, and *set of edges* of T, respectively. Let (u, v) be the edge in $\mathscr{E}_T$ from u to its child v (where $u, v \in \mathscr{V}_T$), and r_T be the root of T. Unless otherwise specified, e is used to denote the edge connecting u to v. Throughout this paper, we use *edge* and *branch* interchangeably. We assume that the root of T has exactly one child and all of its other internal nodes has exactly two children. For each node $v \in \mathscr{V}_T$, we let T_v be the clade of T rooted at v, $\mathscr{C}_T(v)$ be the leafset of T_v, and $\tilde{\mathscr{C}_T}(v) := \mathscr{L}_T \setminus \mathscr{C}_T(v)$. When the context is clear, the subscript T can be omitted for brevity.

Throughout this paper, we will use the following two conventions for random/realization matrices/vectors: (1) blackboard-bold letters (e.g. $\mathbb{A}, \mathbb{a}, \mathbb{B}, \mathbb{b}, \mathbb{C}, \mathbb{c}$) will be used to indicate a *random* entity while normal bold letters (e.g. **A,a,B,b,C,c**) will be used to indicate a realization. (2) the uppercase letters (e.g. $\mathbb{A}, \mathbb{B}, \mathbb{C}$ and **A,B,C**) are reserved for matrices while lowercase letters (e.g. $\mathbb{a}, \mathbb{b}, \mathbb{c}$ and **a,b,c**) are reserved for vectors. For a set $\mathscr{S}$ of N cells with K sites, we define the genome-edited sequences of $\mathscr{S}$ by a $N \times K$ matrix and refer to it as the *cell-state representation matrix* of $\mathscr{S}$, which has rows indexed by $\mathscr{S}$ and columns indexed by the sites.

We let $\mathbb{X}$ be the cell-state representation random matrix of $\mathscr{V}_T$ *before sc-Seq*, and **X** be its realization. Similarly, we let $\mathbb{D}$ be the cell-state representation random matrix of $\mathscr{L}_T$ *after sc-Seq*, and **D** be its realization. Note that entries of **X** take values in $\mathscr{A}^{(k)} \setminus \{?\}$ while entries of **D** take values in $\mathscr{A}^{(k)} \setminus \{-1\}$. In dynamic lineage tracing, $\mathbb{D}$ is observed exactly once and $\mathbb{X}$ is hidden. Therefore, throughout this paper, **D** represents the lineage tracing data, which is also the sole *observed* realization of $\mathbb{D}$. Borrowing the terminology from previous works [23, 30], we also refer to **D** as the *character matrix*.

We use the following conventions for any cell-state representation matrix, both random and realization. First, the superscript $\bullet^{(k)}$ is used to denote any entity associated with site k. For example, $\mathbb{X}^{(k)}$ denotes the kth column of $\mathbb{X}$. Second, we use $\bullet^{(k)}(v)$ to refer to the element of the cell-state representation matrix associated with the row index v and column index k. For example, $\mathbb{X}^{(k)}(v)$ denotes the random variable in $\mathbb{X}$ that is associated with node v and site k. Finally, if **M** is a cell-state representation matrix and $\mathscr{S}$ is a subset of the row indices of **M**, then we let $\mathbf{M}|_{\mathscr{S}}$ be the matrix constructed from the rows of **M** whose indices belong to $\mathscr{S}$. The same convention is applied to $\mathbb{X}$, $\mathbb{D}$, and their realizations. Finally, for the sole realization **D** of $\mathbb{D}$, we let $\mathbf{D}_v$ be the shorthand for $\mathbf{D}|_{\mathscr{C}_T(v)}$ and $\tilde{\mathbf{D}}_v$ be the shorthand for $\mathbf{D}|_{\tilde{\mathscr{C}}_T(v)}$.

Table S9 List of Notations

Notations	Explanation
N	Number of cells
K	Number of sites
$M^{(k)}$	Number of mutated states associated with site k
$\mathscr{A}^{(k)}$	Alphabet of site k
T, r_T	Cell lineage tree and its root
$\mathscr{V}_T, \mathscr{E}_T, \mathscr{L}_T$	Set of nodes, edges, leaves of tree T
$\mathscr{C}_T(v)$	Set of leaf indices under node v of tree T
$\mathbb{X}, \mathbf{X}$	Random matrix and its realization
$\mathbb{x}, \mathbf{x}$	Random vector and its realization
$\mathbb{X}^{(k)}, \mathbf{X}^{(k)}$	Random vector associated with site k and its realization
$\mathbb{X}_i^{(k)}, \mathbf{X}_i^{(k)}$	Random entry with index i associated with site k and its realization
$\mathbb{X}^{(k)}(v), \mathbf{X}^{(k)}(v)$	Random entry associated with site k and node v, and its realization
**X, D**	Realized values of random matrices $\mathbb{X}, \mathbb{D}$
$\mathbf{X}^{(k)}, \mathbf{D}^{(k)}$	Realized Random vector associated with site k
$\mathbf{Q}^{(k)}$	Rate matrix at site k
$\mathbf{\Psi}^{(k)}$	Transition matrix at site k
$\mathbf{\Phi}$	Transition matrix for dropout
δ_e	Length of branch e
ν, ϕ	Heritable missing rate and dropout probability

S2.2 Computing the log-likelihood given T and Θ

The log-likelihood can be computed in linear-time using Felsenstein's pruning algorithm [57]. In this section we show that the pruning algorithm can be applied to our model by presenting recurrence equations.

To describe his algorithm, Felsenstein introduced the concept of *partial likelihood* at a node in the tree. Here we use the term *inside likelihood* (see Definition 1) to refer to the partial

likelihood described by Felsenstein, for the purpose of distinguishing it from the *outside likelihood* that will be defined later. Consider a node $u \in \mathscr{V}_T$. The *node-inside likelihood of* u is defined as follows:

Definition 1. *The node-inside likelihood (a.k.a partial likelihood) of u at site k w.r.t a realization α_u of $\mathbb{X}^{(k)}(u)$, denoted by $\mathcal{L}^{(k)}_{\text{node}}(u, \alpha_u; T, \mathbf{\Theta})$, is the likelihood of observing $\boldsymbol{D}^{(k)}_u$ given $\mathbb{X}^{(k)}(u) = \alpha_u$. In other words:*

$$\mathcal{L}^{(k)}_{\text{node}}(u, \alpha_u; T, \mathbf{\Theta}) := \mathcal{P}(\boldsymbol{D}^{(k)}_u | \mathbb{X}^{(k)}(u) = \alpha_u; T, \mathbf{\Theta}) \quad \text{(S1)}$$

The node-inside likelihoods of all nodes u at all states $\alpha_u \in \mathscr{A}^{(k)}$ can be computed in one bottom-up tree traversal, using the following recurrence:

$$\mathcal{L}^{(k)}_{\text{node}}(u, \alpha_u; T, \mathbf{\Theta}) = \begin{cases} \mathbf{\Phi}(\alpha_u, \mathbf{D}^{(k)}_u(u)), \text{ if } u \in \mathscr{L}_T \\ \prod\limits_{v \in \mathscr{C}_T(u)} \sum\limits_{\alpha_v \in \mathscr{A}^{(k)}} \mathbf{\Psi}^{(k)}_e(\alpha_u, \alpha_v) \mathcal{L}^{(k)}_{\text{node}}(v, \alpha_v; T, \mathbf{\Theta}), \text{ otherwise} \end{cases} \quad \text{(S2)}$$

In the PMM model, $\mathbb{X}^{(k)}(r_T)$ can only take state 0. Therefore, the full likelihood $\mathcal{P}(\mathbf{D}^{(k)}; T, \mathbf{\Theta})$ is simply the node-inside likelihood of the root node r_T w.r.t. state 0. In other words, for all site k we have:

$$\mathcal{P}(\mathbf{D}^{(k)}; T, \mathbf{\Theta}) = \mathcal{L}^{(k)}_{\text{node}}(r_T, 0; T, \mathbf{\Theta})$$

Thus, the full likelihood can be computed in one bottom up traversal, following Felsenstein's. The complexity is therefore $\mathcal{O}(NKM^2)$ where N is the number of cells, K is the number of target sites, and M is the maximum alphabet size. As we will show in this section, we improve this complexity to a linear-time algorithm $\mathcal{O}(NK)$.

S2.3 The edge-inside likelihood, the outside likelihood, and the posterior

In this section, we define the **outside likelihood** and the **posterior** probabilities, which can be used later. We will show equations relating the inside likelihood, outside likelihood, and the posterior, and present a linear-time algorithm to compute all these entities for a tree (i.e. an extended version of the Felsenstein's prunning algorithm). We note that similar equations have been previously described in the literature (see [59] for example). Below we show the definitions and recurrence equations, but skip the proofs for brevity.

S2.3.1 The edge-inside likelihood

We first define the *inside likelihood of a branch*, as follows:

Definition 2. *The edge-inside likelihood of $e = (u, v)$ at site k w.r.t. a realization α_u of $\mathbb{X}^{(k)}(u)$, denoted by $\mathcal{L}^{(k)}_{\text{edge}}(e, \alpha; T, \mathbf{\Theta})$, is the probability of observing $\boldsymbol{D}^{(k)}_v$ given $\mathbb{X}^{(k)}(u) = \alpha_u$. In other words:*

$$\mathcal{L}^{(k)}_{\text{edge}}(e, \alpha_u; T, \mathbf{\Theta}) := \mathcal{P}(\boldsymbol{D}^{(k)}_v | \mathbb{X}^{(k)}(u) = \alpha_u; T, \mathbf{\Theta}) \quad \text{(S3)}$$

The edge-inside likelihood of $e = (u, v)$ at any state α_u can be computed by summing over the inside likelihoods of v at all states α_v, as follows:

$$\begin{aligned}\mathcal{L}^{(k)}_{\mathsf{edge}}(e, \alpha_u; T, \mathbf{\Theta}) &= \sum_{\alpha_v \in \mathscr{A}^{(k)}} \mathbf{\Psi}^{(k)}_e(\alpha_u, \alpha_v)\mathcal{P}(\mathbf{D}^{(k)}_v | \mathbb{X}^{(k)}(v) = \alpha_v; T, \mathbf{\Theta}) \\ &= \sum_{\alpha_v \in \mathscr{A}^{(k)}} \mathbf{\Psi}^{(k)}_e(\alpha_u, \alpha_v)\mathcal{L}^{(k)}_{\mathsf{node}}(v, \alpha_v; T, \mathbf{\Theta})\end{aligned} \tag{S4}$$

Using Eq. S4, we can extend the bottom up traversal to also compute the inside likelihoods at all branches.

S2.3.2 The outside likelihood

Definition 3. *The outside likelihood of a node v at site k w.r.t. a realization α of $\mathbb{X}^{(k)}(v)$, denoted by $\mathcal{L}^{(k)}_{\mathsf{out}}(v, \alpha; T, \Theta)$, is the joint probability of $\mathbb{X}^{(k)}(v) = \alpha$ and $\tilde{\mathbb{D}}^{(k)}_v = \tilde{\boldsymbol{D}}^{(k)}_u$. In other words:*

$$\mathcal{L}^{(k)}_{\mathsf{out}}(v, \alpha; T, \Theta) \coloneqq \mathcal{P}(\tilde{\mathbb{D}}^{(k)}_u = \tilde{\boldsymbol{D}}^{(k)}_v, \mathbb{X}^{(k)}(v) = \alpha; T, \mathbf{\Theta}) \tag{S5}$$

The outside likelihood can be computed using the following topdown recurrence:

$$\mathcal{L}^{(k)}_{\mathsf{out}}(v, \alpha_v; T, \Theta) = \begin{cases} 1, \text{ if } v = r_T \text{ and } \alpha_v = 0 \\ 0, \text{ if } v = r_T \text{ and } \alpha_v \neq 0 \\ \mathbf{\Psi}^{(k)}_{(u,v)}(0, \alpha_v), \text{ if } u \text{ is the parent of } v \text{ and } u = r_T \\ \sum_{\alpha_u} \mathbf{\Psi}^{(k)}_{(u,v)}(\alpha_u, \alpha_v)\mathcal{L}^{(k)}_{\mathsf{edge}}((u, w), \alpha_u; T, \Theta)\mathcal{L}^{(k)}_{\mathsf{out}}(u, \alpha_u; T, \mathbf{\Theta}), \text{ otherwise} \end{cases} \tag{S6}$$

where in the last case, u and w are the parent and sister of v, respectively. In other words, one can compute the outside likelihood of any node from the outside likelihood of its parent and the inside likelihoods of the sister branch.

S2.3.3 The posterior

Next, we define the *posterior probability of a node* and *posterior probability of an edge*.

Definition 4. *The posterior probability of a node u at site k w.r.t. the realization α_u of $\mathbb{X}^{(k)}(u)$, denoted by $\mathcal{S}^{(k)}_{\mathsf{node}}(u, \alpha_u; T, \Theta)$, is the probability of $\mathbb{X}^{(k)}(u) = \alpha_u$ given $\mathbb{D}^{(k)} = \boldsymbol{D}^{(k)}$. In other words:*

$$\mathcal{S}^{(k)}_{\mathsf{node}}(u, \alpha_u; T, \mathbf{\Theta}) \coloneqq \mathcal{P}(\mathbb{X}^{(k)}(u) = \alpha_u | \mathbb{D}^{(k)} = \boldsymbol{D}^{(k)}; T, \mathbf{\Theta}) \tag{S7}$$

The posterior probability of a node can be computed using the inside and outside likelihoods, as follows:

$$\mathcal{S}^{(k)}_{\mathsf{node}}(u, \alpha_u; T, \mathbf{\Theta}) = \begin{cases} 1, \text{ if } u \text{ is the root and } \alpha_u = 0 \\ 0, \text{ if } u \text{ is the root and } \alpha_u \neq 0 \\ \frac{\mathcal{L}^{(k)}_{\mathsf{node}}(u, \alpha_u; T, \mathbf{\Theta})\mathcal{L}^{(k)}_{\mathsf{out}}(u, \alpha_u; T, \mathbf{\Theta})}{\mathcal{P}(\mathbf{D}^{(k)}; T, \mathbf{\Theta})}, \text{ otherwise} \end{cases} \tag{S8}$$

Definition 5. *The posterior probability of an edge $e = (u, v)$ at site k w.r.t. the realization α_u of $\mathbb{X}^{(k)}(u)$ and α_v of $\mathbb{X}^{(k)}(v)$, denoted by $\mathcal{S}^{(k)}_{\text{edge}}(u, v, \alpha_u, \alpha_v; T, \Theta)$, is the joint probability of $\mathbb{X}^{(k)}(u) = \alpha_u$ and $\mathbb{X}^{(k)}(v) = \alpha_v$ given $\mathbb{D}^{(k)} = \boldsymbol{D}^{(k)}$. In other words:*

$$\mathcal{S}^{(k)}_{\text{edge}}(e, \alpha_u, \alpha_v; T, \boldsymbol{\Theta}) \coloneqq \mathcal{P}(\mathbb{X}^{(k)}(u) = \alpha_u, \mathbb{X}^{(k)}(v) = \alpha_v | \mathbb{D}^{(k)} = \boldsymbol{D}^{(k)}; T, \boldsymbol{\Theta}) \tag{S9}$$

The posterior probability of an edge $e = (u, v)$ can be computed from the other previously defined entities, as follows:

$$\mathcal{S}^{(k)}_{\text{edge}}(e, \alpha_u, \alpha_v; T, \boldsymbol{\Theta}) = \frac{\mathcal{S}^{(k)}_{\text{node}}(u, \alpha_u; T, \boldsymbol{\Theta})\mathcal{L}^{(k)}_{\text{node}}(v, \alpha_v; T, \boldsymbol{\Theta})\boldsymbol{\Psi}^{(k)}_e(\alpha_u, \alpha_v)}{\mathcal{L}^{(k)}_{\text{edge}}(e, \alpha_u; T, \boldsymbol{\Theta})} \tag{S10}$$

Thus, if all the inside likelihoods, outside likelihoods, and the full likelihood have been computed, we can use Eq. S10 and Eq. S8 to compute all the posterior assignments of states to nodes, in linear-time. With this observation, we can extend the Felsenstein's pruning algorithm to compute all the inside, outside likelihoods, and posterior assignments, as follows: step (1) compute and store the inside likelihoods at all nodes and branches using Eq. S2 and Eq. S4, in a bottom up tree traversal; step; step (2) compute and store the outside likelihoods at all nodes using Eq. S6, in a topdown tree traversal; step (3) compute the posterior assignments at all nodes using Eq. S10 and Eq. S8, in a tree traversal (either bottom-up or topdown). These values are needed for the E-step of the EM algorithm described in the main text.

S2.4 Derivation of the EM algorithm (Proof of Eq. 14 in the main text)

The basic setup of an EM algorithm to optimize $\mathcal{P}(\mathbb{D} = \mathbf{D}; \boldsymbol{\Theta})$ is as follows: in the E-step, compute for every site k the posterior probabilities $\mathcal{P}(\mathbb{X}^{(k)} = \mathbf{x}|\mathbb{D}^{(k)} = \mathbf{D}^{(k)}; \boldsymbol{\Theta}^t)$ for all possible realizations $\mathbf{x}$ of $\mathbb{X}^{(k)}$. In the M-step, compute $\boldsymbol{\Theta}^{t+1}$ as

$$\begin{aligned}
\boldsymbol{\Theta}^{t+1} &= \operatorname*{argmax}_{\boldsymbol{\Theta}} \sum_{k=1}^{K} \sum_{\mathbf{x}} \mathcal{P}(\mathbb{X}^{(k)} = \mathbf{x}|\mathbf{D}^{(k)}; \boldsymbol{\Theta}^t) \log \mathcal{P}(\mathbb{D}^{(k)} = \mathbf{D}^{(k)}, \mathbb{X}^{(k)} = \mathbf{x}; \boldsymbol{\Theta}) \\
&= \operatorname*{argmax}_{\boldsymbol{\Theta}} \sum_{k=1}^{K} \sum_{\mathbf{x}} \mathcal{P}(\mathbb{X}^{(k)} = \mathbf{x}|\mathbf{D}^{(k)}; \boldsymbol{\Theta}^t) \log \left[\prod_{e=(u,v)\in\mathscr{E}_T} \boldsymbol{\Psi}^{(k)}_e(\mathbf{x}(u), \mathbf{x}(v)) \prod_{w\in\mathscr{L}_{\mathscr{T}}} \boldsymbol{\Phi}(\mathbf{x}(w), \mathbf{D}^{(k)}(w)) \right] \\
&= \operatorname*{argmax}_{\boldsymbol{\Theta}} \sum_{k=1}^{K} \sum_{\mathbf{x}} \mathcal{P}(\mathbb{X}^{(k)} = \mathbf{x}|\mathbf{D}^{(k)}; \boldsymbol{\Theta}^t) \left[\sum_{e=(u,v)\in\mathscr{E}_T} \log \boldsymbol{\Psi}^{(k)}_e(\mathbf{x}(u), \mathbf{x}(v)) + \sum_{w\in\mathscr{L}_{\mathscr{T}}} \log \boldsymbol{\Phi}(\mathbf{x}(w), \mathbf{D}^{(k)}(w)) \right]
\end{aligned}$$

Note that every non-zero entries of $\boldsymbol{\Phi}$ can only be ϕ, $1 - \phi$, or 1. Therefore,

$$
\begin{aligned}
&\sum_{k=1}^{K}\sum_{\mathbf{x}}\mathcal{P}(\mathbb{X}^{(k)}=\mathbf{x}|\mathbf{D}^{(k)};\boldsymbol{\Theta}^{t})\sum_{w\in\mathscr{L}_{\mathscr{T}}}\log\boldsymbol{\Phi}(\mathbf{x}(w),\mathbf{D}^{(k)}(w))\\
&=\sum_{k=1}^{K}\sum_{\mathbf{x}}\sum_{w\in\mathscr{L}_{\mathscr{T}}}\mathcal{P}(\mathbb{X}^{(k)}=\mathbf{x}|\mathbf{D}^{(k)};\boldsymbol{\Theta}^{t})\log\boldsymbol{\Phi}(\mathbf{x}(w),\mathbf{D}^{(k)}(w))\\
&=\sum_{w\in\mathscr{L}_{T}}\sum_{\mathbf{x}:\mathbf{x}(w)\neq-1}\mathcal{P}(\mathbb{X}^{(k)}=\mathbf{x}|\mathbf{D}^{(k)};\boldsymbol{\Theta}^{t})\sum_{k:\mathbf{D}^{(k)}(w)=?}\log\phi+\sum_{w\in\mathscr{L}_{T}}\sum_{k:\mathbf{D}^{(k)}(w)\neq?}\log(1-\phi)\sum_{\mathbf{x}}\mathcal{P}(\mathbb{X}^{(k)}=\mathbf{x}|\mathbf{D}^{(k)};\boldsymbol{\Theta}^{t})\\
&=\sum_{w\in\mathscr{L}_{T}}\mathcal{P}(\mathbb{X}^{(k)}(w)\neq-1|\mathbf{D}^{(k)};\boldsymbol{\Theta}^{t})\log\phi+\sum_{w\in\mathscr{L}_{T}}\sum_{k:\mathbf{D}^{(k)}(w)\neq?}\log(1-\phi)\\
&=\sum_{w\in\mathscr{L}_{T}}(1-\mathcal{P}(\mathbb{X}^{(k)}(w)=-1|\mathbf{D}^{(k)};\boldsymbol{\Theta}^{t}))\log\phi+\sum_{w\in\mathscr{L}_{T}}|\{k:\mathbf{D}^{(k)}(w)\neq?\}|\log(1-\phi)\\
&=\sum_{w\in\mathscr{L}_{T}}(\tilde{\mathcal{B}}_{w}\log\phi+\mathcal{B}_{w}\log(1-\phi))
\end{aligned}
$$

The derivation of the other term is similar: note that all Ψ_e share the same locations of non-zero entries, and $\log\boldsymbol{\Psi}_e^{(k)}(\mathbf{x}(u),\mathbf{x}(v))$ can only be $-\delta_e(1+\nu)$, $\log 1-e^{-\delta_e}$, $-\nu\delta_e$, $-\delta\nu(1-e^{-\delta_e})$, or 0 (plus constants). Therefore, we have (skipping details, as the derivation is very similar to [59]):

$$
\begin{aligned}
&\sum_{k=1}^{K}\sum_{\mathbf{x}}\mathcal{P}(\mathbb{X}^{(k)}=\mathbf{x}|\mathbf{D}^{(k)};\boldsymbol{\Theta}^{t})\sum_{e=(u,v)\in\mathscr{E}_{T}}\log\boldsymbol{\Psi}_e^{(k)}(\mathbf{x}(u),\mathbf{x}(v))\\
&=\sum_{e\in\mathscr{E}_{T}}\Big(-\mathcal{C}_e^{z\to z}(1+\nu)\delta_e+\mathcal{C}_e^{z\to a}(\log(1-e^{-\delta_e})-\nu\delta_e)+\mathcal{C}_e^{z\to m}\log(1-e^{-\delta_e\nu})-\mathcal{C}_e^{a\to a}\delta_e\nu+\mathcal{C}_e^{a\to m}\log(1-e^{-}
\end{aligned}
$$

Adding the two terms, we get Eq. 14 in the main text.

S2.5 Reducing the complexity of the E-step

In the E-step, we need to compute $\mathcal{C}_e^{z\to z}, \mathcal{C}_e^{z\to a}, \mathcal{C}_e^{z\to m}, \mathcal{C}_e^{a\to a}$, and $\mathcal{C}_e^{a\to m}$ for all branches e. Below we introduce an algorithm to compute them in $O(NK)$, where N and K are the number of cells and target sites, respectively. Importantly, the complexity of our algorithm is independent of the alphabet size of any target site. First, we introduce the concepts of **α-trees,** **α-clades**, and **z-branches** that will be useful in describing the algorithm.

Definition 6. *Consider a target site k of the lineage tracing data **D** whose alphabet is $\mathscr{A}^{(k)}$ and a character state $\alpha \in \mathscr{A}^{(k)} \setminus \{0, -1\}$. An **$\alpha$-tree** on site k at state α of **D** is a tree T with leafset $\mathscr{L}_T$ such that for all $w \in \mathscr{L}_T$, $\boldsymbol{D}^{(k)}(w) \in \{?, \alpha\}$.*

Definition 7. *An **α-clade** of a tree T with respect to a site k of a lineage tracing data **D** is a clade of T that forms an α-tree on site k at state α of **D**.*

Note that the definitions of α-tree and α-clade include the case where $\alpha =?$ (i.e. all leaves of T have state "?"). In such a case, we call the tree/clade a *masked tree/clade*.

Definition 8. *A **masked-clade** of a tree T with respect to a site k of lineage tracing data **D** is an α-clade of T at k where $\alpha = ?$. Any branch belongs to a masked-clade is called a **masked branch**. Any branch that is not a masked branch is referred to as an **unmasked** branch.*

Note that an α-tree where $\alpha \neq ?$ may include some masked clades and masked branches inside it.

Definition 9. *A **z-branch** of a tree T with respect to a site k of a lineage tracing data **D** is a branch that does not belong to any α-clade of T on k, for all $\alpha \in \mathscr{A}^{(k)} \setminus \{0, -1\}$.*

S2.5.1 Special properties of α-clades and z-branches

Theorem 1. *[az-partition] Consider a target site k of the lineage tracing data **D** whose alphabet is $\mathscr{A}^{(k)}$. Any tree T on $\boldsymbol{D}^{(k)}$ can be partitioned into **edge-disjoint** α-clades (each can have a different $\alpha \in \mathscr{A}^{(k)} \setminus \{0, -1\}$) and z-branches. This partition is unique and is named the **az-partition** of T with respect to $\boldsymbol{D}^{(k)}$.*

Proof. The following is an algorithm to partition any tree T into α-clades and z-branches.

Algorithm 3 Linear-time algorithm to perform az-partition. Input: a tree topology T and a target-site $\mathbf{D}^{(k)}$. Output: the az-partition of T with respect to $\mathbf{D}^{(k)}$.

```
1:  function AZPARTITION(T, D^(k))
2:      alphaTrees, zBranches ← [], []
3:      for u in postorder traversal of T do
4:          if u is a leaf then                         ▷ Set tag by the state of the leaf node.
5:              if D^(k)(u) == 0 then
6:                  u.tag ← "z"
7:                  Add the branch incident to u to zBranches
8:              else
9:                  u.tag ← D^(k)(u)
10:             end if
11:         else                                        ▷ Set tag by the tags of the child nodes
12:             a, b ← children(u)
13:             if (a.tag == b.tag and a ≠ "z") or (one is "?") then
14:                 u.tag ← a.tag if a.tag != "?" else b.tag
15:             else
16:                 u.tag ←"z"                          ▷ the branch above u is a z-branch.
17:             end if
18:             if u.tag =="z" then
19:                 Add the branch incident to u to zBranches
20:                 if a.tag is not "z" then            ▷ u has tag "z" and a does not
21:                     Add the clade attached to branch (u, a) to alphaTrees
22:                 end if
23:                 if b.tag is not "z" then            ▷ u has tag "z" and b does not
24:                     Add the clade attached to branch (u, b) to alphaTrees
25:                 end if
26:             end if
27:         end if
28:     end for
29:     return alphaTrees, zBranches
30: end function
```

We will analyze this algorithm to demonstrate that it produces a unique az-partition of T with respect to $D^{(i)}$.

The algorithm iterates over every node u in the tree T, and considers the incident branch, so that the algorithm considers every branch e. A branch e is either tagged and output as a zBranch (lines 6-7, 15-17), or tagged with the leaf state (lines 12-13). If the branch is tagged with a leaf state α, then it must necessarily be output as part of the corresponding α-tree at a later iteration. Since a zBranch marks the first branch where not all leaves share the same state, the branches above a zBranch cannot lie in an alphaTree, so alphaTrees must be pairwise disjoint. Therefore, each branch must be output either as part of an alphaTree or as a zBranch, and will only appear once in the output. Thus, we have that the algorithm produces a valid partition.

We should next show that alphaTrees are indeed α-clades of T for some α. For any alphaTree T_α returned by the algorithm, we know that by construction all branches in that alphaTree are tagged with either α or "?", except for the root branch, which is tagged with a z. For a branch to be tagged with a leaf state α, the incident node u's children have tags in the set $\{\alpha, ?\}$, recursively down to the leaves (line 11-13) such that all the leaf nodes under this branch must have state in the set $\{\alpha, ?\}$. Lines 19 and 21, where the entire set of branches below a z-branch is returned, determine that the alphaTrees returned must be a clade. Therefore, by construction we know that the returned trees must be clades that each form an alphaTree with corresponding leafset $\in \{\alpha, ?\}$, as stipulated by Def. 6 and 7.

We should next show that returned zBranches fit Def. 9. zBranches are returned in a few cases: on line 5-6, a zBranch could be returned if the leaf state is 0, which is consistent with Def 9. The other case when a zBranch is returned happens in lines 16-17, where if a node u is tagged with a z, then the incident branch b will be returned as a zBranch. A few scenarios force a node u to be tagged with z: (1) its two children do not share the same tag, (2) two children are both tagged with z. In Case (1), the leaves under branch b do not share states $\in \{\alpha, ?\}$, and so this branch cannot belong to any α-clade and must necessarily be a zBranch. In Case (2), if both children are tagged with z, then the leaves under each child node respectively do not share states $\in \{\alpha, ?\} \forall \alpha \in \Sigma_i \setminus \{0, -1\}$, so that this branch cannot belong to any α-clade and must also necessarily be a zBranch. Therefore, all elements of the returned zBranches must be a zBranch, and since all branches are represented at least once, and all alphaTree branches are accounted for, the algorithm must have returned all zBranches.

Putting the above statements together, we want to show that if the algorithm always returns an answer, then the partition must exist. Suppose towards a contradiction the algorithm returns a set of alphaTrees and zBranches, but that no partition exists. However, we saw above that the algorithm necessarily outputs all branches, and each branch appears once as either a zBranch, or as a branch in an alphaTree. Therefore, we must have a valid partition, but we have assumed that no partition exists, and we arrive at a contradiction. We can see that the algorithm must always return an answer given a valid input, since the algorithm output is an assignment for each branch. Consequently, it must be true that if the algorithm returns an answer, then the partition must exist.

Finally, it remains to be shown that the partition is unique. Suppose towards a contradiction that we have two partitions P_1 and P_2 which are both valid partitions for an input tree T and data $\mathbf{D}^{(k)}$, but which are different without loss of generality by one branch b. Since all branches must show up in both valid partitions, this different branch b could be different in tow ways: Case (1): $\in$ the set of zBranches in P_1 and $\in$ an alphaTree in P_2, or Case (2): $\in$ topology alphaTree T_α in P_1 and $\notin$ topology alphaTree T_β in P_2.

In Case (1), suppose towards a contradiction that it is true a branch b could be a zBranch in one valid partition, and in an alphaTree in another valid partition. Then, in valid partition P_1, the leaf states below b must not lie in the set $\{\alpha, ?\}$, and in valid partition P_2, the leaf states do lie in the set $\{\alpha ?\}$. However, both cannot be true, since we have the same tree topology T and data $\mathbf{D}^{(k)}$. Therefore, we have arrived at a contradiction, and we know that any branch must either lie in a zBranch or in an alphaTree exclusively, in all valid partitions.

In the other possible Case (2), suppose towards a contradiction that b has been assigned to two different alphatrees; that is, b is assigned to $T_\alpha \in P_1$ and not assigned to $T_\beta \in P_2$, where T_α and T_β are topologically the same except for branch b. Then, all leaves under branch

$b \in P_1$ must necessarily lie in the allowed T_α leaf states. In partition P_2, for branch b to belong to a different alphaTree, it must be tagged with a different α. However, the input data is the same, and so all the leaves below branch b must have the same leaf state in both partitions. We have arrived at a contradiction, and thus we have that the partition returned by the algorithm must be unique.

Since missing clades are special cases of alphaTrees, we will not discuss them specially here, except to note that all branches in an alphaTree (masked branches included) will only be output as part of an alphaTree when the ancestral zBranch is processed. We should note that the branch incident to the case where one child has the missing state, and the other child has a nonmissing, nonzero state is not a zBranch.

Thus, we can see that the algorithm must necessarily provide a valid partition of our input tree and data into alphaTrees and a set of zBranches fitting the proposed definitions. □

Corollary 1. *Any branch $e = (u, v)$ of T is either a z-branch, a masked-branch, or an unmasked branch of some α_0-clade of T where $\alpha_0 \in \mathscr{A}^{(k)} \setminus \{?, 0, -1\}$, with respect to $\boldsymbol{D}^{(k)}$. In addition, if e is not a z-branch or masked-branch, it has a unique associated α_0.*

Theorem 2. *[Properties of a z-branch] Given a tree topology T parameterized by Θ, a target site k, and a z-branch $e = (u, v)$ of T on site k. We have the following statements:*

$$
\begin{aligned}
&(1)\mathcal{L}^{(k)}_{\text{node}}(v, \alpha; T, \boldsymbol{\Theta}) = \mathcal{L}^{(k)}_{\text{edge}}(e, \alpha; T, \boldsymbol{\Theta}) = 0,\ \forall \alpha \neq 0 \\
&(2)\mathcal{S}^{(k)}_{\text{edge}}(e, \alpha, \beta; T, \boldsymbol{\Theta}) = 0,\ \forall (\alpha, \beta) \neq (0, 0) \\
&(3)\mathcal{S}^{(k)}_{\text{edge}}(e, 0, 0; T, \boldsymbol{\Theta}) = 1 \\
&(4)\mathcal{S}^{(k)}_{\text{node}}(u, 0; T, \boldsymbol{\Theta}) = 1 \\
&(5)\mathcal{S}^{(k)}_{\text{node}}(u, \alpha; T, \boldsymbol{\Theta}) = 0,\ \forall \alpha \neq 0
\end{aligned}
$$

Proof. **Proof of Statement (1)**. To prove (1), first we prove that if e is a z-branch of site k, then leaves in $\mathbf{D}^{(k)}_v$ belong to two distinct unmasked states. Suppose towards a contradiction that all leaves in $\mathbf{D}^{(k)}_v$ are either in masked state "?" or a same state α_0; by definition of α-clade, $\mathbf{D}^{(k)}_v$ defines an α-clade on T where $\alpha = \alpha_0$ or $\alpha =?$. By definition, e belongs to the clade defined by $\mathbf{D}^{(k)}_v$, so it is part of an α-clade, contradicting the assumption that e is a z-branch.

Hence, there exists two leaves l_1 and l_2 under v such that $\mathbf{D}^{(k)}(l_1) = \beta_1 \neq \mathbf{D}^{(k)}(l_2) = \beta_2$, where $\beta_1, \beta_2 \in \mathscr{A}^{(k)} \setminus \{?\}$. Let w be the LCA of l_1 and l_2, so $\mathbb{D}^{(k)}(l_1)$ and $\mathbb{D}^{(k)}(l_2)$ are conditionally independent given $\mathbb{X}^{(k)}(w)$). Recall that $\mathbf{D}^{(k)}_v$ includes $\mathbf{D}^{(k)}(l_1)$ and $\mathbf{D}^{(k)}(l_2)$.

For any $\alpha \neq 0$, we have:

$$\begin{aligned}
0 &\leq \mathcal{L}^{(k)}_{\textsf{node}}(v, \alpha; T, \boldsymbol{\Theta}) = \mathcal{P}(\mathbb{D}^{(k)}_v = \mathbf{D}^{(k)}_v | \mathbb{X}^{(k)}(v) = \alpha) \\
&\leq \mathcal{P}(\mathbb{D}^{(k)}(l_1) = \mathbf{D}^{(k)}(l_1), \mathbb{D}^{(k)}(l_2) = \mathbf{D}^{(k)}(l_2) | \mathbb{X}^{(k)}(v) = \alpha) \\
&= \mathcal{P}(\mathbb{D}^{(k)}(l_1) = \beta_1, \mathbb{D}^{(k)}(l_2) = \beta_2 | \mathbb{X}^{(k)}(v) = \alpha) \\
&= \sum_{\beta \in \mathscr{A}^{(k)}} \mathcal{P}(\mathbb{D}^{(k)}(l_1) = \beta_1, \mathbb{D}^{(k)}(l_2) = \beta_2 | \mathbb{X}^{(k)}(w) = \beta) \mathcal{P}(\mathbb{X}^{(k)}(w) = \beta | \mathbb{X}^{(k)}(v) = \alpha) \\
&= \sum_{\beta \in \mathscr{A}^{(k)}} \mathcal{P}(\mathbb{D}^{(k)}(l_1) = \beta_1 | \mathbb{X}^{(k)}(w) = \beta) \mathcal{P}(\mathbb{D}^{(k)}(l_2) = \beta_2 | \mathbb{X}^{(k)}(w) = \beta) \mathcal{P}(\mathbb{X}^{(k)}(w) = \beta | \mathbb{X}^{(k)}(v) = \alpha) \\
&= 0,
\end{aligned}$$

where the last equation holds because all terms inside the last summation are 0: if $\beta = 0$ or $\beta \notin \{\alpha, -1\}$, then $\mathcal{P}(\mathbb{X}^{(k)}(w) = \beta | \mathbb{X}^{(k)}(v) = \alpha) = 0$; else if $\beta = -1$ then $\mathcal{P}(\mathbf{D}^{(k)}(l_1) = \beta_1 | \mathbb{X}^{(k)}(w) = \beta) = \mathcal{P}(\mathbf{D}^{(k)}(l_2) = \beta_2 | \mathbb{X}^{(k)}(w) = \beta) = 0$ because $\beta_1 \neq ?$ and $\beta_2 \neq ?$; otherwise, $\beta = \alpha$, so either $\mathcal{P}(\mathbf{D}^{(k)}(l_1) = \beta_1 | \mathbb{X}^{(k)}(w) = \beta) = 0$ or $\mathcal{P}(\mathbf{D}^{(k)}(l_2) = \beta_2 | \mathbb{X}^{(k)}(w) = \beta) = 0$ because $\beta_1 \neq \beta_2$ and $\alpha \neq 0$. Thus, $\mathcal{P}(\mathbf{D}^{(k)}_v | \mathbb{X}^{(k)}(v) = \alpha) = 0$ for all $\alpha \neq 0$. Now we prove the second equality of (1). For any $\alpha \neq 0$, we have:

$$\mathcal{P}(\mathbf{D}^{(k)}_v | \mathbb{X}^{(k)}(u) = \alpha; T, \Theta) = \sum_{\beta \in \mathscr{A}^{(k)}} \mathcal{P}(\mathbf{D}^{(k)}_v | \mathbb{X}^{(k)}(v) = \beta; T, \Theta) \mathcal{P}(\mathbb{X}^{(k)}(v) = \beta | \mathbb{X}^{(k)}(u) = \alpha; T, \Theta) = 0,$$

where the second equality holds because $\mathcal{P}(\mathbb{X}^{(k)}(v) = 0 | \mathbb{X}^{(k)}(u) = \alpha; T, \Theta) = 0$ (recall that $\alpha \neq 0$) and $\mathcal{P}(\mathbf{D}^{(k)}_v | \mathbb{X}^{(k)}(v) = \beta; T, \Theta) = 0$ for all $\beta \neq 0$.

Proof of Statement (2). Using Eq. S10, we have:

$$\mathcal{S}^{(k)}_{\textsf{edge}}(e, \alpha, \beta; T, \boldsymbol{\Theta}) = \frac{\mathcal{S}^{(k)}_{\textsf{node}}(u, \alpha; T, \boldsymbol{\Theta}) \mathcal{L}^{(k)}_{\textsf{node}}(v, \beta; T, \boldsymbol{\Theta}) \boldsymbol{\Psi}^{(k)}_e(\alpha, \beta)}{\mathcal{L}^{(k)}_{\textsf{edge}}(e, \alpha; T, \boldsymbol{\Theta})}$$

Recall that $(\alpha, \beta) \neq (0, 0)$, so either $\beta \neq 0$ or ($\beta = 0$ and $\alpha \neq 0$).

- Case 1: $\beta \neq 0$. In this case, $\mathcal{L}^{(k)}_{\textsf{node}}(v, \beta; T, \boldsymbol{\Theta}) = 0$ (Statement (1)), so $\mathcal{S}^{(k)}_{\textsf{edge}}(e, \alpha, \beta; T, \Theta) = 0$.
- Case 2: $\beta = 0$ and $\alpha \neq 0$. In this case, $\boldsymbol{\Psi}^{(k)}_e(\alpha, \beta) = 0$ (see Eq. 4 in the main text), so $\mathcal{S}^{(k)}_{\textsf{edge}}(e, \alpha, \beta; T, \Theta) = 0$.

Proof of Statement (3). It is straightforward to derive Statement (3) from (2):

$$\begin{aligned}
\mathcal{S}^{(k)}_{\text{edge}}(e,0,0;T,\boldsymbol{\Theta}) &= \mathcal{P}(\mathbb{X}^{(k)}(u)=0,\mathbb{X}^{(k)}(v)=0|\mathbf{D}^{(k)};T,\Theta)\\
&= 1-\sum_{(\alpha,\beta)\neq(0,0)}\mathcal{P}(\mathbb{X}^{(k)}(u)=\alpha,\mathbb{X}^{(k)}(v)=\beta|\mathbf{D}^{(k)};T,\Theta)\\
&= 1-\sum_{(\alpha,\beta)\neq(0,0)}\mathcal{S}^{(k)}_{\text{edge}}(e,\alpha,\beta;T,\boldsymbol{\Theta})\\
&= 1
\end{aligned}$$

Proof of Statement (4). We prove Statement (4) using Statements (2) and (3):

$$\begin{aligned}
\mathcal{S}^{(k)}_{\text{node}}(u,0;T,\boldsymbol{\Theta}) &= \mathcal{P}(\mathbb{X}^{(k)}(u)=0|\mathbf{D}^{(k)};T,\Theta)\\
&= \textstyle\sum_{\beta}\mathcal{P}(\mathbb{X}^{(k)}(u)=0,\mathbb{X}^{(k)}(v)=\beta|\mathbf{D}^{(k)};T,\Theta)\\
&= \textstyle\sum_{\beta\neq 0}\mathcal{P}(\mathbb{X}^{(k)}(u)=0,\mathbb{X}^{(k)}(v)=\beta|\mathbf{D}^{(k)};T,\Theta)+\mathcal{P}(\mathbb{X}^{(k)}(u)=0,\mathbb{X}^{(k)}(v)=0|\mathbf{D}^{(k)};T,\Theta)\\
&= \textstyle\sum_{\beta\neq 0}\mathcal{S}^{(k)}_{\text{edge}}(e,0,\beta;T,\boldsymbol{\Theta})+\mathcal{S}^{(k)}_{\text{edge}}(e,0,0;T,\boldsymbol{\Theta})=1
\end{aligned}$$

The last equality is justified by Statements (2) and (3).

Proof of Statement (5). For all $\alpha\neq 0$, we have:

$$\begin{aligned}
0\leq\mathcal{S}^{(k)}_{\text{node}}(u,\alpha;T,\boldsymbol{\Theta}) &\leq \sum_{\beta\neq 0}\mathcal{S}^{(k)}_{\text{node}}(u,\beta;T,\boldsymbol{\Theta})\\
&= \sum_{\beta\neq 0}\mathcal{P}(\mathbb{X}^{(k)}(u)=\beta|\mathbf{D}^{(k)};T,\Theta)\\
&= 1-\mathcal{P}(\mathbb{X}^{(k)}(u)=0|\mathbf{D}^{(k)};T,\Theta)\\
&= 1-\mathcal{S}^{(k)}_{\text{node}}(u,0;T,\boldsymbol{\Theta})=0
\end{aligned}$$

The last equality is justified by Statement (4). Thus, $\mathcal{S}^{(k)}_{\text{node}}(u,\alpha;T,\boldsymbol{\Theta})=0$ for all $\alpha\neq 0$. □

Theorem 3. *[Properties of a masked-branch] Consider a masked clade of a tree T at a site k of **D** and $e=(u,v)$ is an arbitrary branch of this masked clade. Then we have*

(1) $\mathcal{L}^{(k)}_{\text{node}}(v,-1;T,\boldsymbol{\Theta})=\mathcal{L}^{(k)}_{\text{edge}}(e,-1;T,\boldsymbol{\Theta})=1$

(2) $\mathcal{L}^{(k)}_{\text{edge}}(e,\alpha;T,\boldsymbol{\Theta})=\mathcal{L}^{(k)}_{\text{edge}}(e,\beta;T,\boldsymbol{\Theta}),\forall\alpha,\beta\in\mathscr{A}^{(k)}\setminus\{0,-1,?\},\alpha\neq\beta$

(3) $\mathcal{L}^{(k)}_{\text{node}}(v,\alpha;T,\boldsymbol{\Theta})=\mathcal{L}^{(k)}_{\text{node}}(v,\beta;T,\boldsymbol{\Theta}),\forall\alpha,\beta\in\mathscr{A}^{(k)}\setminus\{0,-1,?\},\alpha\neq\beta$

Proof. **Proof of (1)**. Let Clade(v) denote the leaf set of the clade of T below v. We have:

$$\begin{aligned}
&1\geq\mathcal{P}(\mathbf{D}^{(k)}_v|\mathbb{X}^{(k)}(v)=-1;T,\Theta)\geq\prod_{l\in\text{Clade}(v)}\mathcal{P}(\mathbf{D}^{(k)}(l)|\mathbb{X}^{(k)}(v)=-1;T,\Theta)=1\\
&\implies\mathcal{P}(\mathbf{D}^{(k)}_v|\mathbb{X}^{(k)}(v)=-1;T,\Theta)=1\\
&\implies\mathcal{L}^{(k)}_{\text{node}}(v,-1;T,\boldsymbol{\Theta})=1
\end{aligned}$$

Now we prove the second equality of Statement (1). Indeed:

$$\begin{aligned}\mathcal{L}^{(k)}_{\mathsf{edge}}(e,-1;T,\mathbf{\Theta}) &= \mathcal{P}(\mathbf{D}^{(k)}_v|\mathbb{X}^{(k)}(u)=-1;T,\Theta)\\ &= \textstyle\sum_{\alpha\in\mathscr{A}^{(k)}} \mathcal{P}(\mathbf{D}^{(k)}_v|\mathbb{X}^{(k)}(v)=\alpha;T,\mathbf{\Theta})\mathcal{P}(\mathbb{X}^{(k)}(v)=\alpha|\mathbb{X}^{(k)}(u)=-1;T,\Theta)\\ &= \textstyle\sum_{\alpha\in\mathscr{A}^{(k)}} \mathcal{L}^{(k)}_{\mathsf{node}}(v,\alpha;T,\mathbf{\Theta})\mathbf{\Psi}_e(-1,\alpha)\\ &= \textstyle\sum_{\alpha\neq 0} \mathcal{L}^{(k)}_{\mathsf{node}}(v,\alpha;T,\mathbf{\Theta})\mathbf{\Psi}_e(-1,\alpha)+\mathcal{L}^{(k)}_{\mathsf{node}}(v,-1;T,\mathbf{\Theta})\mathbf{\Psi}_e(-1,-1)\\ &= \mathcal{L}^{(k)}_{\mathsf{node}}(v,-1;T,\mathbf{\Theta})\mathbf{\Psi}_e(-1,-1)=1\end{aligned}$$

Proof of (2). We will prove by induction. Base case: e is a terminal branch (i.e. v is a leaf node), so that $\mathbf{D}^{(k)}_v=\mathbf{D}^{(k)}(v)$. In addition, because e is masked, $\mathbf{D}^{(k)}(v)=?$. So, we have:

$$\begin{aligned}\mathcal{L}^{(k)}_{\mathsf{edge}}(e,\alpha;T,\mathbf{\Theta}) &= \mathcal{P}(\mathbb{D}^{(k)}_v=\mathbf{D}^{(k)}_v|\mathbb{X}^{(k)}(u)=\alpha)\\ &= \mathcal{P}(\mathbb{D}^{(k)}(v)=?|\mathbb{X}^{(k)}(u)=\alpha)\\ &= \textstyle\sum_{\gamma\in\mathscr{A}^{(k)}} \mathcal{P}(\mathbb{D}^{(k)}(v)=?|\mathbb{X}^{(k)}(v)=\gamma)\mathcal{P}(\mathbb{X}^{(k)}(v)=\gamma|\mathbb{X}^{(k)}(u)=\alpha)\\ &= \textstyle\sum_{\gamma\in\mathscr{A}^{(k)}} \mathbf{\Phi}(\gamma,?)\mathbf{\Psi}^{(k)}(\alpha,\gamma)\\ &= \phi p^{\nu}_e+(1-p^{\nu}_e)\end{aligned}$$

Similarly, we can prove that $\mathcal{L}^{(k)}_{\mathsf{edge}}(e,\beta;T,\mathbf{\Theta})=\phi p^{\nu}_e+(1-p^{\nu}_e)$, so $\mathcal{L}^{(k)}_{\mathsf{edge}}(e,\alpha;T,\mathbf{\Theta})=\mathcal{L}^{(k)}_{\mathsf{edge}}(e,\beta;T,\mathbf{\Theta})$.

Now we prove the induction case. If e is not a terminal branch, then v has two children v_1,v_2. Suppose that the statement is correct for the two branches $e_1=(v,v_1)$ and $e_2=(v,v_2)$, we will prove that it is also correct for branch $e=(u,v)$. For any state $\alpha\in\mathscr{A}^{(k)}\setminus\{0,-1,?\}$, we have:

$$\begin{aligned}\mathcal{L}^{(k)}_{\mathsf{edge}}(e,\alpha;T,\mathbf{\Theta}) &= \mathcal{P}(\mathbf{D}^{(k)}_v|\mathbb{X}^{(k)}(u)=\alpha)\\ &= \mathcal{P}(\mathbf{D}^{(k)}_{v_1},\mathbf{D}^{(k)}_{v_2}|\mathbb{X}^{(k)}(u)=\alpha)\\ &= \textstyle\sum_{\gamma\in\mathscr{A}^{(k)}} \mathcal{P}(\mathbf{D}^{(k)}_{v_1},\mathbf{D}^{(k)}_{v_2},\mathbb{X}^{(k)}(v)=\gamma|\mathbb{X}^{(k)}(u)=\alpha)\\ &= \textstyle\sum_{\gamma\in\mathscr{A}^{(k)}} \mathcal{P}(\mathbf{D}^{(k)}_{v_1}|\mathbb{X}^{(k)}(v)=\gamma)\mathcal{P}(\mathbf{D}^{(k)}_{v_2}|\mathbb{X}^{(k)}(v)=\gamma)\mathcal{P}(\mathbb{X}^{(k)}(v)=\gamma|\mathbb{X}^{(k)}(u)=\alpha)\\ &= \textstyle\sum_{\gamma\in\mathscr{A}^{(k)}} \mathcal{L}^{(k)}_{\mathsf{edge}}(e_1,\gamma;T,\mathbf{\Theta})\mathcal{L}^{(k)}_{\mathsf{edge}}(e_2,\gamma;T,\mathbf{\Theta})\mathbf{\Psi}_e(\alpha,\gamma)\end{aligned}$$

Recall that $\mathbf{\Psi}_e$ is a sparse matrix. Because $\alpha\neq 0$, $\mathbf{\Psi}_e(\alpha,\gamma)=0$ for all $\gamma\neq\{-1,\alpha\}$. Therefore, we have:

$$\begin{aligned}\mathcal{L}^{(k)}_{\mathsf{edge}}(e,\alpha;T,\mathbf{\Theta}) &= \mathcal{L}^{(k)}_{\mathsf{edge}}(e_1,-1;T,\mathbf{\Theta})\mathcal{L}^{(k)}_{\mathsf{edge}}(e_2,-1;T,\mathbf{\Theta})\mathbf{\Psi}_e(\alpha,-1)+\mathcal{L}^{(k)}_{\mathsf{edge}}(e_1,\alpha;T,\mathbf{\Theta})\mathcal{L}^{(k)}_{\mathsf{edge}}(e_2,\alpha;T,\mathbf{\Theta})\mathbf{\Psi}_e(\alpha,\alpha)\\ &= \mathcal{L}^{(k)}_{\mathsf{edge}}(e_1,-1;T,\mathbf{\Theta})\mathcal{L}^{(k)}_{\mathsf{edge}}(e_2,-1;T,\mathbf{\Theta})(1-p^{\nu}_e)+\mathcal{L}^{(k)}_{\mathsf{edge}}(e_1,\alpha;T,\mathbf{\Theta})\mathcal{L}^{(k)}_{\mathsf{edge}}(e_2,\alpha;T,\mathbf{\Theta})p^{\nu}_e\end{aligned}$$

By induction assumptions, for any state $\beta\in\mathscr{A}^{(k)}\setminus\{0,-1,?\}$ that is distinct from α, we have $\mathcal{L}^{(k)}_{\mathsf{edge}}(e_1,\alpha;T,\mathbf{\Theta})=\mathcal{L}^{(k)}_{\mathsf{edge}}(e_1,\beta;T,\mathbf{\Theta})$ and $\mathcal{L}^{(k)}_{\mathsf{edge}}(e_2,\alpha;T,\mathbf{\Theta})=\mathcal{L}^{(k)}_{\mathsf{edge}}(e_2,\beta;T,\mathbf{\Theta})$. Thus:

$$\begin{aligned}\mathcal{L}^{(k)}_{\mathsf{edge}}(e,\alpha;T,\mathbf{\Theta}) &= \mathcal{L}^{(k)}_{\mathsf{edge}}(e_1,-1;T,\mathbf{\Theta})\mathcal{L}^{(k)}_{\mathsf{edge}}(e_2,-1;T,\mathbf{\Theta})(1-p^{\nu}_e)+\mathcal{L}^{(k)}_{\mathsf{edge}}(e_1,\beta;T,\mathbf{\Theta})\mathcal{L}^{(k)}_{\mathsf{edge}}(e_2,\beta;T,\mathbf{\Theta})p^{\nu}_e\\ &= \mathcal{L}^{(k)}_{\mathsf{edge}}(e,\beta;T,\mathbf{\Theta})\end{aligned}$$

Proof of (3). Similar to (2), we can use induction to prove this statement. $\square$

Theorem 4. *[Properties of unmasked branch of an α-tree] Consider an α-clade of a tree T at a site k of $\mathbf{D}$ where $\alpha \neq ?$ and $e = (u, v)$ is an arbitrary unmasked branch of this clade. Then:*

$$(1)\ \mathcal{L}^{(k)}_{\text{node}}(v, \beta; T, \mathbf{\Theta}) = 0,\ \forall \beta \in \mathscr{A}^{(k)} \setminus \{0, \alpha\}$$
$$(2)\ \mathcal{L}^{(k)}_{\text{edge}}(e, \beta; T, \mathbf{\Theta}) = 0,\ \forall \beta \in \mathscr{A}^{(k)} \setminus \{0, \alpha\}$$
$$(3)\ \mathcal{S}^{(k)}_{\text{edge}}(\beta, \gamma; T, \mathbf{\Theta}) = 0,\ \forall (\beta, \gamma) \in (\mathscr{A}^{(k)})^2 \setminus \{(0,0), (0,\alpha), (\alpha,\alpha)\}$$

Proof. **Proof of (1)** Because $e = (u, v)$ is an unmasked branch of an α-clade of T on $\mathbf{D}^{(k)}$, by definition there exists at least one leaf node l of T under v that has $\mathbf{D}^{(k)}(l) = \alpha$ and $\mathbf{D}^{(k)}(l) \in \mathbf{D}^{(k)}_v$. Let w be the parent of l, and $e_1 = (w, l)$. It is easy to see that $\mathcal{P}(\mathbb{X}^{(k)}(l) = \gamma | \mathbb{X}^{(k)}(v) = \beta; T, \mathbf{\Theta}) \leq \mathcal{P}(\mathbb{X}^{(k)}(l) = \gamma | \mathbb{X}^{(k)}(w) = \beta; T, \mathbf{\Theta}) = \mathbf{\Psi}_{e_1}(\beta, \gamma)$ for all $\gamma \in \mathscr{A}^{(k)}$. So we have:

$$\begin{aligned}
0 &\leq \mathcal{L}^{(k)}_{\text{node}}(v, \beta; T, \mathbf{\Theta}) \\
&= \mathcal{P}(\mathbb{D}^{(k)}_v = \mathbf{D}^{(k)}_v | \mathbb{X}^{(k)}(v) = \beta; T, \mathbf{\Theta}) \\
&\leq \mathcal{P}(\mathbb{D}^{(k)}(l) = \mathbf{D}^{(k)}(l) | \mathbb{X}^{(k)}(v) = \beta; T, \mathbf{\Theta}) \\
&= \mathcal{P}(\mathbb{D}^{(k)}(l) = \alpha | \mathbb{X}^{(k)}(v) = \beta; T, \mathbf{\Theta}) \\
&= \sum_{\gamma} \mathcal{P}(\mathbb{D}^{(k)}(l) = \alpha, \mathbb{X}^{(k)}(l) = \gamma | \mathbb{X}^{(k)}(v) = \beta; T, \mathbf{\Theta}) \\
&= \sum_{\gamma} \mathcal{P}(\mathbb{D}^{(k)}(l) = \alpha | \mathbb{X}^{(k)}(l) = \gamma; T, \mathbf{\Theta}) \mathcal{P}(\mathbb{X}^{(k)}(l) = \gamma | \mathbb{X}^{(k)}(v) = \beta; T, \mathbf{\Theta}) \\
&\leq \sum_{\gamma} \mathbf{\Phi}(\gamma, \alpha) \mathbf{\Psi}_{e_1}(\beta, \gamma) = 0,
\end{aligned}$$

where the last equality holds because of the sparsity of $\mathbf{\Phi}$ and $\mathbf{\Psi}_{e_1}$ (note that $\beta \neq 0$ and $\beta \neq \alpha$).

Proof of (2) Similar to the proof for (1), observe that $\mathcal{P}(\mathbb{X}^{(k)}(l) = \gamma | \mathbb{X}^{(k)}(u) = \beta; T, \mathbf{\Theta}) \leq \mathcal{P}(\mathbb{X}^{(k)}(l) = \gamma | \mathbb{X}^{(k)}(w) = \beta; T, \mathbf{\Theta}) = \mathbf{\Psi}_{e_1}(\beta, \gamma)$ for all $\gamma \in \mathscr{A}^{(k)}$. So we have:

$$\begin{aligned}
0 &\leq \mathcal{L}^{(k)}_{\text{edge}}(e, \beta; T, \mathbf{\Theta}) \\
&= \mathcal{P}(\mathbb{D}^{(k)}_v = \mathbf{D}^{(k)}_v | \mathbb{X}^{(k)}(u) = \beta; T, \mathbf{\Theta}) \\
&\leq \mathcal{P}(\mathbb{D}^{(k)}(l) = \mathbf{D}^{(k)}(l) | \mathbb{X}^{(k)}(u) = \beta; T, \mathbf{\Theta}) \\
&= \mathcal{P}(\mathbb{D}^{(k)}(l) = \alpha | \mathbb{X}^{(k)}(u) = \beta; T, \mathbf{\Theta}) \\
&= \sum_{\gamma} \mathcal{P}(\mathbb{D}^{(k)}(l) = \alpha, \mathbb{X}^{(k)}(l) = \gamma | \mathbb{X}^{(k)}(u) = \beta; T, \mathbf{\Theta}) \\
&= \sum_{\gamma} \mathcal{P}(\mathbb{D}^{(k)}(l) = \alpha | \mathbb{X}^{(k)}(l) = \gamma; T, \mathbf{\Theta}) \mathcal{P}(\mathbb{X}^{(k)}(l) = \gamma | \mathbb{X}^{(k)}(u) = \beta; T, \mathbf{\Theta}) \\
&\leq \sum_{\gamma} \mathbf{\Phi}(\gamma, \alpha) \mathbf{\Psi}_{e_1}(\beta, \gamma) = 0
\end{aligned}$$

Proof of (3) From Eq. S10, for all $(\beta, \gamma) \in (\mathscr{A}^{(k)})^2 \setminus \{(0,0), (0,\alpha), (\alpha,\alpha)\}$, we have:

$$\mathcal{S}^{(k)}_{\text{edge}}(e, \beta, \gamma; T, \mathbf{\Theta}) = \frac{\mathcal{S}^{(k)}_{\text{node}}(u, \beta; T, \mathbf{\Theta})\mathcal{L}^{(k)}_{\text{node}}(v, \gamma; T, \mathbf{\Theta})\mathbf{\Psi}^{(k)}_e(\beta, \gamma)}{\mathcal{L}^{(k)}_{\text{edge}}(e, \beta; T, \mathbf{\Theta})}$$

Consider the following exclusive cases:

- Case 1: $\gamma = 0$, then $\beta \neq 0$ (because $(\beta, \gamma) \neq (0, 0)$). It is followed that $\mathbf{\Psi}^{(k)}_e(\beta, \gamma) = \mathbf{\Psi}^{(k)}_e(\beta, 0) = 0$, so $\mathcal{S}^{(k)}_{\text{edge}}(e, \beta, \gamma; T, \mathbf{\Theta})$.
- Case 2: $\gamma = \alpha$, then $\beta \neq \alpha$ (because $(\beta, \gamma) \neq (\alpha, \alpha)$). It is also followed that $\mathbf{\Psi}^{(k)}_e(\beta, \gamma) = 0$, so $\mathcal{S}^{(k)}_{\text{edge}} = 0$
- Case 3: $\gamma \notin \{0, \alpha\}$. Using Statement (1), we have $\mathcal{L}^{(k)}_{\text{node}}(v, \gamma; T, \mathbf{\Theta}) = 0$, so $\mathcal{S}^{(k)}_{\text{edge}}(e, \beta, \gamma; T, \mathbf{\Theta}) = 0$.

Thus, $\mathcal{S}^{(k)}_{\text{edge}}(e, \beta, \gamma; T, \mathbf{\Theta}) = 0$ for all $(\beta, \gamma) \in (\mathscr{A}^{(k)})^2 \setminus \{(0,0), (0,\alpha), (\alpha,\alpha)\}$. □

S2.5.2 The linear-time algorithm

Recall that at iteration $t+1$ of the EM algorithm, we need to compute the posterior probabilities that are parameterized by $\mathbf{\Theta}^t$, where $\mathbf{\Theta}^t$ is the estimate at iteration t. Below we describe a new algorithm for the E-step that has lower complexity than that presented in the main text, using the special properties of α-clades and z-branches. Because the tree topology T and parameter $\mathbf{\Theta}^t$ are shared for all entities described below, in the rest of this section, we will drop these two parameters in all notations. Let

$$\mathcal{C}^{z\to z}_{e,k} = \mathcal{S}^{(k)}_{\text{edge}}(e, 0, 0)\ ; \mathcal{C}^{z\to a}_{e,k} = \sum_{\alpha \in \mathscr{A}^{(k)} \setminus \{0,-1,?\}} \mathcal{S}^{(k)}_{\text{edge}}(e, 0, \alpha)\ ; \mathcal{C}^{z\to m}_{e,k} = \mathcal{S}^{(k)}_{\text{edge}}(e, 0, -1)$$

$$\mathcal{C}^{a\to a}_{e,k} = \sum_{\alpha \in \mathscr{A}^{(k)} \setminus \{0,-1,?\}} \mathcal{S}^{(k)}_{\text{edge}}(e, \alpha, \alpha)\ ; \mathcal{C}^{a\to m}_{e,k} = \sum_{\alpha \in \mathscr{A}^{(k)} \setminus \{0,-1,?\}} \mathcal{S}^{(k)}_{\text{edge}}(e, \alpha, -1)$$

From Eq. 13 (see the main text) and the above equations, it is easy to see that:

$$\mathcal{C}^{z\to z}_e = \sum_{k=1}^{K} \mathcal{C}^{z\to z}_{e,k}$$

$$\mathcal{C}^{z\to a}_e = \sum_{k=1}^{K} \mathcal{C}^{z\to a}_{e,k}$$

$$\mathcal{C}^{a\to a}_e = \sum_{k=1}^{K} \mathcal{C}^{a\to a}_{e,k}$$

$$\mathcal{C}^{a\to m}_e = \sum_{k=1}^{K} \mathcal{C}^{a\to m}_{e,k}$$

Let $p_e = e^{-\delta_e}$. Using theorems 2, 3, and 4, we will prove the followings:

Casework for $\mathcal{C}_{e,k}^{z\to z}$

$$\mathcal{C}_{e,k}^{z\to z} = \begin{cases} 1, \text{ if } e \text{ is a z-branch at site } k \\ \mathcal{S}_{\text{node}}^{(k)}(u,0)\frac{\mathcal{L}_{\text{node}}^{(k)}(v,0)p_e^{1+\nu}}{\mathcal{L}_{\text{edge}}^{(k)}(e,0)}, \text{ otherwise} \end{cases}$$

Proof. If e is a z-branch, Eq. S2.5.2 is a direct corollary of Statement (3) of Theorem 2. Otherwise, from Eq. S10 we have:

$$\mathcal{C}_{e,k}^{z\to z} = \mathcal{S}_{\text{edge}}^{(k)}(e,0,0) = \frac{\mathcal{S}_{\text{node}}^{(k)}(u,0)\mathcal{L}_{\text{node}}^{(k)}(v,0)\mathbf{\Psi}_e^{(k)}(0,0)}{\mathcal{L}_{\text{edge}}^{(k)}(e,0)} = \mathcal{S}_{\text{node}}^{(k)}(u,0)\frac{\mathcal{L}_{\text{node}}^{(k)}(v,0)p_e^{1+\nu}}{\mathcal{L}_{\text{edge}}^{(k)}(e,0)}$$

□

Casework for $\mathcal{C}_{e,k}^{z\to a}$

$$\mathcal{C}_{e,k}^{z\to a} = \begin{cases} 0, \text{ if } e \text{ is a z-branch at site } k \\ \mathcal{S}_{\text{node}}^{(k)}(u,0) - \frac{\mathcal{S}_{\text{node}}^{(k)}(u,0)\mathcal{L}_{\text{node}}^{(k)}(v,0)}{\mathcal{L}_{\text{edge}}^{(k)}(e,0)}, \text{ if } e \text{ is unmasked at } i \\ \mathcal{S}_{\text{node}}^{(k)}(u,0) - \frac{\mathcal{S}_{\text{node}}^{(k)}(u,0)}{\mathcal{L}_{\text{edge}}^{(k)}(e,0)}(\mathcal{L}_{\text{node}}^{(k)}(v,0)p_e^{1+\nu} + (1-p_e^{\nu})), \text{ otherwise} \end{cases}$$

Proof. From the definition of $\mathcal{C}_{e,k}^{z\to a}$ and Eq. S10, we have:

$$\begin{aligned}\mathcal{C}_{e,k}^{z\to a} &= \textstyle\sum_{\alpha\in\mathscr{A}^{(k)}\setminus\{0,-1,?\}} \mathcal{S}_{\text{edge}}^{(k)}(e,0,\alpha) \\ &= \frac{\mathcal{S}_{\text{node}}^{(k)}(u,0)}{\mathcal{L}_{\text{edge}}^{(k)}(e,0)} \textstyle\sum_{\alpha\in\mathscr{A}^{(k)}\setminus\{0,-1,?\}} \mathcal{L}_{\text{node}}^{(k)}(v,\alpha)\mathbf{\Psi}_e^{(k)}(0,\alpha)\end{aligned}$$

If e is a z-branch, then according to Theorem 2, $\mathcal{L}_{\text{node}}^{(k)}(v,\alpha) = 0$ for all $\alpha \in \mathscr{A}^{(k)} \setminus \{0,-1,?\}$, so $\mathcal{C}_{e,k}^{z\to a} = 0$. Otherwise, we have:

$$\begin{aligned}&\sum_{\alpha\in\mathscr{A}^{(k)}\setminus\{0,-1,?\}} \mathcal{L}_{\text{node}}^{(k)}(v,\alpha)\mathbf{\Psi}_e^{(k)}(0,\alpha) \\ &= \sum_{\alpha\in\mathscr{A}^{(k)}\setminus\{0,-1,?\}} \mathcal{P}(\mathbf{D}_v^{(k)}|\mathbb{X}^{(k)}(v)=\alpha)\mathcal{P}(\mathbb{X}^{(k)}(v)=0|\mathbb{X}^{(k)}(u)=0) \\ &= \mathcal{P}(\mathbf{D}_v^{(k)}|\mathbb{X}^{(k)}(u)=0) - \mathcal{P}(\mathbf{D}_v^{(k)}|\mathbb{X}^{(k)}(v)=0)\mathcal{P}(\mathbb{X}^{(k)}(v)=0|\mathbb{X}^{(k)}(u)=0) \\ &- \mathcal{P}(\mathbf{D}_v^{(k)}|\mathbb{X}^{(k)}(v)=-1)\mathcal{P}(\mathbb{X}^{(k)}(v)=-1|\mathbb{X}^{(k)}(u)=0) \\ &= \mathcal{L}_{\text{edge}}^{(k)}(e,0) - \mathcal{L}_{\text{node}}^{(k)}(v,0)\mathbf{\Psi}_e(0,0) - \mathcal{L}_{\text{node}}^{(k)}(v,-1)\mathbf{\Psi}_e(0,-1)\end{aligned}$$

If e is a masked branch, then $\mathcal{L}_{\text{node}}^{(k)}(v,-1) = 1$; otherwise, e is unmasked and is not a z-branch, so $\mathcal{L}_{\text{node}}^{(k)}(v,-1) = 0$ according to Theorem 4. Also, $\mathbf{\Psi}_e(0,-1) = 1 - p_e^{\nu}$ by definition. Thus, from Eq. S2.5.2 and S2.5.2, we get the proposed equation to compute $\mathcal{C}_{e,k}^{z\to a}$. □

Casework for $\mathcal{C}_{e,k}^{z\to m}$

$$\mathcal{C}_{e,k}^{z\to m} = \begin{cases} \mathcal{S}_{\textsf{node}}^{(k)}(u,0)\frac{1-p_e^{\nu}}{\mathcal{L}_{\textsf{edge}}^{(k)}(e,0)}, \text{ if } e \text{ is a masked-branch} \\ 0, \text{ otherwise} \end{cases}$$

Proof. From the definition of $\mathcal{C}_{e,k}^{z\to m}$ and Eq. S10, we have:

$$\begin{aligned} \mathcal{C}_{e,k}^{z\to m} &= \mathcal{S}_{\textsf{edge}}^{(k)}(e,0,-1) \\ &= \frac{\mathcal{S}_{\textsf{node}}^{(k)}(u,0)\mathcal{L}_{\textsf{node}}^{(k)}(v,-1)\mathbf{\Psi}_e^{(k)}(0,-1)}{\mathcal{L}_{\textsf{edge}}^{(k)}(e,0)} \end{aligned}$$

If e is not a masked branch, then according to Theorem 2 and Theorem 4, $\mathcal{L}_{\textsf{node}}^{(k)}(v,-1)=0$, so $\mathcal{C}_{e,k}^{z\to m}=0$. Otherwise, if e is a masked branch, then $\mathcal{L}_{\textsf{node}}^{(k)}(v,-1)=1$ (Theorem 3), and $\mathbf{\Psi}_e^{(k)}(0,-1)=1-p_e^{\nu}$. From here we get the proposed equation. □

Casework for $\mathcal{C}_{e,k}^{a\to a}$

$$\mathcal{C}_{e,k}^{a\to a} = \begin{cases} 0, \text{ if e is a z-branch at site } k \\ \frac{\mathcal{L}_{\textsf{node}}^{(k)}(v,\alpha_0)p_e^{\nu}}{\mathcal{L}_{\textsf{edge}}^{(k)}(e,\alpha_0)}\mathcal{S}_{\textsf{node}}^{(k)}(u,\alpha_0), \text{ if e is an unmasked branch of } \alpha_0\text{-clade at } k \\ \dfrac{\mathcal{L}_{\textsf{node}}^{(k)}(v,\alpha_0)p_e^{\nu}}{\mathcal{L}_{\textsf{edge}}^{(k)}(e,\alpha_0)}(1-\mathcal{S}_{\textsf{node}}^{(k)}(u,0)-\mathcal{S}_{\textsf{node}}^{(k)}(u,-1)), \text{ if } e \text{ is a masked branch, } \alpha_0 \text{ is arbitrarily chosen in } \mathscr{A}^{(k)}\setminus\{0, \end{cases}$$

Proof.

$$\begin{aligned} \mathcal{C}_{e,k}^{a\to a} &= \sum_{\alpha\in\mathscr{A}^{(k)}\setminus\{0,-1,?\}} \mathcal{S}_{\textsf{edge}}^{(k)}(e,\alpha,\alpha) \\ &= \sum_{\alpha\in\mathscr{A}^{(k)}\setminus\{0,-1,?\}} \frac{\mathcal{S}_{\textsf{node}}^{(k)}(u,\alpha)\mathcal{L}_{\textsf{node}}^{(k)}(v,\alpha)\mathbf{\Psi}_e^{(k)}(\alpha,\alpha)}{\mathcal{L}_{\textsf{edge}}^{(k)}(e,\alpha)} \\ &= \sum_{\alpha\in\mathscr{A}^{(k)}\setminus\{0,-1,?\}} \frac{\mathcal{S}_{\textsf{node}}^{(k)}(u,\alpha)\mathcal{L}_{\textsf{node}}^{(k)}(v,\alpha)p_e^{\nu}}{\mathcal{L}_{\textsf{edge}}^{(k)}(e,\alpha)} \end{aligned}$$

Case 1: If e is a z-branch, then $\mathcal{S}_{\textsf{node}}^{(k)}(u,\alpha)=0$ for all $\alpha\in\mathscr{A}^{(k)}\setminus\{0,-1,?\}$ (Theorem 2), so $\mathcal{C}_{e,k}^{a\to a}=0$.
Case 2: If e is an unmasked branch of some α_0-clade, then $\mathcal{P}(\mathbf{D}_v^{(k)}|\mathbb{X}^{(k)}(v)=\alpha)=0$ for all $\alpha\in\mathscr{A}^{(k)}\setminus\{0,-1,?,\alpha_0\}$ (Theorem 4), so the summation is reduced to the only term containing α_0. Thus:

$$\mathcal{C}_{e,k}^{a\to a} = \frac{\mathcal{L}_{\textsf{node}}^{(k)}(v,\alpha_0)p_e^{\nu}}{\mathcal{L}_{\textsf{edge}}^{(k)}(e,\alpha_0)}\mathcal{S}_{\textsf{node}}^{(k)}(u,\alpha_0)$$

Case 3: If e is a masked branch, then from Statements (2) and (3) of Theorem 3, we have:

$$\begin{aligned}\mathcal{C}_{e,k}^{a\to a} &= \frac{\mathcal{L}_{\text{node}}^{(k)}(v,\alpha_0)p_e^{\nu}}{\mathcal{L}_{\text{edge}}^{(k)}(e,\alpha_0)} \sum_{\alpha\in\mathscr{A}^{(k)}\setminus\{0,-1,?\}} \mathcal{S}_{\text{node}}^{(k)}(u,\alpha) \\ &= \frac{\mathcal{L}_{\text{node}}^{(k)}(v,\alpha_0)p_e^{\nu}}{\mathcal{L}_{\text{edge}}^{(k)}(e,\alpha_0)}(1-\mathcal{S}_{\text{node}}^{(k)}(u,0)-\mathcal{S}_{\text{node}}^{(k)}(u,-1)),\end{aligned}$$

where α_0 is arbitrarily chosen in $\mathscr{A}^{(k)}\setminus\{0,-1,?\}$ □

Casework for $\mathcal{C}_{e,k}^{a\to m}$

$$\mathcal{C}_{e,k}^{a\to m} = \begin{cases} \dfrac{1-p_e^{\nu}}{\mathcal{L}_{\text{edge}}^{(k)}(e,\alpha_0)}(1-\mathcal{S}_{\text{node}}^{(k)}(u,0)-\mathcal{S}_{\text{node}}^{(k)}(u,-1)), & \text{if } e \text{ is a masked branch, } \alpha_0 \text{ is arbitrarily chosen in } \mathscr{A}^{(k)}\setminus \\ 0, & \text{otherwise} \end{cases}$$

Proof. From the definition of $\mathcal{C}_{e,k}^{a\to m}$ and Eq. S10, we have:

$$\begin{aligned}\mathcal{C}_{e,k}^{a\to m} &= \sum_{\alpha\in\mathscr{A}^{(k)}\setminus\{0,-1,?\}} \mathcal{S}_{\text{edge}}^{(k)}(e,\alpha,-1) \\ &= \sum_{\alpha\in\mathscr{A}^{(k)}\setminus\{0,-1,?\}} \frac{\mathcal{S}_{\text{node}}^{(k)}(u,\alpha)\mathcal{L}_{\text{node}}^{(k)}(v,-1)\mathbf{\Psi}_e^{(k)}(\alpha,-1)}{\mathcal{L}_{\text{edge}}^{(k)}(e,\alpha)}\end{aligned}$$

If e is not a masked branch, then according to Theorem 2 and Theorem 4, $\mathcal{L}_{\text{node}}^{(k)}(v,-1)=0$, so $\mathcal{C}_{e,k}^{a\to m}=0$. Otherwise, if e is a masked branch, then $\mathcal{L}_{\text{node}}^{(k)}(v,-1)=1$ (Theorem 3), $\mathbf{\Psi}_e^{(k)}(\alpha,-1)=1-p_e^{\nu}$ for all $\alpha\in\mathscr{A}^{(k)}\setminus\{0,-1,?\}$. Combining with Statements (2) and (3) of Theorem 3, we have:

$$\begin{aligned}\mathcal{C}_{e,k}^{a\to m} &= \frac{1-p_e^{\nu}}{\mathcal{L}_{\text{edge}}^{(k)}(e,\alpha_0)} \sum_{\alpha\in\mathscr{A}^{(k)}\setminus\{0,-1,?\}} \mathcal{S}_{\text{node}}^{(k)}(u,\alpha) \\ &= \frac{1-p_e^{\nu}}{\mathcal{L}_{\text{edge}}^{(k)}(e,\alpha_0)}(1-\mathcal{S}_{\text{node}}^{(k)}(u,0)-\mathcal{S}_{\text{node}}^{(k)}(u,-1)),\end{aligned}$$

where α_0 is arbitrarily chosen in $\mathscr{A}^{(k)}\setminus\{0,-1,?\}$ □

Thus, for any branch $e=(u,v)$, if e is a z-branch of site k, then each of the 5 entities $\mathcal{C}_{e,k}^{z\to z}$, $\mathcal{C}_{e,k}^{z\to a}$, $\mathcal{C}_{e,k}^{z\to m}$, $\mathcal{C}_{e,k}^{a\to a}$, $\mathcal{C}_{e,k}^{a\to m}$ is either 0 or 1 (Theorem 2). Otherwise, to compute them we need the following 6 entities:

- $\mathcal{L}_{\text{edge}}^{(k)}(e,0)$
- $\mathcal{L}_{\text{edge}}^{(k)}(e,\alpha_0)$
- $\mathcal{L}_{\text{node}}^{(k)}(v,0)$
- $\mathcal{L}_{\text{node}}^{(k)}(v,\alpha_0)$
- $\mathcal{S}_{\text{node}}^{(k)}(u,0)$
- $\mathcal{S}_{\text{node}}^{(k)}(u,-1)$,

where α_0 is arbitrarily chosen in $\mathscr{A}^{(k)}\setminus\{0,-1\}$ if e is a masked-branch; otherwise, e belongs to an α-clade where α is uniquely defined and we let $\alpha_0=\alpha$.

Computing$\mathcal{L}^{(k)}_{\text{edge}}(e,0)$***and***$\mathcal{L}^{(k)}_{\text{edge}}(e,\alpha_0)$

Base case: if v is a leaf node, then

$$\mathcal{L}^{(k)}_{\text{edge}}(e,0) = \begin{cases} \phi p_e^{\nu} - p_e^{\nu} + 1, & \text{if } e \text{ is a masked branch} \\ q_{\alpha_0} p_e^{\nu}(1-p_e)(1-\phi), & \text{otherwise} \end{cases}$$

$$\mathcal{L}^{(k)}_{\text{edge}}(e,\alpha_0) = \begin{cases} \phi p_e^{\nu} - p_e^{\nu} + 1, & \text{if } e \text{ is a masked branch} \\ p_e^{\nu}(1-\phi), & \text{otherwise} \end{cases}$$

Recursion: if v has two children, v_1 and v_2, then let $e_1 = (v, v_1), e_2 = (v, v_2)$, we have:

$$\mathcal{L}^{(k)}_{\text{edge}}(e,0) = \begin{cases} \mathcal{L}^{(k)}_{\text{edge}}(e_1,0)\mathcal{L}^{(k)}_{\text{edge}}(e_2,0)p_e^{\nu+1} + \mathcal{L}^{(k)}_{\text{edge}}(e_1,\alpha_0)\mathcal{L}^{(k)}_{\text{edge}}(e_2,\alpha_0)(1-p_e)p_e^{\nu} + (1-p_e^{\nu}), & \text{if } e \text{ is masked} \\ \mathcal{L}^{(k)}_{\text{edge}}(e_1,0)\mathcal{L}^{(k)}_{\text{edge}}(e_2,0)p_e^{\nu+1} + \mathcal{L}^{(k)}_{\text{edge}}(e_1,\alpha_0)\mathcal{L}^{(k)}_{\text{edge}}(e_2,\alpha_0)q_{\alpha_0}(1-p_e)p_e^{\nu}, & \text{otherwise} \end{cases}$$

Computing $\mathcal{L}^{(k)}_{\text{node}}(v,0)$ ***and*** $\mathcal{L}^{(k)}_{\text{node}}(v,\alpha_0)$

$$\mathcal{L}^{(k)}_{\text{node}}(v,0) = \begin{cases} \phi, & \text{if v is a leaf and } \mathbf{D}^{(k)}(v) = ? \\ 1-\phi, & \text{if v is a leaf and } \mathbf{D}^{(k)}(v) = 0 \\ 0, & \text{if v is a leaf and } \mathbf{D}^{(k)}(v) \notin \{0, ?\} \\ \mathcal{L}^{(k)}_{\text{edge}}(e_1,0)\mathcal{L}^{(k)}_{\text{edge}}(e_2,0), & \text{if v has two children, } v_1 \text{ and } v_2;\ e_1 = (v,v_1),\ e_2 = (v,v_2) \end{cases}$$

$$\mathcal{L}^{(k)}_{\text{node}}(v,\alpha_0) = \begin{cases} \phi, & \text{if v is a leaf and } \mathbf{D}^{(k)}(v) = ? \\ 1-\phi, & \text{if v is a leaf and } \mathbf{D}^{(k)}(v) = \alpha_0 \\ 0, & \text{if v is a leaf and } \mathbf{D}^{(k)}(v) \notin \{\alpha_0, ?\} \\ \mathcal{L}^{(k)}_{\text{edge}}(e_1,\alpha_0)\mathcal{L}^{(k)}_{\text{edge}}(e_2,\alpha_0), & \text{if v has two children, } v_1 \text{ and } v_2;\ e_1 = (v,v_1),\ e_2 = (v,v_2) \end{cases}$$

Computing $\mathcal{S}^{(k)}_{\text{node}}(u,0)$ ***and*** $\mathcal{S}^{(k)}_{\text{node}}(u,-1)$**.**

Consider a branch (u, v). From Eq. (S8), we have: if u is the root, then $\mathcal{S}^{(k)}_{\text{node}}(u,0) = 1$ and $\mathcal{S}^{(k)}_{\text{node}}(u,-1) = 0$. Otherwise, to compute these two entities we need 5 other entities: (1) $\mathcal{L}^{(k)}_{\text{node}}(u,0)$, (2) $\mathcal{L}^{(k)}_{\text{node}}(u,-1)$, (3) $\mathcal{P}(\mathbf{D}^{(k)})$, (4) $\mathcal{L}^{(k)}_{\text{out}}(u,0)$, and (5) $\mathcal{L}^{(k)}_{\text{out}}(u,-1)$. Among them, (1) and (2) are already computed (see the above section). Entity (3) is simply the full likelihood of site k, so it is equal to $\mathcal{L}^{(k)}_{\text{edge}}(e_0,0)$ where e_0 is the root branch. To compute (4) and (5) (i.e. the outside likelihoods), we can use Eq. (S6), which gives a top-down recursive formula. Let u_0 be the parent of u, $e = (u_0, u)$ be the branch connecting u_0 and u, and u_1 be the sister of u (if there exists one) and $e_1 = (u_0, u_1)$. From Eq. (S6), it is straightforward to derive the following:

$$\mathcal{L}^{(k)}_{\text{out}}(u,0) = \begin{cases} p_e^{1+\nu}, & \text{if } e \text{ is the root branch} \\ p_e^{1+\nu}\mathcal{L}^{(k)}_{\text{edge}}(e_1,0)\mathcal{L}^{(k)}_{\text{out}}(u_0,0), & \text{otherwise} \end{cases}$$

Thus, we have a top-down recursion to compute $\mathcal{L}^{(k)}_{\text{out}}(u,0)$. The computation of $\mathcal{L}^{(k)}_{\text{out}}(u,-1)$, however, is more complicated. A naive application of Eq. (S6) requires the outside likelihoods with respect to all states β at the parent of u. Therefore, it takes $\mathcal{O}(M)$ time to compute $\mathcal{L}^{(k)}_{\text{out}}(u,-1)$ for each node u, so the overall complexity is $\mathcal{O}(NKM)$ where M is the maximum

alphabet size. Nevertheless, we can still employ the special properties of the model to reduce the algorithm to $\mathcal{O}(NK)$, as shown below.

Let v be the parent of u, $e = (v, u) \in \mathscr{E}_T$ be the branch connecting v and u, w be the sister of u, and $e_1 = (v, w)$. Using Eq. S6, it is straight forward to prove the followings:

- If e_1 is a z-branch, then $\mathcal{L}^{(k)}_{\mathsf{edge}}(e_1, \beta) = 0$ for all $\beta \neq 0$. Therefore, $\mathcal{L}^{(k)}_{\mathsf{out}}(u, -1) = (1 - p_e^\nu)\mathcal{L}^{(k)}_{\mathsf{out}}(v, 0)\mathcal{L}^{(k)}_{\mathsf{edge}}(e_1, -1)$
- If e_1 is an unmasked branch of an α_0-clade, then $\mathcal{L}^{(k)}_{\mathsf{edge}}(e_1, \beta) = 0$ for all $\beta \notin \{0, \alpha_0\}$. Therefore, $\mathcal{L}^{(k)}_{\mathsf{out}}(u, -1) = (1 - p_e^\nu)\mathcal{L}^{(k)}_{\mathsf{out}}(v, 0)\mathcal{L}^{(k)}_{\mathsf{edge}}(e_1, 0) + (1 - p_e^\nu)\mathcal{L}^{(k)}_{\mathsf{out}}(v, \alpha_0)\mathcal{L}^{(k)}_{\mathsf{edge}}(e_1, \alpha_0)$
- If e_1 is a masked branch, then $\mathcal{L}^{(k)}_{\mathsf{edge}}(e_1, \alpha) = \mathcal{L}^{(k)}_{\mathsf{edge}}(e_1, \beta)$ for all $\alpha \neq \beta$, $\alpha, \beta \in \mathscr{A}^{(k)} \setminus \{0, -1, ?\}$ (Theoreom 3). Let α_0 be an arbitrarily chosen character state in $\mathscr{A}^{(k)} \setminus \{0, -1, ?\}$. Then $\mathcal{L}^{(k)}_{\mathsf{out}}(u, -1) = (1 - p_e^\nu)\mathcal{L}^{(k)}_{\mathsf{out}}(v, 0)\mathcal{L}^{(k)}_{\mathsf{edge}}(e_1, 0) + \mathcal{L}^{(k)}_{\mathsf{out}}(v, -1)\mathcal{L}^{(k)}_{\mathsf{edge}}(e_1, -1) + (1 - p_e^\nu)\mathcal{L}^{(k)}_{\mathsf{edge}}(e_1, \alpha_0)M_v$, where $M_v = \sum_{\beta \notin \{0,-1\}} \mathcal{L}^{(k)}_{\mathsf{out}}(v, \beta)$.

From the above equations, we see that in all cases $\mathcal{L}^{(k)}_{\mathsf{out}}(u, -1)$ can be computed from $\mathcal{L}^{(k)}_{\mathsf{out}}(v, \alpha_0)$, M_v, and other known entities. Therefore, what left to be done is deriving top-down recursive formulas for $\mathcal{L}^{(k)}_{\mathsf{out}}(v, \alpha_0)$ and M_v. Below we show the formulas and leave the derivation to the reader.

Recursive formula for M_v.

Consider a branch $e = (u, v)$, let w be the sister of v and $e_1 = (u, w)$. Recall that by definition, $M_v = \sum_{\beta \notin \{0,-1\}} \mathcal{L}^{(k)}_{\mathsf{out}}(v, \beta)$. Below is the topdown recursive formula to compute M_v from M_u and other known entities:

$$M_v = \begin{cases} \mathcal{L}^{(k)}_{\mathsf{out}}(u, 0)\mathcal{L}^{(k)}_{\mathsf{edge}}(e_1, 0)p_e^\nu(1 - p_e), & \text{if } e_1 \text{ is a z-branch} \\ \mathcal{L}^{(k)}_{\mathsf{out}}(u, 0)\mathcal{L}^{(k)}_{\mathsf{edge}}(e_1, 0)p_e^\nu(1 - p_e) + p_e^\nu M_u \mathcal{L}^{(k)}_{\mathsf{edge}}(e_1, \alpha_0), & \text{if } e_1 \text{ is a masked branch} \\ \mathcal{L}^{(k)}_{\mathsf{out}}(u, 0)\mathcal{L}^{(k)}_{\mathsf{edge}}(e_1, 0)p_e^\nu(1 - p_e) + \mathcal{L}^{(k)}_{\mathsf{out}}(u, \alpha_0)\mathcal{L}^{(k)}_{\mathsf{edge}}(e_1, \alpha_0)p_e^\nu, & \text{otherwise} \end{cases}$$

Recursive formula for $\mathcal{L}^{(k)}_{\mathsf{out}}(v, \alpha_0)$.

Consider a branch $e = (u, v)$, let w be the sister of v and $e_1 = (u, w)$. For any $\alpha_0 \notin \{0, -1\}$, we have the following topdown recursive formula to compute $\mathcal{L}^{(k)}_{\mathsf{out}}(v, \alpha_0)$ from $\mathcal{L}^{(k)}_{\mathsf{out}}(u, 0)$, $\mathcal{L}^{(k)}_{\mathsf{out}}(u, \alpha_0)$, and other known entities:

$$\mathcal{L}^{(k)}_{\mathsf{out}}(v, \alpha_0) = \mathcal{L}^{(k)}_{\mathsf{out}}(u, 0)q_{\alpha_0}p_e^\nu(1 - p_e)\mathcal{L}^{(k)}_{\mathsf{edge}}(e_1, 0) + \mathcal{L}^{(k)}_{\mathsf{out}}(u, \alpha_0)p_e^\nu\mathcal{L}^{(k)}_{\mathsf{edge}}(e_1, \alpha_0)$$

S2.6 Solving the M-step

S2.6.1 Proof of convexity by variable blocks

Recall that at iteration t of the proposed EM algorithm, in the M-step we need to solve the following optimization problem:

$$\max_{\boldsymbol{\Theta}} f(\boldsymbol{\Theta};\boldsymbol{\Theta}^{t-1}) = \sum_{e=(u,v)\in\mathscr{E}_T} \big(-\mathcal{C}_e^{z\to z}(1+\nu)\delta_e + \mathcal{C}_e^{z\to a}(\log(1-e^{-\delta_e}) - \nu\delta_e) + \mathcal{C}_e^{z\to m}\log(1-e^{-\delta_e\nu}) - \mathcal{C}_e^{a\to a}\delta_e\nu + \mathcal{C}_e^{a\to m}\log(1-e^{-\delta_e\nu})\big) + \sum_{u\in\mathscr{L}_T}\Big(\mathcal{B}_u\log(1-\phi) + \tilde{\mathcal{B}}_u\log\phi\Big)$$

Below we prove that $f(\boldsymbol{\Theta};\boldsymbol{\Theta}^{t-1})$ is concave with respect to $\{\delta_e\}$, ν, and ϕ separately. Note that all variables are separable, so to prove concavity, we need to show that the second-order partial derivatives are negative. Indeed, we have:

$$\frac{\partial^2 f}{\partial\phi^2} = \sum_{u\in\mathscr{L}_T}\left(-\frac{\mathcal{B}_u}{(1-\phi)^2} - \frac{\tilde{\mathcal{B}}_u}{\phi^2}\right) < 0 \text{ for all } \phi\notin\{0,1\}$$

$$\frac{\partial^2 f}{\partial\nu^2} = \sum_{e\in\mathscr{E}_T} -(\mathcal{C}_e^{z\to m}+\mathcal{C}_e^{a\to m})\frac{e^{-\delta_e\nu}}{(1-e^{-\delta_e\nu})^2}\delta_e^2 < 0 \text{ for all } \nu\geq 0$$

$$\frac{\partial^2 f}{\partial\delta_e^2} = \sum_{e\in\mathscr{E}_T}\left(-\mathcal{C}_e^{z\to a}\frac{e^{-\delta_e}}{(1-e^{-\delta_e})^2} - (\mathcal{C}_e^{z\to m}+\mathcal{C}_e^{a\to m})\frac{e^{-\delta_e\nu}}{(1-e^{-\delta_e\nu})^2}\nu^2\right) < 0 \text{ for all } \delta_e\geq 0$$

S2.6.2 The block coordinate ascent algorithm

We initialize $\boldsymbol{\Theta}^t \leftarrow \boldsymbol{\Theta}^{t-1}$, then successively minimize f along the coordinate of ϕ, ν, and the block of $\{\delta_e\}$ while fixing the others, and iterate until convergence. In other words, let $\phi^{(t,1)} = \phi^{(t)}$, $\nu^{(t,1)} = \nu^{(t)}$, and $\{\delta\}^{(t,1)} = \{\delta\}^{(t)}$; at each iteration p of coordinate ascent, we find $\phi^{(t,p+1)}$, $\nu^{(t,p+1)}$, and $\{\delta\}^{(t,p+1)}$ such that:

$$\phi^{(t,p+1)} = \operatorname*{argmax}_{\phi}\sum_{u\in\mathscr{L}_T}\Big(\mathcal{B}_u\log(1-\phi)+\tilde{\mathcal{B}}_u\log\phi\Big) = \frac{\sum_{u\in\mathscr{L}_T}\tilde{\mathcal{B}}_u}{\sum_{u\in\mathscr{L}_T}(\mathcal{B}_u+\tilde{\mathcal{B}}_u)}$$

$$\nu^{(t,p+1)} = \operatorname*{argmax}_{\nu}\sum_{e\in\mathscr{E}_T}\Big(-\mathcal{C}_e^{z\to z}(1+\nu)\delta_e^{(t,p)} + \mathcal{C}_e^{z\to a}(\log(1-e^{-\delta_e^{(t,p)}}) - \nu\delta_e^{(t,p)}) + \mathcal{C}_e^{z\to m}\log(1-e^{-\delta_e^{(t,p)}\nu}) - \mathcal{C}_e^{a\to a}\delta_e^{(t,p)}\nu + \mathcal{C}_e^{a\to m}\log(1-e^{-\delta_e^{(t,p)}\nu})\Big)$$

$$\{\delta_e\}^{(t,p+1)} = \operatorname*{argmax}_{\{\delta_e\}}\sum_{e\in\mathscr{E}_T}\Big(-\mathcal{C}_e^{z\to z}(1+\nu^{(t,p+1)})\delta_e + \mathcal{C}_e^{z\to a}(\log(1-e^{-\delta_e}) - \nu^{(t,p+1)}\delta_e) + \mathcal{C}_e^{z\to m}\log(1-e^{-\delta_e\nu^{(t,p+1)}}) - \mathcal{C}_e^{a\to a}\delta_e\nu^{(t,p+1)} + \mathcal{C}_e^{a\to m}\log(1-e^{-\delta_e\nu^{(t,p+1)}})\Big)$$

While $\phi^{(t,p+1)}$ has a closed-form, $\nu^{(t,p+1)}$ and $\{\delta_e\}^{(t,p+1)}$ require solving convex optimization problems. We use the CVXPY package [60, 61] with the MOSEK solver [77] to solve them.
